# Cell-free, high-density lipoprotein–specific phospholipid efflux assay predicts incident cardiovascular disease

**DOI:** 10.1172/JCI165370

**Published:** 2023-09-15

**Authors:** Masaki Sato, Edward B. Neufeld, Martin P. Playford, Yu Lei, Alexander V. Sorokin, Angel M. Aponte, Lita A. Freeman, Scott M. Gordon, Amit K. Dey, Kianoush Jeiran, Masato Hamasaki, Maureen L. Sampson, Robert D. Shamburek, Jingrong Tang, Marcus Y. Chen, Kazuhiko Kotani, Josephine L.C. Anderson, Robin P.F. Dullaart, Nehal N. Mehta, Uwe J.F. Tietge, Alan T. Remaley

**Affiliations:** 1 Lipoprotein Metabolism Laboratory, National Heart, Lung, and Blood Institute (NHLBI), NIH, Bethesda, Maryland, USA.; 2 Division of Community and Family Medicine and Department of Clinical Laboratory Medicine, Jichi Medical University, Shimotsuke-City, Tochigi, Japan.; 3 Biochemical Research Laboratory II, Eiken Chemical Co., Ltd., Shimotsuga-gun, Tochigi, Japan.; 4 Section of Inflammation and Cardiometabolic Diseases, NHLBI, NIH, Bethesda, Maryland, USA.; 5 Division of Clinical Chemistry, Department of Laboratory Medicine, Karolinska Institute, Stockholm, Sweden.; 6 Proteomics Core Facility, NHLBI, NIH, Bethesda, Maryland, USA.; 7 Saha Cardiovascular Research Center and Department of Physiology, University of Kentucky, Lexington, Kentucky, USA.; 8 The NIH Clinical Center and; 9 Laboratory of Cardiovascular CT, NHLBI, NIH, Bethesda, Maryland, USA.; 10 Department of Internal Medicine, University Medical Center Groningen, University of Groningen, Groningen, Netherlands.; 11 Clinical Chemistry, Karolinska University Laboratory, Karolinska University Hospital, Stockholm, Sweden.

**Keywords:** Vascular Biology, Cardiovascular disease, Lipoproteins, Molecular diagnosis

## Abstract

**BACKGROUND:**

Cellular cholesterol efflux capacity (CEC) is a better predictor of cardiovascular disease (CVD) events than HDL-cholesterol (HDL-C) but is not suitable as a routine clinical assay.

**METHODS:**

We developed an HDL-specific phospholipid efflux (HDL-SPE) assay to assess HDL functionality based on whole plasma HDL apolipoprotein–mediated solubilization of fluorescent phosphatidylethanolamine from artificial lipid donor particles. We first assessed the association of HDL-SPE with prevalent coronary artery disease (CAD): study I included NIH severe-CAD (*n* = 50) and non-CAD (*n* = 50) participants, who were frequency matched for sex, BMI, type 2 diabetes mellitus, and smoking; study II included Japanese CAD (*n* = 70) and non-CAD (*n* = 154) participants. We also examined the association of HDL-SPE with incident CVD events in the Prevention of Renal and Vascular End-stage Disease (PREVEND) study comparing 340 patients with 340 controls individually matched for age, sex, smoking, and HDL-C levels.

**RESULTS:**

Receiver operating characteristic curves revealed stronger associations of HDL-SPE with prevalent CAD. The AUCs in study I were as follows: HDL-SPE, 0.68; apolipoprotein A-I (apoA-I), 0.62; HDL-C, 0.63; and CEC, 0.52. The AUCs in study II were as follows: HDL-SPE, 0.83; apoA-I, 0.64; and HDL-C, 0.53. Also longitudinally, HDL-SPE was significantly associated with incident CVD events independent of traditional risk factors with ORs below 0.2 per SD increment in the PREVEND study (*P* < 0.001).

**CONCLUSION:**

HDL-SPE could serve as a routine clinical assay for improving CVD risk assessment and drug discovery.

**TRIAL REGISTRATION:**

ClinicalTrials.gov NCT01621594.

**FUNDING:**

NHLBI Intramural Research Program, NIH (HL006095-06).

## Graphical abstract

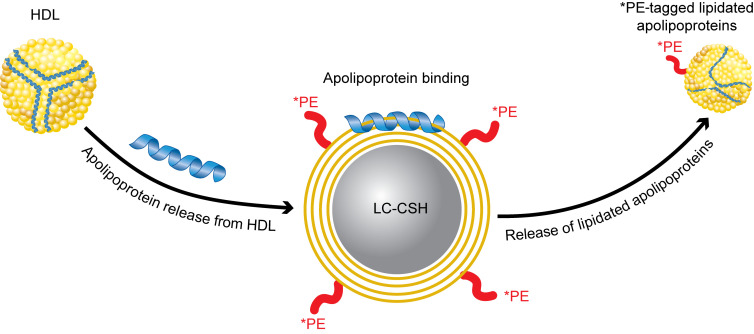

## Introduction

Coronary artery disease (CAD) risk assessment is a critical step for determining the need for lipid-lowering therapy (1). HDL-cholesterol (HDL-C) and total cholesterol are the 2 lipid tests currently used to calculate a 10-year CAD risk score for the US multi-society guidelines on cholesterol management (1). Low levels of HDL-C have consistently been shown to be inversely related to CAD in large epidemiological studies (2, 3), but recently, very high HDL-C has been found to be positively associated with incident CAD and all-cause mortality (4–8). In addition, pharmacologically-induced increases in HDL-C have not proven so far to be beneficial in preventing CAD events (9). For these reasons, interest has shifted to assessing HDL function as a metric for CAD risk (10).

HDL has a variety of potential antiatherogenic functions, but the best understood is its ability to remove excess cholesterol from lipid-laden macrophages in arterial lesions (10). This aspect of HDL functionality is typically measured by the cellular cholesterol efflux capacity (CEC) assay, which depends on the in vitro efflux of radioactive or fluorescent cholesterol to apolipoprotein B–depleted (apoB-depleted) serum from macrophages grown in cell culture. CEC has been shown to better predict prevalent CAD than HDL-C concentrations (11) and is inversely associated with future CAD events (12). A major limitation of the cell-based CEC assays is that they are not amenable for diagnostic testing in a routine clinical laboratory setting and are therefore limited to just research studies.

Recently, cell-free assays that monitor fluorescent cholesterol uptake capacity of HDL have been developed as potential CAD biomarkers (13, 14). One of these assays is based on the ability of apolipoprotein A-I (apoA-I), the main protein component of HDL, to exchange between lipid surfaces, which is known to highly correlate with ATP-binding cassette transporter A1–specific (ABCA1-specific) CEC (15). This assay utilizes electron paramagnetic resonance and was found to be markedly reduced in patients with acute coronary syndrome (16). In another cell-free HDL function assay, a dual NBD/Alexa Fluor 647–labeled exogenous apoA-I was used as a probe, revealing an association between a low apoA-I exchange rate and an increased risk of incident major adverse cardiac events (MACEs) (17). Surrogate assays based on plasma proteomics (18) and nuclear magnetic resonance–based (NMR-based) analyses (19) that closely correlate with CEC and CAD risk have also been described. None of these alternatives to the CEC assays has been implemented as a routine diagnostic test, either because of the complexity of the test or the need for a tedious sample preparation step involving the physical separation of HDL from other lipoproteins.

Although not as widely studied, phospholipids are also removed during the lipid efflux process from cells by exchangeable apolipoproteins (20–22). We recently by agarose gel analysis found that apoA-I or HDL can remove a fluorescence-tagged analog of phosphatidylethanolamine, a nonexchangeable lipid, from multilamellar vesicles composed of a mixture of phosphatidylcholine and cholesterol (23, 24). In the present study, we used calcium silicate hydrate crystals labeled with fluorescence-tagged phosphatidylethanolamine as artificial lipid-donor particles to develop a high-throughput cell-free, HDL-specific phospholipid efflux assay (HDL-SPE) that measures plasma HDL–associated lipid fluorescence in the supernatant and is suitable for automation. We found that the HDL-SPE assay depended on the binding of endogenous plasma apoA-I and other exchangeable apolipoproteins to the artificial lipid-donor particles and their subsequent dissociation along with solubilized phospholipids. We also compared our new HDL-SPE assay with cell-based cholesterol efflux assays and, more important, have examined its association with atherosclerotic cardiovascular disease (ASCVD) clinical events. In 3 different human studies, we demonstrate the potential clinical relevance of the new HDL-SPE assay by demonstrating substantial associations even after adjustment for other measures of HDL with prevalent CAD, as well as with incident CAD events. Taken together, our proof-of-principle studies indicate that HDL-SPE is a functional assay for HDL that is amenable for automation and could potentially be implemented as a routine diagnostic test for improving cardiovascular risk stratification.

## Results

### Development of a cell-free phospholipid efflux assay.

**Figure 1:**
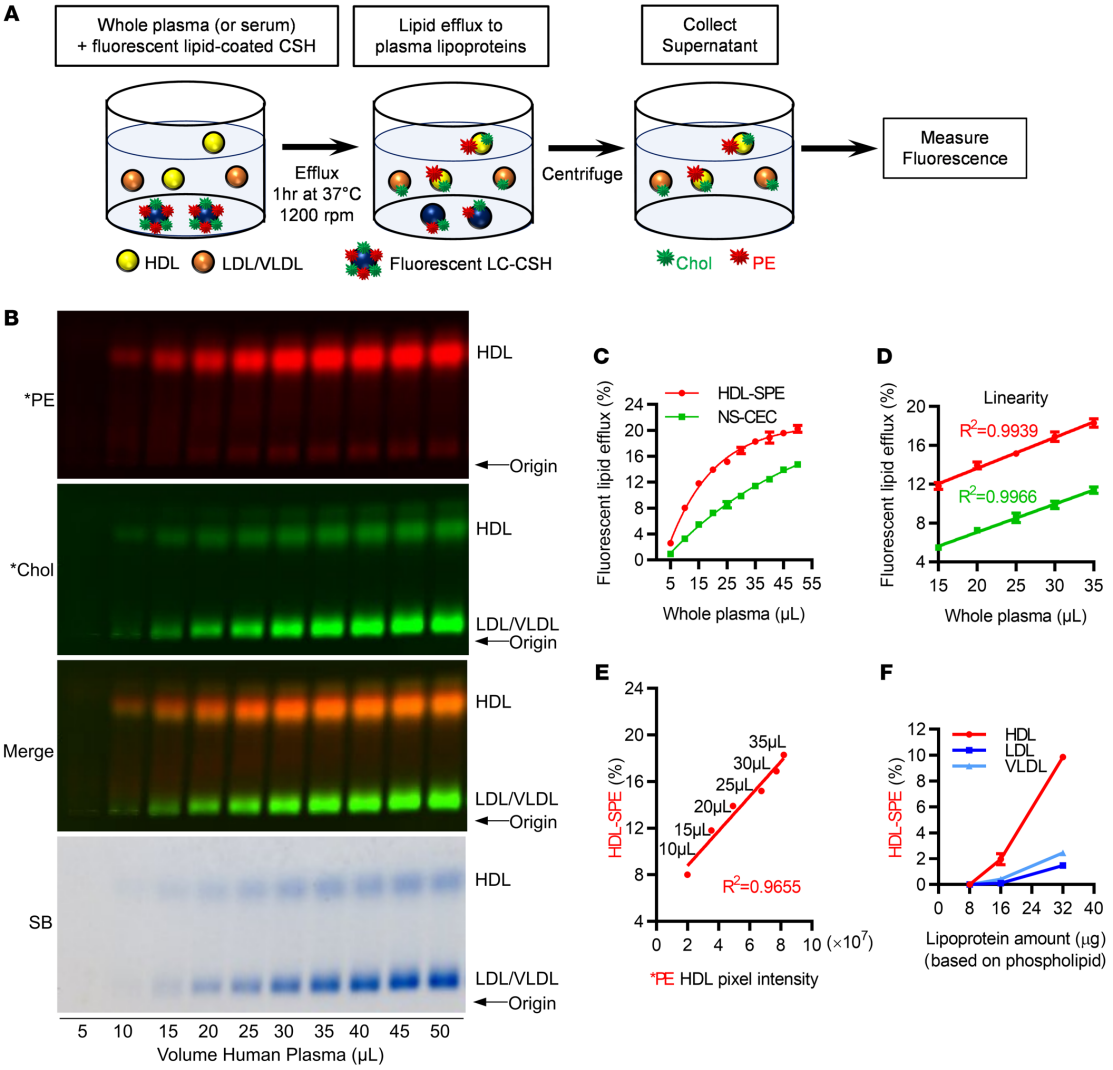
Efflux of fluorescent PE from donor LC-CSH particles to plasma lipoproteins is HDL specific. (**A**) Model of cell-free HDL-SPE and NS-CEC assays, as described in Methods. After centrifugation, lipoprotein-associated fluorescence in the supernatant is measured by agarose gel electrophoresis and fluorometry. HDL acquires both *PE and *Chol, whereas LDL/VLDL acquires only *Chol. (**B**) Effect of pooled normal HP volume on fluorescent lipid efflux. Agarose gel electrophoresis: *PE transfer from donor particles was HDL specific, whereas *Chol transfer to plasma lipoproteins was nonspecific. (**C**) Fluorometry of the reaction mixture supernatant revealed that the dependence of *PE and *Chol efflux on plasma volume differed. (**D**) Both HDL-SPE and NS-CEC were linear using 15–35 μL plasma. (**E**) Fluorometric and electrophoretic gel analyses of HDL-SPE were highly correlated. (**F**) LC-CSH *PE robustly effluxed to isolated HDL, whereas little to no *PE effluxed to isolated LDL or VLDL. All data are presented as the mean ± SD from triplicate assays unless otherwise stated. A *P* value of less than 0.05 was considered statistically significant. SUP, supernatant; R, released.

We used calcium silicate hydrate (CSH) crystals to make artificial lipid-donor particles, because of their high affinity for lipids and their high density, which allow for their easy removal by low-speed centrifugation in the last step of the assay. The final preparation of lipid-coated CSH (LC-CSH) particles used in our assay (Supplemental Figure 1; supplemental material available online with this article; https://doi.org/10.1172/JCI165370DS1) were made with a mixture of dimyristoylphosphatidylcholine and cholesterol (2:1 mole ratio), along with a trace amount of lissamine rhodamine-tagged phosphatidylethanolamine (*PE). Confocal and electron microscopy revealed that LC-CSH particles are plate- and needle-like lipid-coated structures (Supplemental Figure 2). The solubilization of phospholipids from the lipid-donor particles by HDL in plasma, which we refer to as HDL-SPE, was monitored by the measurement of fluorescent *PE in the supernatant after removal of LC-CSH particles by low-speed centrifugation (Figure 1A).

We initially conducted a large series of experiments to first optimize the type of LC-CSH particle that would still provide a good signal in our studies but would have a relatively simple lipid composition. As detailed in Supplemental Figure 1, 1,2-dimyristoyl-sn-glycero-3-phosphocholine/cholesterol–coated (DMPC/cholesterol-coated) CSH resulted in the robust transfer of *PE from the lipid-donor particles to plasma as compared with coating with egg lecithin/cholesterol or with either phospholipid alone. *PE efflux was also greater using DMPC/cholesterol at a 2:1 ratio as compared with 3:1 or 4:1 mole ratios, and it was more HDL specific than at a 1:1 ratio. Increasing the LC-CSH fluorescent lipid content 5-fold from our previously used concentration (23) also markedly increased the plasma *PE signal in an LC-CSH mass–dependent manner. Finally, we confirmed that saturation of lipid binding to CSH was achieved using 80 mg LC-CSH, consistent with our previous observations (23).

We also included in some of our studies fluorescence-tagged BODIPY-cholesterol (*Chol) as a component of LC-CSH to examine the solubilization of cholesterol as an alternative biomarker. Inclusion of *Chol did not affect *PE fluorescence or the HDL-SPE assay results (Supplemental Figure 2). In contrast to *PE, which was primarily found on HDL by agarose gel electrophoresis at the end of the assay (Figure 1B), *Chol transferred to all types of lipoproteins, including LDL (Figure 1B). Free cholesterol is more polar than phospholipids and is known to be able to spontaneously desorb from lipid surfaces and rapidly equilibrate between lipoproteins (25). This nonspecific cholesterol efflux exchange of *Chol from LC-CSH was linear with increasing plasma volumes but did not show saturation, unlike *PE solubilization (Figure 1C). In contrast to phospholipid efflux, *Chol efflux from LC-CSH to plasma was the highest using CSH coated with pure egg lecithin, again suggesting that different mechanisms underlie *PE and *Chol efflux to plasma (Supplemental Figure 1).

The release of *PE from LC-CSH to plasma (HDL-SPE) was temperature dependent (Supplemental Figure 2), with little or no fluorescent lipid efflux occurring at 4°C for incubation durations up to 6 hours. Kinetic studies revealed that both *PE and *Chol transfer to plasma at 37°C was linear from 5- to 60-minute incubation durations but was saturated after longer time intervals (Supplemental Figure 2). Given these findings, we typically used a 60-minute incubation for our assay. The HDL-SPE assay was also linear over a wide range of plasma volumes (10–35 μL plasma; Figure 1, B–D), and we chose 25 μL plasma as the standard sample volume.

**Figure 2:**
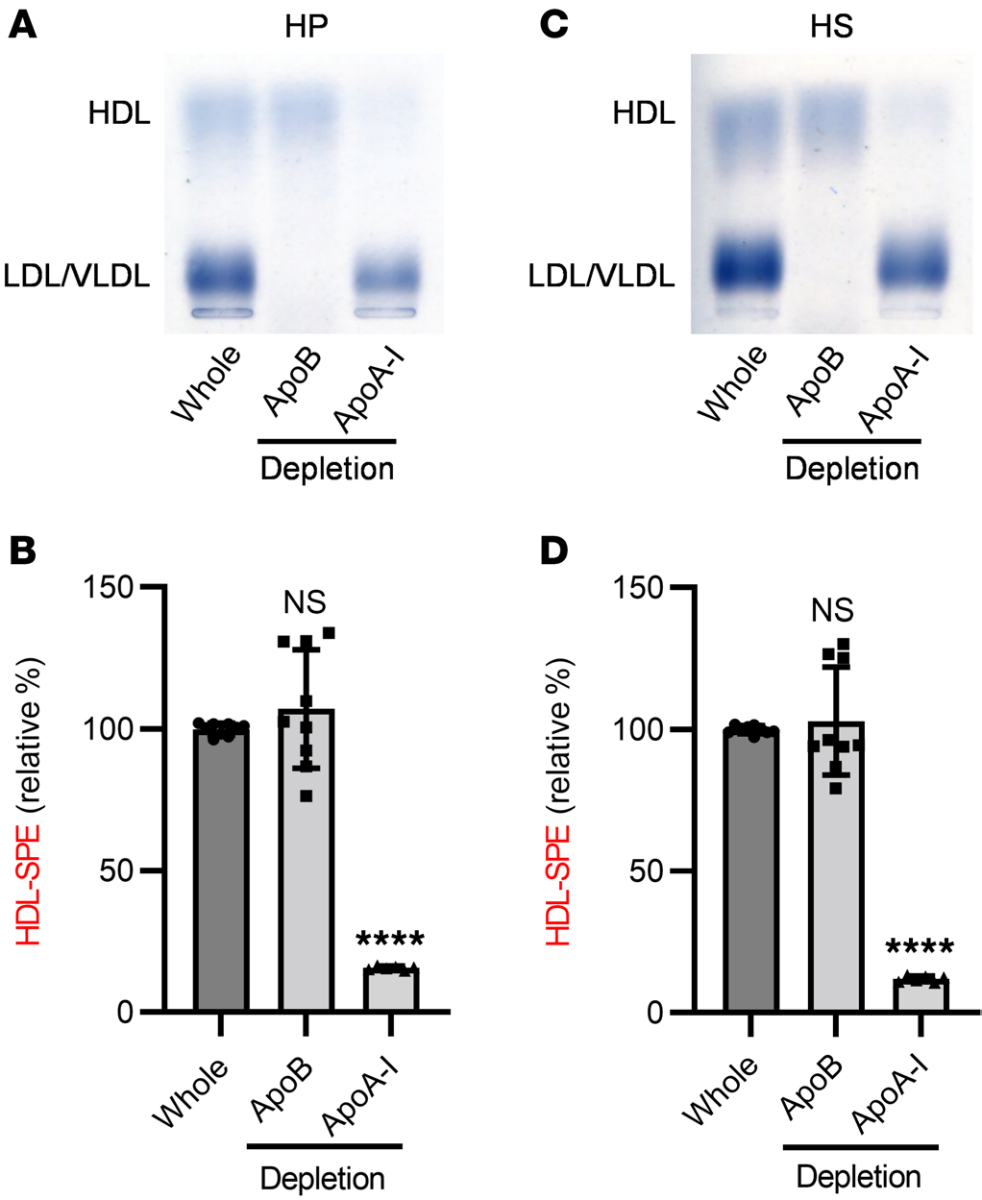
Efflux of fluorescent PE from donor LC-CSH particles to plasma HDL is apoA-I dependent. HDL or LDL/VLDL was removed from (**A** and **B**) HP or (**C** and **D**) pooled normal HS by immunodepletion with anti–apoA-I or anti-apoB antibodies, respectively. SB staining demonstrated apoA-I immunodepletion of HDL and apoB immunodepletion of LDL/VLDL from both HP and HS with anti–apoA-I and anti-apoB antibodies, respectively. (**B** and **D**) HDL-SPE was markedly reduced in apoA-I– but not apoB-depleted samples. Dunnett’s multiple-comparison test was performed using whole plasma or serum samples as controls (*n* = 9). For apoB depletion, data represent 9 samples; triplicate assays were performed in 3 independent experiments. For apoA-I depletion data represent 6 samples; triplicate assays were performed in 2 independent experiments. ****P < 0.0001 versus control.

HDL-SPE highly correlated with the amount of *PE associated with HDL in plasma at the end of the assay (Figure 1, B and E). Adding purified HDL as the sample was also much more effective in solubilizing *PE than using either isolated LDL or VLDL (Figure 1F). HDL-SPE results from human plasma (HP) and human serum (HS) samples from the same donor were highly correlated (*r* = 0.9931, *P* = 8.88 × 10^–8^; Supplemental Figure 3). Consistent with this finding, addition of heparin-sodium or EDTA-2K to serum also did not alter HDL-SPE (Supplemental Figure 3). Taken together, these results confirmed that either plasma or serum is a suitable specimen type for the assay. HDL-SPE was markedly reduced in both plasma and serum after apoA-I immunodepletion of HDL and was unaltered in apoB immune-depleted plasma and serum (Figure 2), confirming its apoA-I and HDL dependence.

The HDL-SPE assay showed excellent reproducibility and precision (Supplemental Figure 4). The interplate and intraplate coefficients of variation (CVs) were consistently below 10%. It was also highly reproducible in both HP and HS samples that underwent multiple freeze-thaw cycles (Supplemental Figure 4). The HDL-SPE assay is also relatively stable with prolonged storage of plasma or serum samples at either –20°C or –80°C (Supplemental Figure 4). The percentage of biological variability of the HDL-SPE assay for repeat plasma samples from the same individuals over a 1-year time period was 7.66% ± 5.42% (mean ± SD), which is at least comparable to the CEC assay (see Supplemental Figure 4). We also assessed the lot-to-lot variability and storage stability of the LC-CSH reagent (Supplemental Figure 5). The LC-CSH lot CV percentage was relatively good (≤10%) with no significant difference in HDL-SPE assay results between the different batches. Furthermore, we confirmed that, even if there was a 10% difference between reagent lots, the effect could be minimized by correcting with the same positive control. LC-CSH was also extremely stable following storage for 7 days at either 4°C, –20°C, or –80°C and remained stable at 4°C for at least 2 weeks and at –20°C for at least 6 weeks.

### Exchangeable HDL apolipoproteins primarily mediate HDL-SPE.

**Figure 3:**
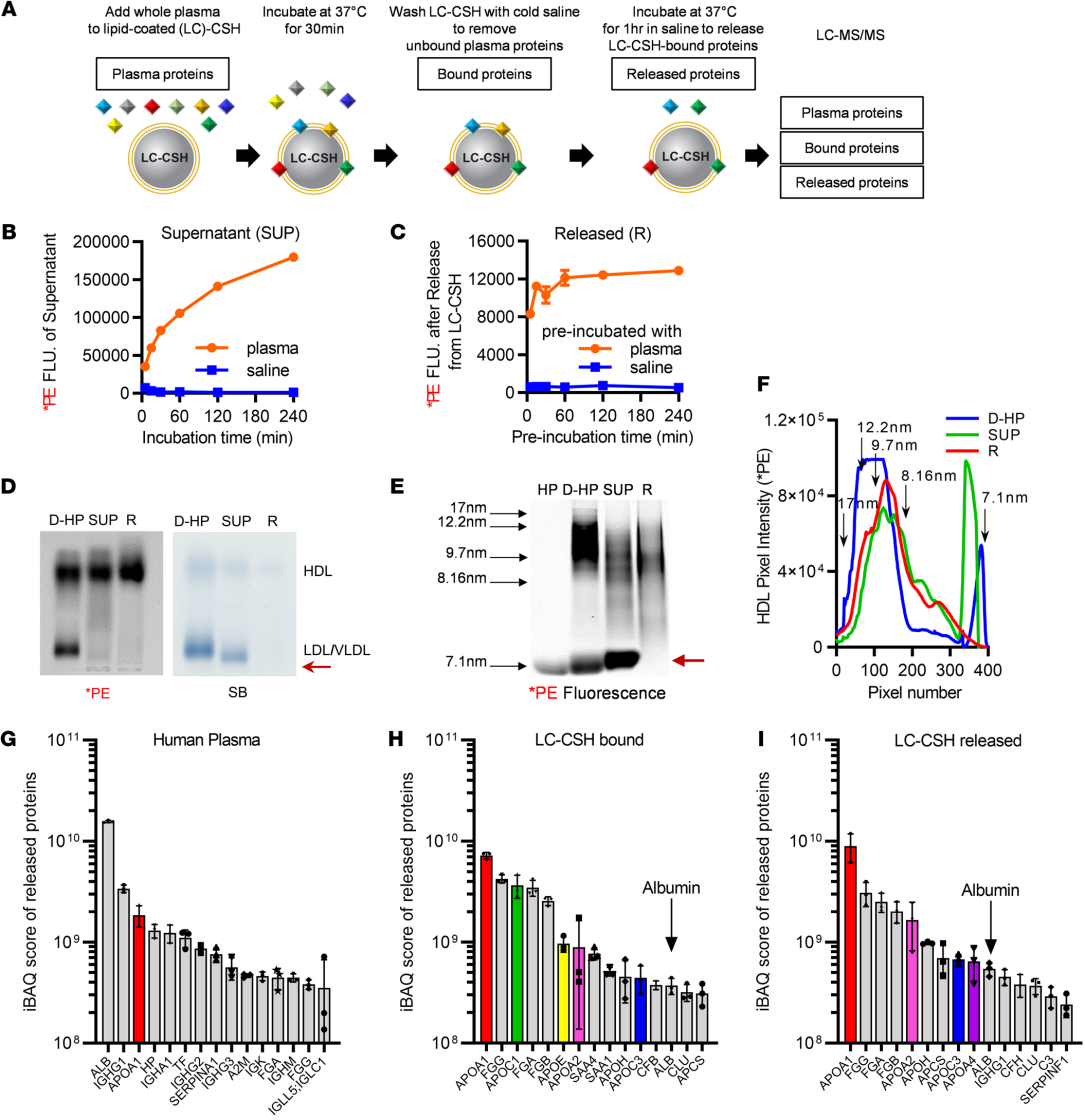
Exchangeable HDL apolipoproteins mediate HDL-SPE. (**A**) Assay to identify plasma proteins that mediate HDL-SPE. (**B**) Time course of LC-CSH *PE efflux to HP versus saline after incubation at 37°C for the indicated durations. (**C**) Release of *PE from LC-CSH in **A**, after washing and subsequent incubation with saline at 37°C for 1 hour. (**D**) Agarose gel electrophoresis: D-HP, HP directly labeled with *PE; SUP, supernatant after incubation of HP with fluorescent *PE-labeled LC-SCH for 30 minutes; R, plasma proteins released to saline from LC-CSH preincubated with HP. (**E**) Fluorescent native gel lipoprotein electrophoresis and (**F**) Kymograph analysis of *PE fluorescence revealed that the supernatant and released plasma proteins contained mostly small *PE-tagged HDL-like particles. Red arrow indicates small HDL/albumin band. HP, unlabeled HP. (**G**–**I**) iBAQ analyses revealed the relative distribution of the identified plasma proteins. (**G**) HP proteins, (**H**) LC-CSH–bound proteins, and (**I**) LC-CSH–released proteins. All data are the mean ± SD from triplicate assays.

As diagrammed in Figure 3A, we investigated the mechanism for the release of *PE during the HDL-SPE assay by characterizing the lipoprotein particles produced during the solubilization of lipids from LC-CSH and the type of plasma proteins involved. LC-CSH donor particles were first incubated with plasma for 30 minutes (within a linear time interval of the assay; Figure 3B), and supernatant was collected for analysis. LC-CSH particles previously incubated with plasma were then rapidly and extensively washed with cold saline (Supplemental Figure 6) followed by further incubation with saline at 37°C for various durations to monitor the release of additional *PE (Figure 3C), presumably from proteins still bound to the LC-CSH donor particles. During the first 30-minute initial incubation of plasma with LC-CSH, released *PE into the supernatant was found by agarose gel electrophoresis to only be associated with HDL-like particles that contained relatively little neutral core lipids as shown by Sudan black (SB) staining (Figure 3D). Similarly, released *PE into saline after incubation of washed LC-CSH (released fraction R) also appeared in HDL-like particles that had even less intense SB staining than the supernatant (Figure 3D).

To further characterize the lipoprotein particles produced during the HDL-SPE assay, we also performed 1D native gel fluorescence lipoprotein electrophoresis (Figure 3, E and F), which revealed that mostly small *PE-tagged, HDL-like particles (apparent size ≤9.7 nm) were formed. The HDL particle size distributions were similar in both the supernatant (SUP) and released (R) pools and were consistent with the heterogenous size distribution of nascent HDL particles previously reported to form by apoA-I–mediated solubilization of multilamellar vesicles of varied lipid composition (26, 27). Direct measurement of the actual size of released particles (R), negatively stained on electron micrographs (Supplemental Figure 7), confirmed that they were small HDL-like particle (median diameter = 8.8 nm) which mostly appeared to be spherical.

Notably, on native gels, there was an absence of larger-sized *PE-labeled HDL for both supernatant and released HDL when compared with directly fluorescent lipid–labeled whole HP (D-HP). The Released HDL particles also had very little SB staining, consistent with the lack of SB staining of HDL in agarose gel electrophoresis (Figure 3D), confirming that they contained little, if any, core hydrophobic lipids. Although supernatant and released HDL particles had similar amounts of *PE labeling, the *Chol content of released HDL was markedly lower than that of supernatant HDL, consistent with diffusion-mediated transfer of *Chol to HDL in the supernatant fraction (Supplemental Figure 7).

Finally, we observed *PE in a small HDL/albumin migrating band (28, 29) in the supernatant fraction (Figure 3E, red arrow). This finding suggests a potential role for albumin in the HDL-SPE assay. Albumin alone, however, only supported a negligible amount of *PE efflux from LC-CSH, but when added to HDL, HDL-SPE was increased, suggesting that albumin may facilitate HDL-SPE by possibly serving as a sink for HDL *PE (Supplemental Figure 8). Neither lecithin-cholesterol acyltransferase (LCAT)nor phospholipid transfer protein (PLTP) activity correlated with HDL-SPE, and LCAT inhibition only slightly decreased HDL-SPE (~7%), indicating that LCAT and PLTP played minor roles in the HDL-SPE assay (Supplemental Figure 8).

We used intensity-based absolute quantification (iBAQ) analysis to determine the relative distribution of identified plasma proteins in whole plasma, LC-CSH–bound proteins, and proteins released from LC-CSH (Figure 3, G–I). Proteomics analysis confirmed that mostly apoA-I, but also to a lesser degree other exchangeable HDL-associated proteins (apoA-II, apoC-III, and apoA-IV), bound to LC-CSH when first incubated with plasma and were then later released from LC-CSH when incubated with saline (Figure 3, G–I, and Table 1). The complete list of identified plasma proteins, LC-CSH–bound plasma proteins, and LC-CSH–released plasma proteins is shown in Supplemental Tables 1–3, respectively. The plasma proteins that do not bind to LC-CSH are listed in Supplemental Table 4. Interestingly, although it was relatively low compared with apoA-I in absolute amounts, apoA-IV was highly enriched in the LC-CSH–released pool (Figure 3I and Table 1). This finding is consistent with previous reports showing that plasma apoA-IV is easily displaced from HDL, enhances CEC (30), and, moreover, is inversely associated with CAD (31). Since apoA-IV distributes between HDL particles (30%–50%) and a lipid-free state (32), non-HDL-associated apoA-IV may also mediate HDL-SPE (20, 33). In contrast, apoE, which is one of the larger-sized exchangeable proteins and firmly binds lipoproteins (34), was also enriched in the proteins bound to LC-CSH (Figure 3H and Table 1) but was not detected among the proteins that were later released from LC-CSH (Figure 3I and Table 1). Thus, lipid- or lipoprotein-binding affinity may not have been the only factor driving the proportion of bound versus released apolipoproteins in our assay.

Albumin, the most abundant plasma protein (Figure 3G), was nearly 20-fold less abundant than apoA-I in both the bound and released proteins (Figure 3, H and I, and Table 1). In addition, very little apoB bound to LC-CSH, and almost none was found in the released fraction, consistent with the lack of *PE labeling of LDL (Table 1) and the known nonexchangeability of this apolipoprotein. Fibrinogen bound to LC-CSH (Figure 3H) and was later released (Figure 3I), but when used alone as a potential acceptor or when added to human serum (HS), fibrinogen did not substantially further increase HDL-SPE (Supplemental Figure 8).

**Figure 4:**
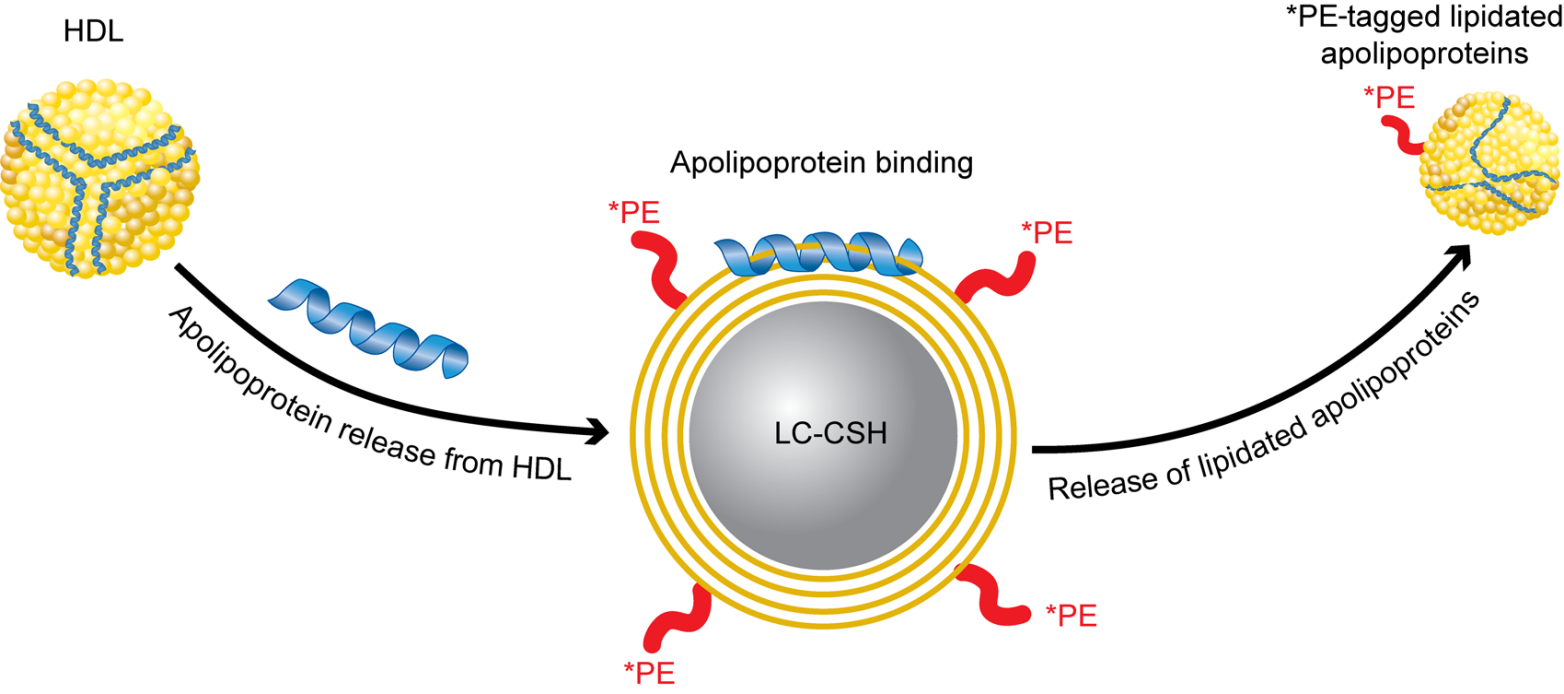
Model of apoA-I/HDL–mediated *PE efflux from LC-CSH particles. ApoA-I and other HDL-associated exchangeable apolipoproteins dissociate from HDL, bind to LC-CSH, solubilize *PE and other lipids, and are then released as small *PE-tagged lipidated apolipoprotein particles.

Despite the binding of many different types of plasma proteins to LC-CSH to varying extents, both the proteomic iBAQ (Figure 3H and Supplemental Table 2) and the SDS-PAGE gel (Supplemental Figure 6) analyses showed that HDL-derived exchangeable apolipoproteins (Table 1), particularly apoA-I, appeared to account for the vast majority of plasma-mediated *PE efflux from LC-CSH (Figure 2). The model shown in Figure 4 summarizes our findings concerning the mechanism for the apolipoprotein-mediated solubilization of lipids from LC-CSH donor particles, accounting for the signal generated by the HDL-SPE assay.

### HDL-SPE is significantly associated with CAD in case-control studies independent of HDL-C and apoA-I.

**Figure 5:**
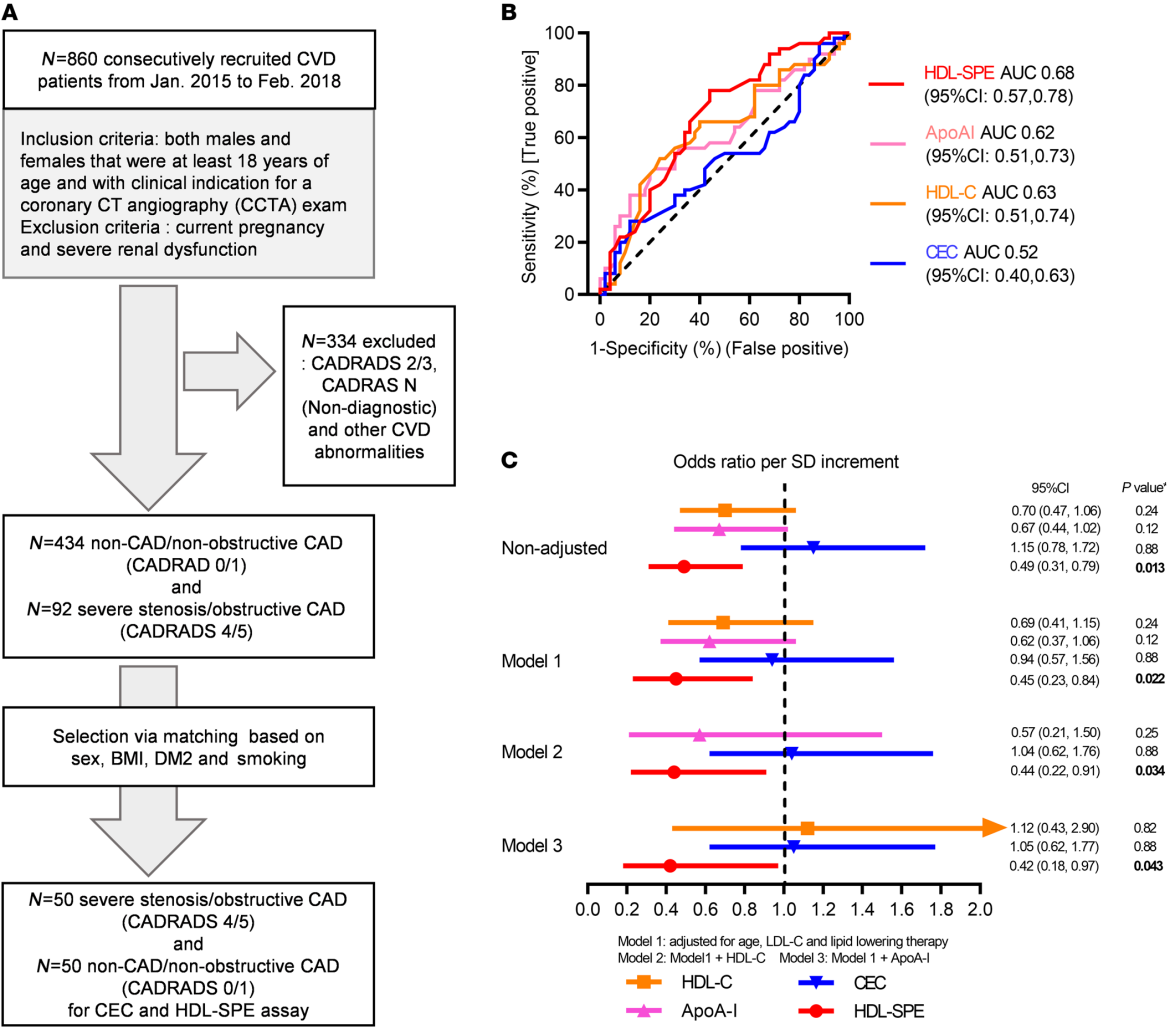
Clinical Study I: HDL-SPE, but not CEC, HDL-C, or apoA-I, associates with severe CAD in a CVD cohort, independent of traditional risk factors. (**A**) Recruitment scheme for participants in Clinical Study I. (**B**) ROC curves for HDL-SPE, CEC, HDL-C, and apoA-I. (**C**) ORs are reported per 1 SD increment for HDL-SPE, CEC, HDL-C, and apoA-I based on univariate (nonadjusted) and multivariate regression analyses adjusted for risk factors and biomarkers as indicated. *P* values in **C** were calculated after adjusting the FDR, and the FDR-adjusted *P* value of less than 0.05 was considered statistically significant.

We conducted 2 pilot case-control clinical studies to initially assess the performance of our HDL-SPE assay as a potential biomarker for CAD. In the first study, we analyzed a total of 100 participants with either severe CAD (*n* = 50) or no CAD (non-CAD) (*n* = 50) (Figure 5). Individuals with severe CAD were defined as having severe stenosis or obstructive disease based on coronary CT angiography (CADRADS 4/5), and non-CAD/nonobstructive CAD participants were defined as having no stenosis or minimum stenosis (CADRADS 0/1). As shown in Figure 5A and Table 2, the frequency matching criteria were sex, BMI, type 2 diabetes mellitus (DM), and smoking. The case-control groups had no significant difference in HDL-related marker levels, such as HDL-C, HDL particle number (HDL-P), HDL size (HDL-Z), or plasma apoA-I levels (Table 2). HDL-SPE, but not CEC, was significantly decreased in the group with severe CAD (CEC: 1.04 ± 0.19 vs. 1.01 ± 0.18, *P* = 0.49; HDL-SPE: 0.93 ± 0.14 vs. 1.04 ± 0.18, *P* = 0.002; severe CAD vs. non-CAD; Table 2). In the entire cohort, HDL-SPE weakly correlated with CEC but was modestly correlated with HDL-C and HDL-P and even more so with plasma apoA-I (Supplemental Figure 9). Receiver operating characteristic (ROC) curve analysis indicated that HDL-SPE was a better univariate biomarker than HDL-C, plasma apoA-I, or CEC for identifying individuals with severe CAD (Figure 5B). Multivariate logistic regression analyses adjusted for traditional risk factors revealed that HDL-SPE was still significantly associated with severe CAD, but the same was not true for HDL-C, apoA-I, or CEC (Figure 5C).

We also compared the ability of the HDL-SPE and CEC assays to predict coronary artery plaque burden in 208 arteries from a subcohort of 73 participants with known CAD from this study (Supplemental Figure 10). Multivariate regression analysis revealed that after adjustment, HDL-SPE remained significantly inversely correlated with total plaque burden (*P* = 0.004), noncalcified plaque burden (*P* = 0.006), and the amount of fibro-fatty plaques (*P* = 0.036), whereas CEC was not associated with any measure of plaque burden based on coronary computed tomography angiography (CCTA) analysis.

**Figure 6:**
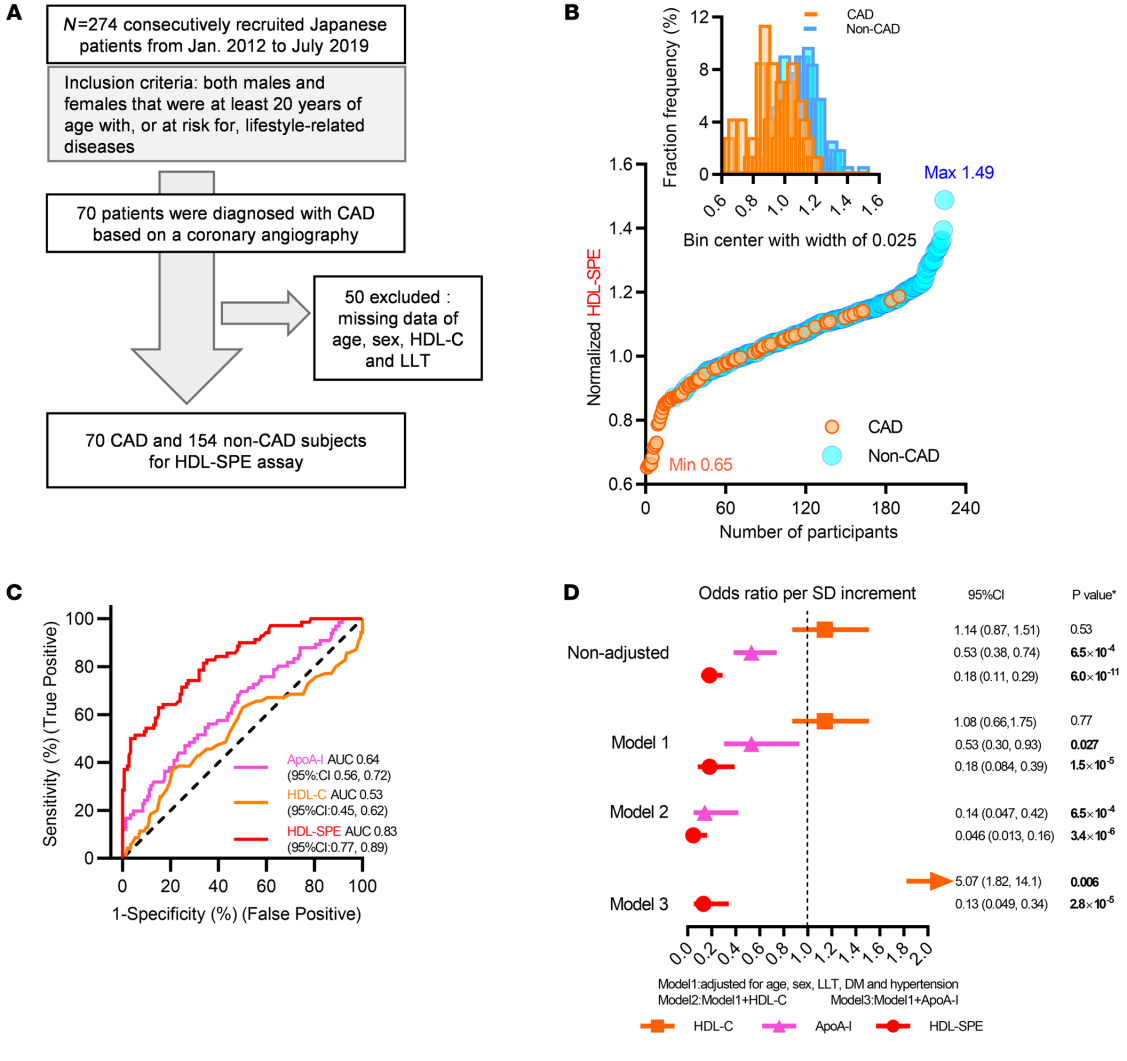
Clinical Study II: HDL-SPE highly and apoA-I significantly associate with CAD in a Japanese cohort. (**A**) Recruitment scheme for participants in Clinical Study II. (**B**) Ranking and frequency distribution of HDL-SPE values among CAD and non-CAD participants. Max, maximum; Min, minimum. (**C**) ROC curves for HDL-SPE, apoA-I, and HDL-C. (**D**) ORs are reported per 1 SD increment for HDL-SPE and apoA-I and HDL-C levels based on univariate (nonadjusted) as well as multivariate logistic regression analyses adjusted for risk factors and biomarkers as indicated. The *P* value in **D** was obtained after adjusting the FDR, and the FDR-adjusted *P* value of less than 0.05 was considered statistically significant.

A Japanese cohort (*n* = 224) in Clinical Study II (Figure 6A and Table 3) included both CAD (*n* = 70) and non-CAD participants (*n* = 154). CAD was evaluated on the basis of standard coronary angiography (Figure 6A). CAD and non-CAD participants had significant differences in age, sex, hypertension, lipid-lowering therapy (LLT), DM, and apoA-I levels (Table 3). HDL-SPE was also significantly reduced in participants with CAD (Table 3). HDL-SPE values were normally distributed and ranged from 0.65 to 1.49 relative units (Figure 6B). Both HDL-C and apoA-I strongly correlated with HDL-SPE (Supplemental Figure 11). ROC curve analysis demonstrated an optimum cut-point of 1.04 for HDL-SPE to discriminate between CAD and non-CAD, with an AUC of 0.83, a sensitivity of 74.3%, and a specificity of 72.7% (Figure 6C). In contrast, HDL-C and apoA-I had much lower AUCs of only 0.53 and 0.64, respectively. The inverse association of HDL-SPE and apoA-I with CAD remained highly statistically significant after adjusting for age, sex, LDL-C, LLT, DM, and hypertension (model 1; Figure 6D). After additional adjustments for HDL-C, both HDL-SPE and apoA-I remained significant and inversely associated with CAD. Finally, after adjusting for apoA-I, in addition to the model 1 parameters, HDL-SPE still remained significant.

We also examined the results of the HDL-SPE assay in patients with very high levels of HDL-C, given the recent reports of increased CVD risk with high HDL-C (35). Analysis of HDL-SPE in a subcohort of participants with HDL-C levels of 80 mg/dL or higher (*n* = 16) showed that apoA-I, but not HDL-C, correlated linearly with HDL-SPE (Supplemental Figure 12). These findings suggest that the HDL-SPE assay may still correlate with HDL function and still be predictive of CAD even in patients with very high HDL-C levels, but this will require additional studies with a much larger sample sizes to confirm.

### HDL-SPE in a general population cohort is significantly associated with the future development of CVD events, independent of HDL-C and apoA-I.

**Figure 7:**
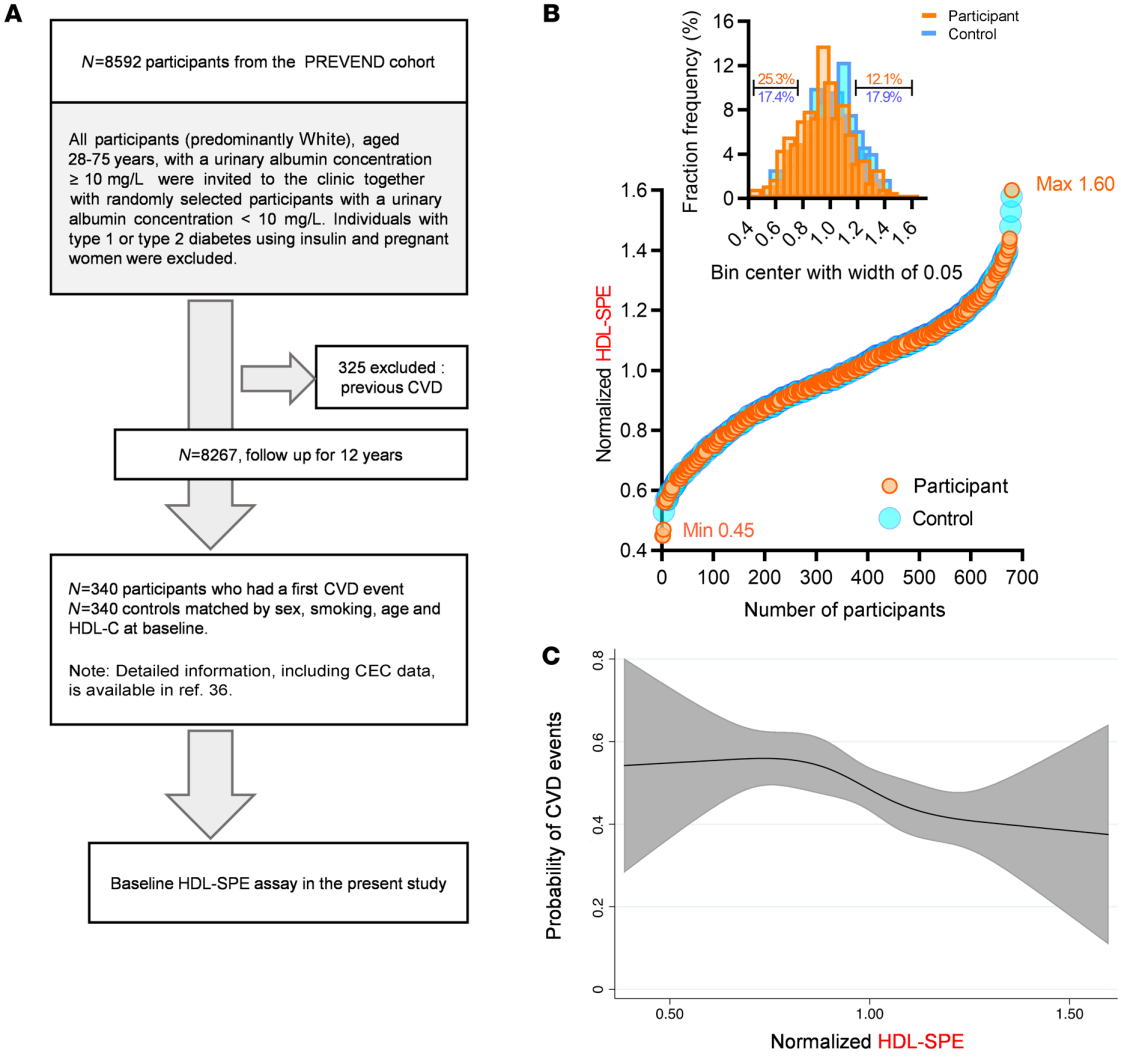
Clinical Study III: HDL-SPE is prospectively associated with CVD events independent of traditional risk factors. (**A**) A prospective study among participants of the PREVEND cohort (36) was used to evaluate the prospective association of HDL-SPE with CVD events in the general population. (**B**) Ranking and frequency distribution of HDL-SPE values among participants who subsequently did (Particpant) or did not (Control) develop CVD events during follow-up. (**C**) Probabilities of CVD events associated with normalized HDL-SPE values were obtained by multivariate conditional logistic regression using restricted cubic splines with 4 knots, adjusted for BMI, DM, LDL-C and TG levels, hypertension, and hsCRP.

We also measured HDL-SPE in a nested case-control study constructed from the male and female participants of the Prevention of Renal and Vascular End-Stage Disease (PREVEND) study (Figure 7A), a large and well-characterized general population cohort from the Netherlands (36). In this longitudinal study, participants were individually matched to controls for age, sex, current smoking behavior, and HDL-C levels. As shown in Table 4, subsequent participants had at baseline, as expected, a significantly higher prevalence of cardiovascular risk factors, whereas, because of the matching, HDL-C and apoA-I levels were very similar between the groups. In univariate, as well as age-, sex- and HDL-C–adjusted analyses (Table 5), CEC was consistently positively associated with HDL-SPE. As previously described for CEC (36), the results for HDL-SPE were also strongly positively related to HDL-C and apoA-I levels (Table 5). Consistent with our previously described in vitro studies (Supplemental Figure 8), the HDL-SPE assay in this cohort did not correlate with either LCAT (*r* = 0.08, *P* = 0.48) or PLTP (*r* = 0.13, *P* = 0.24) levels in Pearson’s correlation analyses. The frequency distribution of HDL-SPE is shown in Figure 7B. In a univariate conditional logistic regression analysis (Table 6), HDL-SPE showed an inverse association with incident CVD events (OR per 1 SD increase, 0.14; 95% CI, 0.05–0.40; *P* < 0.001). After adjusting for BMI, alcohol intake, diabetes status, hypertension, and use of lipid-lowering drugs, this association remained essentially unchanged (model 1: OR per 1 SD increase, 0.14; 95% CI, 0.05–0.42; *P* < 0.001). After further adjustment for total cholesterol, apoA-I, and triglycerides (TGs) (model 2: OR per 1 SD increase, 0.16; 95% CI, 0.05–0.50; *P* = 0.002) and also for high-sensitivity C-reactive protein (hsCRP), urinary albumin excretion (UAE), and estimated glomerular filtration rate (eGFR) (model 3, OR per 1 SD increase = 0.09; 95% CI, 0.03–0.33; *P* < 0.001), the inverse association of HDL-SPE with incident CVD remained strongly significant. Notably, the inverse association of HDL-SPE with incident CVD events did not change after additional adjustment for CEC (model 4: OR per 1 SD increase, 0.09; 95% CI, 0.03–0.33; *P* < 0.001). Restricted cubic spline analysis showed that the probability of an ASCVD event in the general population was inversely related in the range of approximately 0.8 to 1.2 normalized HDL-SPE units after adjustment for traditional CVD risk factors (Figure 7C). The CEC assay also showed a strong inverse association with incident CVD events (Figure 6B). The OR values, however, were lower for HDL-SPE compared with CEC after univariable analysis, as well as after the adjustments in models 1, 2, and 3 (0.14 vs. 0.27, 0.14 vs. 0.28, 0.16 vs. 0.28, and 0.09 vs. 0.24, respectively, for HDL-SPE vs. CEC). Furthermore, the point estimates of the OR for CEC were weaker after an additional adjustment for HDL-SPE (model 4A: OR per 1 SD increase, 0.37; 95% CI, 0.17–0.81; *P* = 0.013 vs. model 3A: OR per 1 SD increase, 0.24; 95% CI, 0.11–0.51; *P* = 0.00042). Conversely, the point estimates of the OR for HDL-SPE remained essentially unchanged after additional adjustment for CEC (model 4A: OR per 1 SD increase, 0.09; 95% CI, 0.03–0.33; *P* = 0.00058 vs. model 3A: OR per 1 SD increase, 0.09; 95% CI, 0.03–0.33; *P* = 0.00084).

## Discussion

We developed a cell-free, HDL-specific phospholipid efflux assay (HDL-SPE) for the assessment of CAD risk on the basis of HDL functionality in whole plasma or serum. One of the main advantages of the HDL-SPE assay is that it can be readily automated, unlike the various CEC assays currently in use. Analyses of HDL-SPE on CVD outcomes in our 3 clinical cohorts provide strong evidence that it is a better marker than current conventional measures of HDL, such as HDL-C and apoA-I. Pilot clinical case-control studies I and II showed that HDL-SPE was also a strong inverse predictor of CAD, independent of apoA-I concentration and HDL-C, in both the American and Japanese cohorts. We also found that HDL-SPE was strongly inversely associated with incident CAD events in a large nested case-control cohort from the prospective PREVEND study, independent of HDL-C and apoA-I plasma levels (36).

We have previously shown that *PE, used in our HDL-SPE assay, is a nonexchangeable phospholipid, which, unlike *Chol, does not spontaneously transfer from labeled to unlabeled lipoproteins in whole plasma because of its greater hydrophobicity (23). ApoA-I is known to solubilize lipids from multilamellar vesicles (MLVs) forming pre–β-HDL–like particles containing *PE, *Chol, and apoA-I (23). Furthermore, MLV-derived *PE specifically incorporates into HP HDL in vitro (23) and into mouse plasma HDL after intravenous injection in vivo (24). In the present study, we found that HDL in plasma specifically solubilized *PE from artificial donor particles (LC-CSH) and was predictive of CAD.

The main mechanism by which HDL-SPE occurs appears to be dependent on apoA-I and other exchangeable apolipoproteins. The ability of apolipoproteins to dissociate from HDL has previously been shown to be associated with atherosclerosis and CAD (15–17). Our results are consistent with the following steps for the HDL-SPE assay: (a) apolipoprotein dissociation from plasma HDL, (b) apolipoprotein binding to LC-CSH and, (c) solubilization of LC-CSH lipids and their subsequent release as small *PE-labeled apolipoprotein particles (Figure 4). It is likely that the hydrophobic face of exchangeable HDL apolipoproteins can only become available for binding to LC-CSH after its dissociation from HDL (37, 38). We cannot exclude, however, the possibility that some holoparticle HDL binding/fusion to the LC-CSH donor particles may also be occurring and contributing to the release of *PE.

The donor particles used in our assay may potentially mimic the ABCA1-modified cell membrane lipid domains on cells, by permitting apoA-I to insert and then later solubilize and remove phospholipids. The particular ratio of cholesterol to phospholipid used in our assay may create lipid-packing defects in the donor particles that enable apolipoprotein binding and subsequent lipid solubilization (25, 39). CSH crystals also have regions of high curvature (Supplemental Figure 2), which may also contribute to defects in lipid packing. The mimicking of ABCA1-dependent cellular cholesterol efflux could, therefore, potentially explain the association of the HDL-SPE assay with clinical CAD events. It is important to note, however, that a significant fraction of cholesterol and other lipids is in the extracellular space of the complex types of plaques that rupture and cause clinical events (40, 41). Extracellular cholesterol is well known to form crystals in complex plaques, but it also exists in aggregated lipoproteins and other types of complexes with phospholipids (42, 43). Only in early atherosclerotic lesions is most cholesterol located inside cells like macrophages (44, 45). Our HDL-SPE assay, a better predictor of CAD events than HDL-C and CEC, may therefore also assess the ability of HDL to solubilize and remove excess extracellular lipid. This is consistent with our finding that HDL-SPE showed an inverse correlation with noncalcified plaque burden, particularly fibro-fatty lesions (Supplemental Figure 10 and Supplemental Table 5). Additional studies will be needed to better understand the strong association of HDL-SPE with CAD.

A comparison of our HDL-SPE assay with related HDL functionality assays (see Supplemental Table 6) revealed that the our assay offers several technological advances. For example, Oda et al. (15, 16) showed in a small study an inverse association of HDL/apoA-I exchangeability by electron spin resonance (ESR), but ESR is not a technology or an instrument routinely available to clinical laboratories. This method may also not provide complete information of HDL functionality, given its dependence on the exchange of only a single apolipoprotein, namely apoA-I. Similarly, the recently described cell-free HDL function assay based on the apoA-I exchange rate (AER) only monitors exogenous fluorescence-tagged apoA-I (17). Most studies to date that have assessed CAD risk by HDL functionality still use the CEC assay and are based on the use of radioisotopes (^3^H-cholesterol) and cultured cells, which is very labor intensive and impractical to do in a clinical laboratory. Another limitation of the CEC assay is its considerable batch-to-batch variability even within the same laboratory, which hinders the necessary standardization needed for routine diagnostic testing (46, 47). Finally, the CEC assay and some of the other recently developed cell-free assays (13, 14) also require a complicated plasma-processing step to prepare apoB-depleted plasma, which, depending on how it is done, may alter the true CEC of HDL (48).

It is important to note that our Clinical Studies I and II used to evaluate the HDL-SPE assay have a limited number of participants and that some of the multivariate adjustments could be overfitted. The nested case-control design of our PREVEND study (36) does have the advantage of matching for variables that are closely related to CEC (HDL-C, apoA-I, and sex), but it is important to note that this could lead to an overestimation for the strength of the association of the HDL-SPE assay with CVD. In the PREVEND study, the interval in which the HDL-SPE assay is linear is approximately in the range of 0.8 to 1.2 normalized HDL-SPE units. It may be linear outside this range, but because of the smaller number of participants at the extreme values at both ends, the CI is too wide to make reliable predictions. Approximately 20% of the study population had an HDL-SPE index below 0.8 and, based on our other analyses, appear to be at increased risk. For many other biomarkers, including cardiovascular biomarkers, the 75th or 80th percentile is frequently used as a dichotomous variable to separate high-risk from lower-risk individuals. These results suggest that this cutoff could possibly be suitable for our assay as well, but additional studies will be needed to determine its optimum cut-point for clinical use. Another potential limitation of the nested case-control design in the PREVEND study is that statistical analyses relating to risk prediction could not be formally carried out, since, per design, the value of what was referred to as risk was preset. Because of these limitations, it will be important to conduct additional validation studies of the HDL-SPE assay in other populations and to assess its clinical utility.

Finally, we also did not fully explore all the different potential assay parameters, such as the lipid composition of donor particles, which could affect our assay signal and its association with CAD. We chose to use trace amounts of a nontransferable fluorescent PE in donor particles to monitor apolipoprotein-mediated phospholipid efflux to plasma HDL as a measure of HDL functionality. PE has been shown to be among the phospholipids specifically removed by apolipoproteins from cells and membranes (49). Interestingly, there is some evidence that HDL PE content may potentially alter HDL metabolism and CAD risk (50, 51). Thus, it may be of interest in future studies to explore potential modulation of our HDL-SPE assay based on HDL PE content. Indeed, we expect that our HDL-SPE assay and its possible new iterations will provide a convenient platform for future research studies on HDL function and composition and how they relate to CVD.

In summary, we have established the HDL-SPE assay for assessment of the functional ability of HDL to efflux phospholipids. Our combined data consistently show that our relatively simple HDL-SPE assay captures a pathophysiologically relevant parameter of HDL function that is at least equivalent to the CEC assay in its association with prevalent and incident CAD. Larger studies with different study designs and different patient groups of even more diverse ethnicities will still be needed to firmly establish the strength of the association of our HDL-SPE assay with ASCVD detection. Our ongoing efforts to implement the HDL-SPE assay on an automated high-throughput analyzer will facilitate its future evaluation for CAD risk assessment in a routine clinical laboratory setting.

## Methods

### LC-CSH preparation.

LC-CSH crystals were prepared as previously described with minor modifications (23). Briefly, lipid removal agent (LRA) (SUPELCO, catalog 13358-U) was used as a source of CSH particles. LC-CSH crystals were formed by combining 11.8 mg (17.7 μmoles) DMPC (Avanti Polar Lipids, catalog 850345C), together with 3.39 mg (8.8 μmoles) cholesterol (MilliporeSigma, catalog C8667), 305 μg (200 nmoles) nonexchangeable head-group–labeled, fluorescence-tagged phosphatidylethanolamine [1,2-dioleoyl-sn-glycero-3-phosphoethanolamine–*N*-(lissamine rhodamine B sulfonyl)] (Avanti Polar Lipidsm, catalog 810150C), and/or 55.0 μg (100 nmoles) fluorescent Bodipy cholesterol [23-(dipyrrometheneboron difluoride)-24-norcholesterol] (Avanti Polar Lipids, catalog 810255) from their respective stock solutions in chloroform. The LC-CSH lipid mole ratio is as follows: DMPC/cholesterol/*PE/*Chol at 2:1:0.02:0.01. The lipid mixtures were dried under nitrogen. To form LC-CSH, 80 mg LRA along with 2 mL saline was added to the dried lipid mixture and then vortexed for 10 minutes. This lipid/LC-CSH ratio provides sufficient lipid to completely cover the surface of LRA particles, thereby preventing direct lipoprotein binding to non-lipid-coated LRA surfaces (23). The resulting LC-CSH crystals were pelleted by centrifugation (2,000 rpm, 2 min), and the supernatant was removed and replaced with 5 mL saline. This washing process was repeated 5 times to ensure removal of any potential lipid vesicles not attached to the LRA. After the final wash, the LC-CSH solution volume was brought up to 2.5 mL in saline. Confocal and transmission electron microscopy revealed the plate and needle crystal structure of the LC-CSH particles (Supplemental Figure 2).

### Plasma and serum samples.

Refer to the Supplemental Methods for details on plasma and serum samples.

### HDL-SPE and nonspecific CEC assays.

The standard assay reaction mixture (150 μL total volume) of 25 μL HP or HS (or 25 μL saline for negative controls), together with 50 μL LC-CSH and 75 μL saline were incubated in 96-well plates for 1 hour at 37°C with shaking (1,200 rpm). A sufficient volume of saline was added to the plasma and LC-CSH reaction mixture to obtain a total volume of 150 μL. After incubation, donor particles were pelleted via centrifugation (2,000 rpm for 2 min). A total of 50 μL of the supernatant was transferred to black 96-well plates along with 50 μL saline and 100 μL 1% Triton X-100 (Thermo Fisher Scientific, catalog 28314) in water. After mixing well, *PE for HDL-SPE and/or *Chol for nonspecific CEC (NS-CEC) were measured by fluorimetry (*PE: 540 nm/600 nm excitation/emission [Ex/Em]; *Chol: 485 nm/520 nm Ex/Em). All assays were performed in triplicate for analytical validation studies or in duplicate for clinical validation studies.

### Percentage of efflux calculation for HDL-SPE (*PE) and NS-CEC (*Chol).

The percentage of *PE or *Chol efflux values was given by: (supernatant sample fluorescence units [FLU] – supernatant saline FLU)/(total LC-CSH FLU) × 100. Total LC-CSH FLU was calculated using standard curves (Supplemental Figure 2). Normalized efflux values were defined as the clinical sample efflux value (FLU) divided by the reference control efflux value (FLU). Identical pooled HP from healthy donors with a normal lipid profile was used as a reference control. Normalization of efflux values in clinical studies was performed to correct for daily variation in reagent preparation and experimentation.

### HDL-SPE assay using isolated lipoproteins (HDL, LDL, and VLDL).

Isolated HDL phospholipid concentration was adjusted to 80 mg/dL with saline, the previously reported HDL phospholipid concentration in HP (52). In order to compare the specificity of HDL-SPE between lipoproteins, isolated LDL and VLDL were also used at the same phospholipid concentration as HDL phospholipid, specifically, 10 μL (8 μg), 20 μL (16 μg), or 40 μL (32 μg).

### HDL depletion or LDL/VLDL depletion.

HDL or LDL/VLDL was removed from HP and pooled normal HS by immunodepletion with anti–apoA-I or anti-apoB antibodies, respectively. For apoA-I depletion, 100 μL HP or HS was incubated with Goat Anti-Human Apolipoprotein AI Sepharose 4B Gel 11A-G1 Resin 1 mL (Academy Bio-Medical, catalog 11A-G1 Resin 1 mL) which was suspended in 400 μL saline and then collected by gravity flow using Poly-Prep Chromatography Columns (Bio-Rad, catalog 7311550) according to the manufacturer’s instructions. ApoB depletion was performed using LipoSep IP (catalog LS-01, Sun Diagnostics) according to the manufacturer’s instructions.

### CEC assay.

See the Supplemental Material for details on the CEC assay.

### Gel electrophoresis.

See the Supplemental Methods for details on gel electrophoresis.

### Proteomics.

See the Supplemental Methods for details on proteomics analyses.

### Clinical studies.

See the Supplemental Methods for details on clinical studies.

### Statistics.

Skewness and kurtosis measurements were used to assess normality. Data are presented as the mean ± SD or the median (IQR) for parametric and nonparametric variables, respectively, and as *n* (%) for categorical variables. *P* values were derived from a single unpaired, 2-tailed *t* test for parametric variables and a Mann-Whitney *U* test for nonparametric variables. Fisher’s exact test was used to determine categorical variables. The relationship between 2 variables was evaluated with a scatterplot using Pearson’s and/or Spearman’s coefficient with a 95% CI and *P* values. In Clinical Study I, the controls were selected via matching on the basis of sex, BMI, type 2 DM, and smoking, as described in the clinical studies methods section in Supplemental Methods. ROC curve analyses were conducted to evaluate the contribution of CEC or HDL-C or apoA-I and HDL-SPE to the discriminatory power of patients with CAD. Data are presented as the AUC with a 95% CI. The optimal cutoff point providing the best pair of sensitivity and specificity was calculated by the lowest distance to the top-left corner of the ROC curve. Bivariate or multivariate logistic regression analyses were used to assess crude ORs or adjusted ORs of CAD for the SD increment in each CEC, HDL-C, apoA-I, or HDL-SPE value. Traditional risk factors were included for adjustment as described in the clinical studies methods section in Supplemental Methods. In all analyses, a *P* value of less than 0.05 was considered statistically significant. Statistical analysis was performed using Stata/IC 12.0 (StataCorp) and EZR version 1.40 (Jichi Medical University, Tochigi, Japan). EZR is an improved version of the R commander utility 42 and included in the Softpedia database with a “100% CLEAN” software award (https://www.softpedia.com/get/Science-CAD/EZR.shtml). To optimize *P* values in multiple testing, the FDR-adjusted *P* values were manually calculated on the basis of the original work (53).

For the PREVEND study, differences in baseline characteristics were tested between participants who had experienced a cardiovascular event during follow-up (patients) and those who had not (controls). Categorical variables are expressed as total numbers (percentage), and differences between groups were tested with a χ^2^ test. Normally distributed continuous variables are expressed as the mean ± SD, and differences were tested using *t* tests. Skewed continuous variables are presented as the median (25th quartile, 75th quartile), and differences were assessed using a Wilcoxon rank-sum test.

Pearson’s rank correlation coefficients were used to assess relationships between baseline characteristics and HDL-SPE in crude analyses. Partial correlation coefficients were adjusted for age, sex, and HDL cholesterol. Given the nested case-control design of the study, (multivariable) conditional logistic regression analysis was used to assess the association of HDL-SPE with CVD outcome, with results being expressed as ORs with 95% CIs. Multivariable models were adjusted for established CVD risk factors. Because within the setting of the PREVEND study we investigated individually matched case-control data, conditional logistic regression analysis was used, which is more appropriate than unconditional logistic regression analysis for such data (54–56). Note that the OR per 1 SD values for CEC differ from those in our previous publication (36), where the OR per 1 SD was calculated using unconditional logistic regression analysis.

To assess the functional relationship of phospholipid efflux with the probability of CVD events, we used restricted cubic spline analysis with 4 knots placed at recommended percentiles according to Harrell. A logistic regression with the spline term was performed, with adjustment for BMI, DM, LDL cholesterol, TG levels, hypertension, and hsCRP. Two-sided *P* values of less than 0.05 were considered statistically significant. Statistical analysis was performed using STATA version 15.0 (StataCorp). The OR for the association of HDL-SPE with CVD in the PREVEND cohort is based on the continuous variable of HDL-SPE. Cubic splines analysis is based on categorized data, therefore, it is possible that there is an underestimation of the effect size. Furthermore, as the output created by a logistic regression depicts proportions and the output of a cubic splines analysis depicts a probability, a direct comparison of the 2 analyses is not formally possible, and a variation in the results achieved by these 2 types of analysis can be expected. ORs are not absolute, but depend on the reference, which in these studies is per SD.

### Study approval.

The protocol for Clinical Study I participants at the NIH Clinical Center (ClinicalTrials.gov NCT01621594) was approved by the IRB of the NHLBI, and all participants provided informed consent at enrollment. The Clinical Study II protocols (nos. C17-R007, 122, 142, and 158) were approved by the ethics committee of Jichi Medical University, and all participants provided informed consent at enrollment. The PREVEND study was approved by the medical ethics committee of the University Medical Center Groningen (approval no. MEC96/01/022), and all participants provided written informed consent.

### Data availability.

Values for all data points in the figures can be found in the supplemental Supporting Data Values file.

## Author contributions

MS, EBN, and ATR conceptualized the project. methodology, MS, EBN and ATR designed the methodology. MS, EBN, and MPP performed study validation. MS, AVS, AMA, SMG, JLCA, and UJFT conducted formal analyses. MS, EBN, MPP, YL, AVS, AMA, LAF, SMG, AKD, KJ, MH, JT, and UJFT conducted experiments. resources, MH, MLS, RDS, RPFD, MYC, KK, and NNM provided resources. EBN and MS wrote the original draft of the manuscript. ATR reviewed and edited the manuscript. MS, EBN and MLS performed visualization studies. EBN and ATR supervised the study. ATR was responsible for project administration. All authors commented on and approved the final version of the manuscript. The assignment of order of the co–first authors was agreed upon by both MS and EBN. MS was assigned the first co-authorship position on the basis of his contribution as the primary author who conducted experiments, and EBN was assigned second co-authorship position on the basis of his contributions to project supervision, conceptualization of the study, and writing of the original draft of the manuscript and major revisions. The assignment of the order of the co–senior authors was agreed upon by both UJFT and ATR. ATR was assigned the first co–senior authorship position because the project was initiated, developed, and primarily performed in his laboratory. UJFT was assigned second co–senior authorship position because of his contribution of major clinical validation studies.

## Supplementary Material

Supplemental data

ICMJE disclosure forms

Supporting data values

## Figures and Tables

**Table 6:**
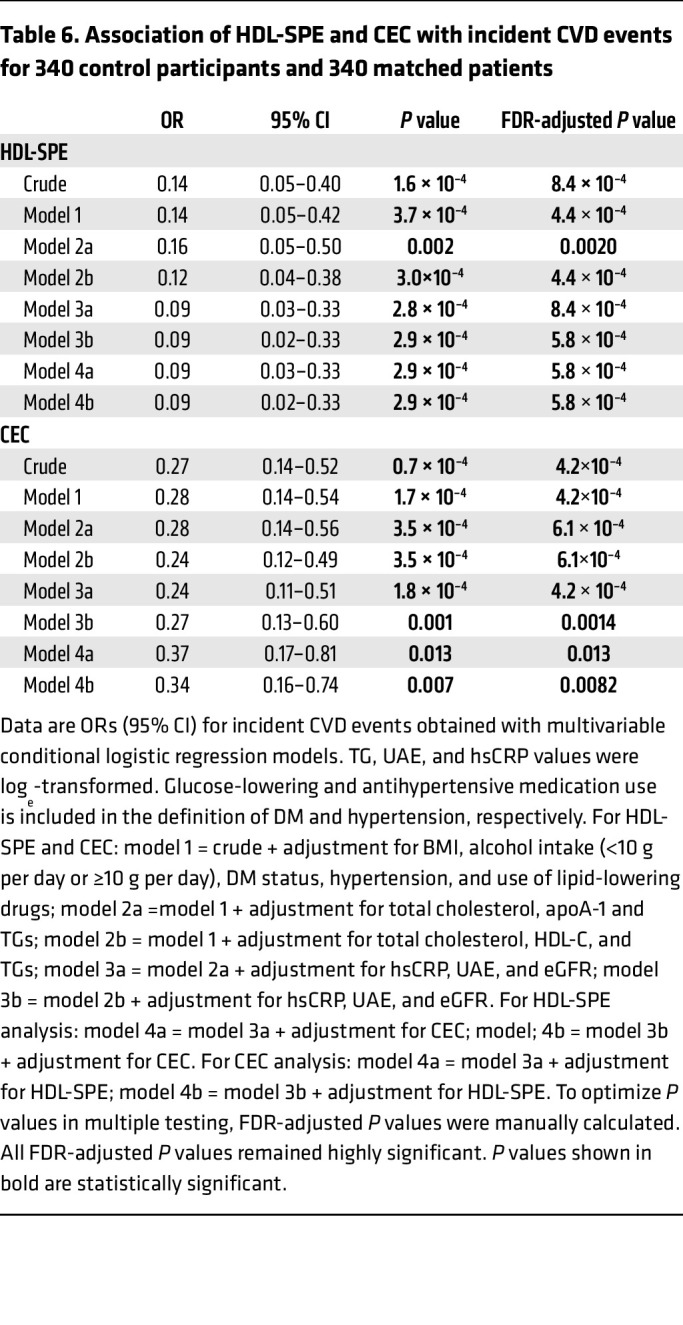
Association of HDL-SPE and CEC with incident CVD events for 340 control participants and 340 matched patients

**Table 5:**
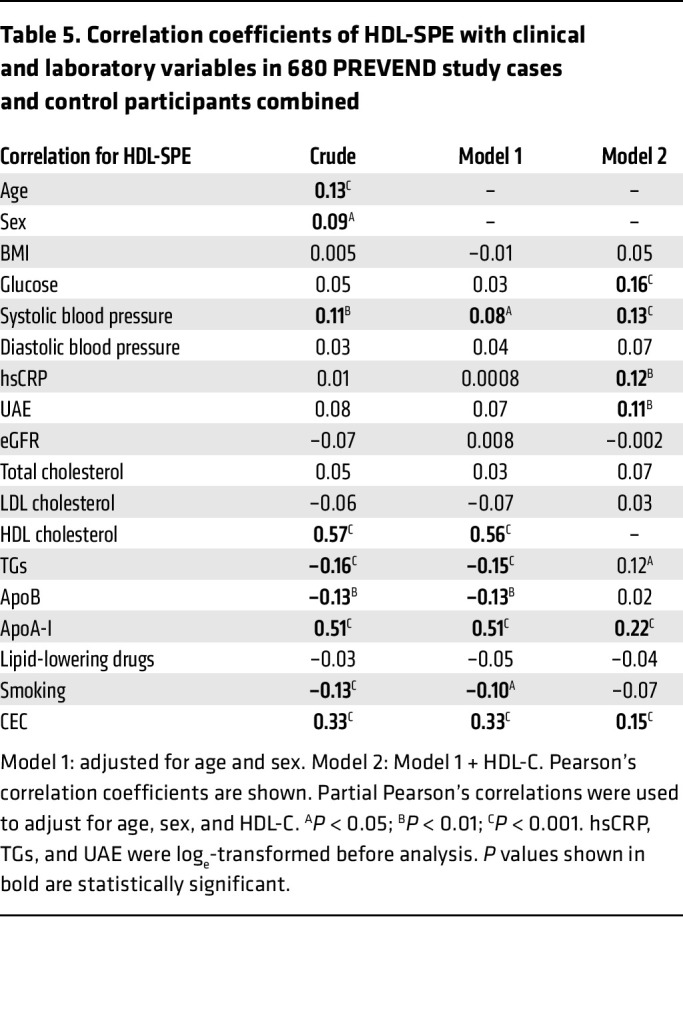
Correlation coefficients of HDL-SPE with clinical and laboratory variables in 680 PREVEND study cases and control participants combined

**Table 3:**
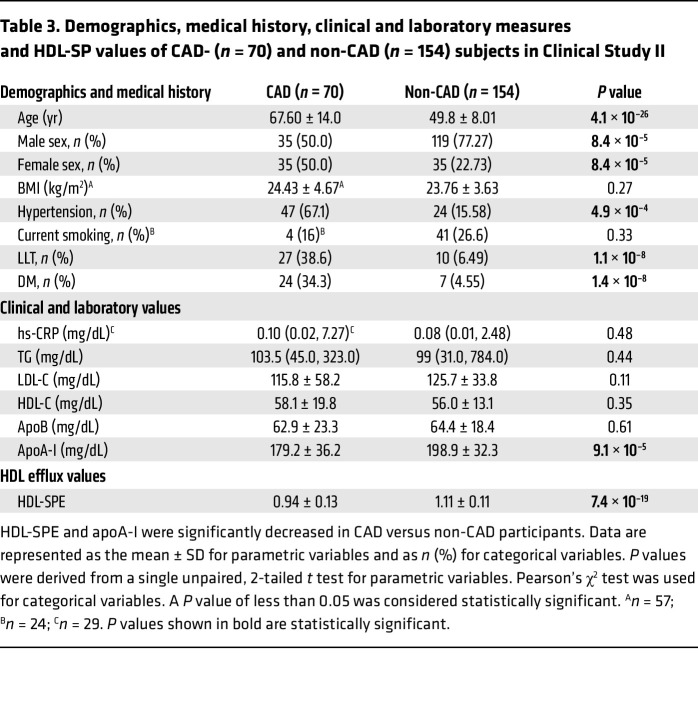
Demographics, medical history, clinical and laboratory measures and HDL-SP values of CAD- (*n* = 70) and non-CAD (*n* = 154) subjects in Clinical Study II

**Table 2:**
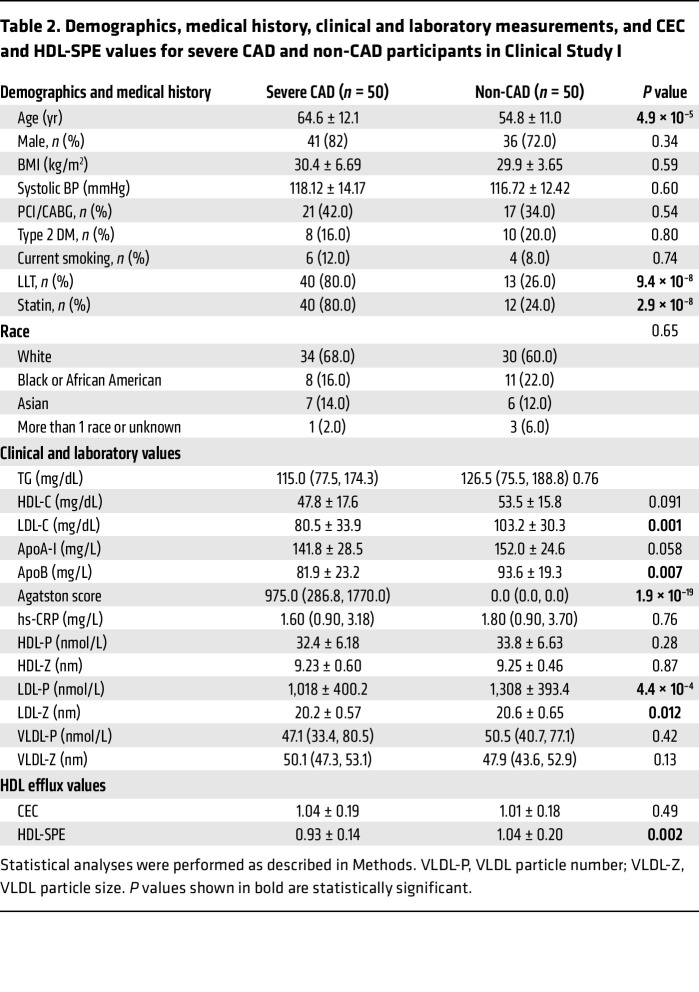
Demographics, medical history, clinical and laboratory measurements, and CEC and HDL-SPE values for severe CAD and non-CAD participants in Clinical Study I

**Table 1:**
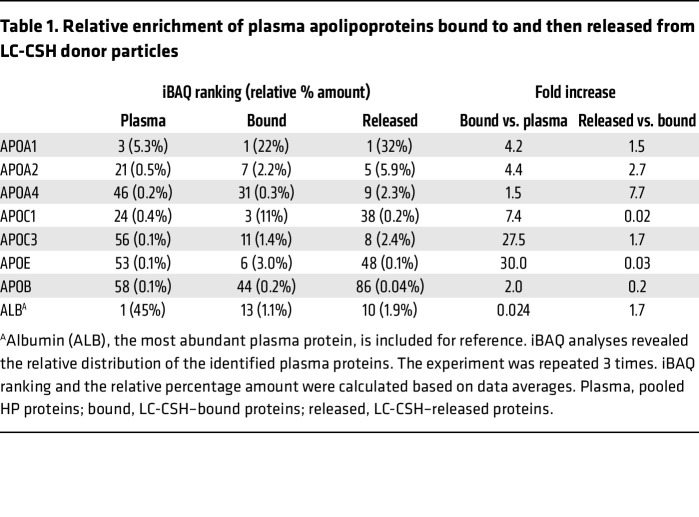
Relative enrichment of plasma apolipoproteins bound to and then released from LC-CSH donor particles

**Table 4:**
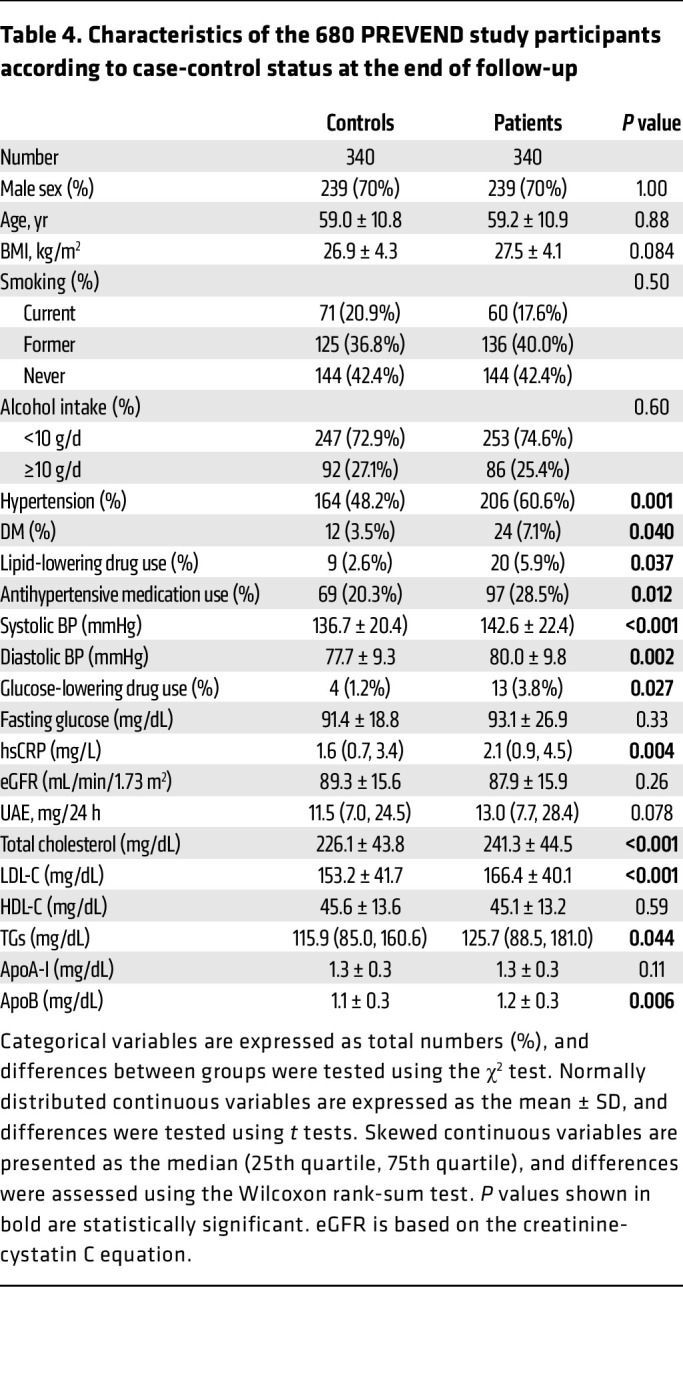
Characteristics of the 680 PREVEND study participants according to case-control status at the end of follow-up

## Version 1 07/20/2023

In-Press Preview

## Version 2 09/15/2023

Electronic publication
