# Supplementary material for: Cell-free, high-density lipoprotein–specific phospholipid efflux assay predicts incident cardiovascular disease
Source: J Clin Invest. 2023 Sep 15;133(18):e165370. doi: 10.1172/JCI165370 (PMC10503808; doi:10.1172/JCI165370)
Supplement: Supplemental data [file jci-133-165370-s169.pdf]

Cell-free high-density lipoprotein-specific phospholipid efflux assay predicts incident cardiovascular disease

Masaki Sato, Edward B. Neufeld, Martin P. Playford, Yu Lei, Alexander V. Sorokin, Angel M. Aponte, Lita A. Freeman, Scott M. Gordon, Amit K. Dey, Kianoush Jeiran, Masato Hamasaki, Maureen L. Sampson, Robert D. Shamburek, Jingrong Tang, Marcus Y. Chen, Kazuhiko Kotani, Josephine L.C. Anderson, Robin P.F. Dullaart, Nehal N. Mehta, Uwe JF Tietge, and Alan T. Remaley

Supplemental Material

Supplemental Methods

Plasma and serum samples. For analytical validation studies, pooled human plasma were obtained from healthy donors. VLDL, LDL, and HDL subfractions from normal human plasma were obtained by differential ultracentrifugation (1). For clinical validation studies, individual human serum samples were obtained from CVD patients enrolled in Clinical Trials at the NIH (Clinical Study I) and from Japanese CAD and non-CAD subjects (Clinical Study II). Blood collection was carried out following the rules of the Declaration of Helsinki of 1975 (https://www.wma.net/what-we-do/medical-ethics/declaration-of-helsinki/), revised in 2013. The NIH study was approved by the National Heart, Lung, and Blood Institute, Institute Review Board. Japanese study was approved by the Ethics Committee of Jichi Medical University. All subjects provided informed consent prior to participation in these studies. Direct labeling of all lipoproteins in whole pooled human plasma with fluorescent lipids was done as follows: 2 µL of *PE in ethanol and then 2 µL of *Chol in ethanol was injected into 200 µL pooled human plasma while vigorously mixing, as previously described (2).

Cholesterol efflux capacity assay (CEC). The CEC assay was performed as previously reported (3). Briefly, J774 cells (J774A.1, TIB-67™, Gaithersburg, MD; murine female) were plated and radiolabeled with 2 µCi of ^{3}H-cholesterol/mL and incubated with cAMP to upregulate ABCA1. PEG precipitated plasma was added to the efflux medium (final concentration 2.8%) for 4 hours. Efflux was calculated by using the following formula (µCi ^{3}H-cholesterol): ((^{3}H-cholesterol in subject's plasma) – (^{3}H-cholesterol in plasma-free media) /(^{3}H-cholesterol in media containing reference plasma pool) – (^{3}H-cholesterol in plasma-free media)). The pooled human plasma was obtained from healthy volunteers. All assays were performed in duplicate.

Gel Electrophoresis

Fluorescent Lipid Agarose Gel Electrophoresis. This assay was performed as previously reported (23). Briefly, fluorescent lipoproteins were monitored by electrophoresis of 10 µl of reaction mixture, using Sebia Hydragel Lipoprotein(E) 15/30 agarose gels (Sebia #4134), which ran at 100V for 60 min at room temperature. Fluorescent bands on the gel were imaged using a Typhoon 9400 Variable Mode Imager (GE). Lipoprotein labeled with *PE and/or *Chol were detected using excitation/emission wavelengths of 532/560nm and 488/520 nm, respectively. Following imaging of fluorescent lipids, gels were stained with Sudan Black staining according to manufacturer's instructions and rescanned. Quantitative analysis of fluorescent band intensity was performed using ImageQuant 5.1 software. Individual pixel intensities were normalized to total HDL pixel intensity for each sample.

Native gel analysis. An equal volume of Novex Tris–Glycine Native Sample Buffer (2X) (Thermo Fisher Scientific) was added to each sample and 20 µl was immediately loaded onto Novex native 10–20% Tris-Glycine WedgeWell minigels, 1.0 mm thick (Thermo Fisher Scientific). Amersham HMW Calibration Kit for Native Gel Electrophoresis (Catalog # 17-0445-01 GE Healthcare Piscataway, NJ) was used for the HDL size marker (8.16nm, 9.7nm, 12.2nm and 17nm). Gels were run at 35V for 17 hours and fluorescent bands were imaged using a Typhoon 9400 Variable Mode Imager (GE) and *PE and *Chol was detected using excitation/emission wavelengths of 532/560 nm and 488/520 nm, respectively. Following imaging of fluorescent lipids, gels were stained with Sudan Black staining and rescanned.

Proteomics

Assessment of LC-CSH-bound and released plasma proteins. To allow binding of plasma proteins to LC-CSH particles, 25 µL of pooled human plasma (or saline), 50 µL of LC-CSH and, 75 µL of saline were incubated for 30 min at 37 °C with shaking at 1200 rpm. 120 µL of supernatant was removed and replaced with 120 µL of new cold saline. After pipetting vigorously 5 times, LC- CSH was precipitated by centrifugation (2000 rpm for 2 min), and 120 µL of the supernatant was removed and replaced with 120 µL of new cold saline. This procedure was repeated for a total of five washes. All washing procedures were performed at 4 °C. We determined that five washes were sufficient to eliminate unbound plasma proteins (see below). The final saline supernatant wash was removed and 120 µL saline was added to the washed LC-CSH pellet (30 µL volume) so that the total final reaction mixture volume was 150 µL. Plasma proteins bound to LC-CSH were then allowed to be released into saline by incubating the mixture for 1h at 37 °C with shaking at 1200 rpm. For In-solution digest (Figure 3, G-I), proteins in the supernatant were concentrated via speed-vac to obtain sufficient protein mass for LC-MS/MS analyses. For fluorescent lipid gel electrophoresis (Figure 3, D-F), *PE labeled-HDL in the supernatant was concentrated by centrifugation (10,000 rpm, 45 min) using a 3K filter (Millipore #UFC500324) in order to enhance the *PE intensity to allow for gel analysis.

In-solution proteomic analysis. Whole plasma proteins, LC-CSH-bound-proteins and concentrated LC-CSH-released proteins were solubilized in 100 µL of lysis buffer (7M Urea / 2M thiourea in 50 mM TEAB). Bradford protein assay (23200, Thermo Scientific Pierce) was used to measure the protein concentrations for each sample. 40 µg of total protein from each sample were delipidated and concentrated using chloroform/methanol procedure adapted from a

previous report (4). Delipidated protein precipitates were resuspended in 100μL of 100 mM triethylammonium bicarbonate (TEAB), then reduced, alkylated and digested overnight with trypsin. Digested peptides were desalted, concentrated and purified using high capacity C18 tips according to the manufacturer's protocol (87784, Thermo Scientific Pierce) and transferred into sample vials for mass spectrometry.

Mass Spectrometry. All mass spectrometry experiments were performed in replicates on an Orbitrap Lumos Tribrid coupled with a Ultimate 3000-nLC (Thermo Fisher Scientific). Peptides were separated on an EASY-Spray C_{18} column (Thermo Scientific; 75 μm x 50 cm inner diameter, 2 μm particle size and 100 Å pore size). Separation was achieved by 4–35% linear gradient of acetonitrile + 0.1% formic acid over 125 min. An electrospray voltage of 1.9 kV was applied to the eluent via the EASY-Spray column electrode. The Orbitrap Lumos was operated in positive ion data-dependent mode. Full scan MS^1 was performed in the Orbitrap with a normal precursor mass range of 380–1,500 *m/z* at a resolution of 120k. The automatic gain control (AGC) target and maximum accumulation time settings were set to 4×10^5 and 50 ms, respectively. MS^2 was triggered by selecting the most intense precursor ions above an intensity threshold of 2.5×10^4 for collision-induced dissociation (CID)-MS^2 fragmentation with an AGC target and maximum accumulation time settings of 5×10^4 and 50 ms, respectively. Mass filtering was performed by the quadrupole with 0.7 *m/z* transmission window, followed by CID fragmentation in the Orbitrap and a normalized collision energy (NCE) of 35% at a resolution of 15k. To improve the spectral acquisition rate, parallelizable time was activated. The number of MS^2 spectra acquired between full scans was restricted to a duty cycle of 3 s.

Proteomic Data Processing. Raw data files were processed using Andromeda integrated in MaxQuant (v1.6.2.10, Max Planck Institute of Biochemistry) (5). All the peak lists were searched against the UniProtKB/Swiss-Prot protein database released 2019_04 with Homo sapiens taxonomy (20,316 sequences) and concatenated with reversed copies of all sequences. The following search parameters were set for MS1 tolerance of 10 ppm; orbitrap-detected MS/MS mass tolerance of 20 ppm; enzyme specificity was set as trypsin with maximum two missed cleavages; minimum peptide length of 7 amino acids; carbamidomethylation of cysteine was set as a fixed modification; and oxidation of methionine was set as a variable modification. Data were filtered to a 1% false discovery rate (FDR) on PSMs estimated using the decoy hit distribution. To calculate the approximate abundance of each protein we used the intensity based absolute quantification, or iBAQ (6) algorithm provided in MaxQuant. iBAQ is the sum of the extracted ion intensities of all identified peptides per protein, normalized by the number of theoretically observable peptides of the protein. These normalized protein intensities are translated to protein copy number estimates based on the overall protein amount in the analyzed sample.

Clinical Studies

Normalization of HDL-SPE efflux values for clinical studies. Normalized efflux values were defined as clinical sample efflux value (FLU.) divided by reference control efflux value (FLU.). Identical pooled human plasma from healthy donors with normal lipid profile was used as a reference control. Normalization of efflux values in clinical studies was performed to correct for any variation in reagent preparation and experimentation.

Case/control clinical study I and II populations. Clinical study I consisted of subjects from an independently selected subset from an ongoing clinical investigation of new radiographic techniques for cardiovascular imaging performed at the NIH Clinical Center (ClinicalTrials.gov identifier: NCT01621594). Coronary CT scans were performed using a 320-detector row Aquilion ONE ViSION system (Toshiba). This Parent Study (n=860) at the time of subset selection for Clinical Study I included both males and females that were at least 18 years of age and with clinical indication for CCTA exam. There were no additional inclusion criteria. Exclusion criteria included current pregnancy and severe renal dysfunction. For HDL-SPE clinical performance evaluation, according to Coronary Artery Disease Reporting and Data System (CADRADS) classification based on CCTA exam, n=385 subjects (CADRADS 2-4 and other CVD abnormalities) were excluded from the parent CVD cohort and severe CAD subjects defined as severe stenosis/obstructive disease patients with based on the angiographical results (CAD-RADS 4/5) and Non-CAD/Non-obstructive CAD subjects defined as with no significant stenosis/minimum stenosis (CADRADS 0/1) were recruited as shown in Figure 5A. Non-CAD/Non-obstructive CAD subjects were followed by selection via matching based on gender, BMI, DM2, and Smoking. We also aimed to keep age differences between groups minimal, but we were not able to fully match by age because of the CVD epidemiological natural distribution and limited number of patients. Finally, 50 severe CAD subjects and 50 non-CAD/non-obstructive CAD subjects were used for CEC and HDL-SPE assay. Abbreviations: Systoltic BP: Systolic blood pressure, PCI/CABG: percutaneous coronary intervention or coronary artery bypass grafting, DM2: Type2-diabetes mellitus, Current SMK: Current smoker, LLT: Lipid lowering therapy, TG: triglyceride, HDL-C: HDL cholesterol, LDL-C: LDL cholesterol, hs-CRP: high sensitivity C-reactive protein, HDL-P: HDL particle number, HDL-

Z: HDL size, LDL-P: LDL particle number, LDL-Z: LDL size, VLDL-P: VLDL particle number, VLDL-Z: LDL size.

For Clinical Study II, we studied an additional ethnic group study, which included 224 subjects of a Japanese cohort, consisting of CAD cases (n=70) and non-CAD (n=154). This was a cross-sectional research study and subjects included both males and females that were at least 20 years of age with, or at risk for, lifestyle-related diseases, with no additional inclusion criteria. A total 267 of subjects were enrolled in this study. For our investigations, 50 subjects were excluded due to missing data concerning the age, gender, HDL-cholesterol levels and lipid lowering therapy of these subjects. CAD was evaluated based on coronary angiography. Strengthening the reporting of observational studies in epidemiology guidelines were followed for reporting the findings (7). All patient samples were collected in clinical trials with written informed consents.

Prevention of REnal and Vascular End stage Disease (PREVEND) study set-up. HDL-SPE was measured in a nested case-control study constructed from the participants of PREVEND as detailed previously (8). Briefly, with the aim to investigate the association of renal damage with CV disease in a large general population cohort in the period between 1997-98, all inhabitants of the city of Groningen aged 28-75 years (a total of 85,421 participants) were sent a questionnaire containing information about the presence of CVD risk factors and morbidity and a vial to collect an early morning urine sample. In total, 40,856 participants (47.8%) responded. All participants with a urinary albumin concentration ≥ 10 mg/L were invited to the clinic together with randomly selected participants with a urinary albumin concentration < 10 mg/L. Individuals with type 1 or type 2 diabetes using insulin and pregnant women were excluded. The study comprised 8,592

predominantly White participants who completed the total screening program. For the current work, first, all participants with a CVD event prior to inclusion were excluded. Cases were identified as participants who had a first CVD event before the end of follow-up (January 1, 2009). Cases (n=369) were then stratified according to sex and current smoking behavior at baseline and divided into HDL cholesterol quartiles. Each case was matched to a control participant of the same sex, same smoking status, age and HDL cholesterol level. Actual plasma samples for were available in 357 controls and 352 cases resulting in 340 matched case-control pairs. Median follow-up time was not different in cases (10.5 [9.9–10.8] years) and in controls (10.4 [9.9–10.8] years). For detailed descriptions of laboratory methods, definitions and procedures with respect to the baseline characterization, please see our previous publication (8). The CEC assay was performed using THP1-derived macrophage foam cells (ATTC via LCG Promochem, Teddington, UK; male) and an efflux time of 5 hours, as previously described. (8)

Outcome measures. The combined endpoint of the study was incident CVD, defined as death from CVD, hospitalization for myocardial infarction (MI), percutaneous transluminal coronary angioplasty (PTCA), ischemic heart disease or coronary artery bypass graft (CABG). From the time of inclusion in the study, the vital status of the participants was checked through the municipal registry. The cause of death was obtained by linking the number of the death certificates to the primary cause of death, as coded by a physician from the Central Bureau of Statistics (CBS, Voorburg/Heerlen, The Netherlands). Causes of death were coded according to the 9th revision of the International Classification of Diseases (ICD-9. Information on MI (ICD-9 code 410), PTCA (code 45), ischemic heart disease (code 411) and CABG (code 414) was obtained from the

national hospital information system (Prismant, Utrecht, The Netherlands). The censoring date was the date obtained from the municipal registry or date of death.

Determination of HDL-SPE for PREVEND plasma samples. Assays were shipped on dry ice from the NIH in a ready-to-use set-up with reagents pre-pipetted into 96-well plates and kept frozen until use. Before use plates were thawed while being centrifuged. Following the detailed protocol described in "Methods", using a total volume of 150 µL per assay, containing 50 µL of LC-CSH and 75 µL saline, HDL-SPE was determined in duplicates, adding 15 µl of individual plasma samples as acceptor and 10 µl saline (total volume 25 µl). Results were normalized to an internal control, a pool of plasma from healthy control subjects, present on each plate.

Methods References

1. Brewer HB, Jr., Ronan R, Meng M, and Bishop C. Isolation and characterization of apolipoproteins A-I, A-II, and A-IV. *Methods Enzymol.* 1986;128:223-46.
2. Neufeld EB, Sato M, Gordon SM, Durbhakula V, Francone N, Aponte A, et al. ApoA-I-Mediated Lipoprotein Remodeling Monitored with a Fluorescent Phospholipid. *Biology (Basel).* 2019;8(3).
3. Gordon SM, Chung JH, Playford MP, Dey AK, Sviridov D, Seifuddin F, et al. High density lipoprotein proteome is associated with cardiovascular risk factors and atherosclerosis burden as evaluated by coronary CT angiography. *Atherosclerosis.* 2018;278:278-85.
4. Wessel D, and Flugge UI. A method for the quantitative recovery of protein in dilute solution in the presence of detergents and lipids. *Anal Biochem.* 1984;138(1):141-3.
5. Cox J, and Mann M. MaxQuant enables high peptide identification rates, individualized p.p.b.-range mass accuracies and proteome-wide protein quantification. *Nat Biotechnol.* 2008;26(12):1367-72.
6. Schwanhausser B, Busse D, Li N, Dittmar G, Schuchhardt J, Wolf J, et al. Global quantification of mammalian gene expression control. *Nature.* 2011;473(7347):337-42.
7. von Elm E, Altman DG, Egger M, Pocock SJ, Gøtzsche PC, and Vandenbroucke JP. The Strengthening the Reporting of Observational Studies in Epidemiology (STROBE) statement: guidelines for reporting observational studies. Ann Intern Med. 2007;147(8):573-7
8. Ebtehaj S, Gruppen EG, Bakker SJL, Dullaart RPF, and Tietge UJF. HDL (High-Density Lipoprotein) Cholesterol Efflux Capacity Is Associated With Incident Cardiovascular Disease in the General Population. *Arteriosclerosis, thrombosis, and vascular biology.* 2019;39(9):1874-83.

Supplemental Figure 1. Development of functional LC-CSH with minimal lipid composition for optimized HDL-SPE.

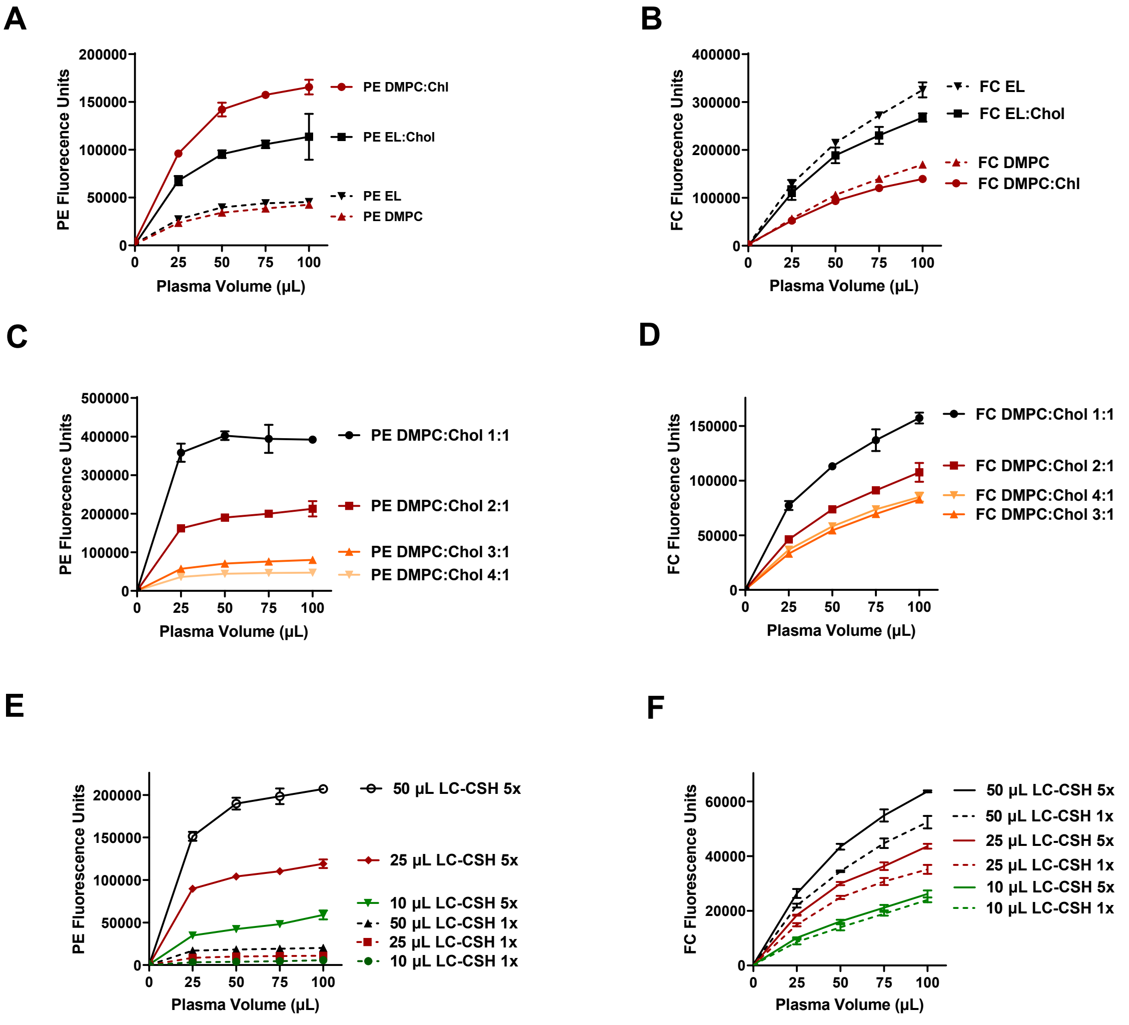

G

LC-CSH labeling and coating

LC-CSH	20	40	60	80
DMPC	11.8	11.8	11.8	11.8
Cholesterol	3.39	3.39	3.39	3.39
*PE	0.305	0.305	0.305	0.305
*Chol	0.028	0.028	0.028	0.028

Unit: mg

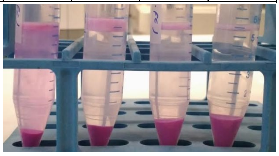

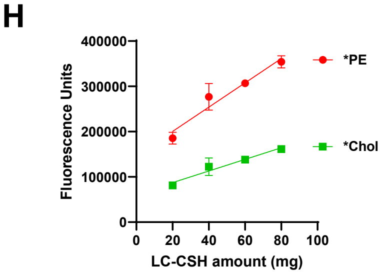

Supplemental Figure 1. Development of functional LC-CSH with minimal lipid composition for optimized HDL-SPE.

Comparison of HDL-SPE using LC-CSH coated with DMPC or egg lecithin phospholipid with or without cholesterol. (A) LC-CSH (80 mg) was coated with DMPC or egg lecithin (EL) alone or in combination with cholesterol at a 4:1 mole ratio (phospholipid/cholesterol) in 2 mL saline containing both fluorescent phosphatidylethanolamine (*PE) and cholesterol (*Chol) and then incubated with 0, 25, 50, 75, or, 100 µL pooled human plasma for 1 h at 37 °C at 1200 RPM. Relatively small amounts of fluorescent PE transferred to plasma from LC-CSH coated with either DMPC or EL phospholipids alone, but PE efflux was markedly enhanced in the presence of cholesterol on LC-CSH. Fluorescent PE transfer from cholesterol-containing LC-CSH to plasma was dependent on phospholipid and was more robust from DMPC- than from EL-containing LC-CSH. Based on these findings DMPC was selected as the phospholipid of choice, and 25 µL or less as the preferred volume of human plasma. **(B)** Cholesterol transfer to plasma was dependent upon the phospholipid composition of donor particles. In marked contrast to *PE efflux, the presence of cholesterol had little effect on *Chol transfer to plasma. LC-CSH containing EL were a better *Chol donor than those containing DMPC, with or without cholesterol. Fluorescent PE efflux from LC-CSH to plasma appeared to saturate in the presence of 50–100µL plasma, whereas Bodipy-cholesterol efflux appeared to be relatively linear across the range of plasma concentrations. Together, these findings suggest different mechanisms underlie phospholipid and cholesterol efflux from donor particles to plasma.

Effect of DMPC:cholesterol mole ratio on fluorescent lipid transfer to human plasma. (C) Pooled human plasma at the indicated volumes were incubated with *PE- and *Chol-labeled LC-CSH coated with different mole ratios of DMPC:cholesterol, as shown, for 1 h at 37 °C at 1200 RPM. *PE and *Chol fluorescence in the supernatant were measured. Although DMPC:Cholesterol 1:1 provided maximal signal intensity, DMPC:Cholesterol 2:1 provided sufficient signal intensity with more highly specific HDL efflux. DMPC:Cholesterol 2:1 markedly increased *PE efflux compared to 3:1 or 4:1 consistent with increased lipid packing defects on the LC-CSH lipid surface. Regardless of the DMPC:cholesterol ratio, saturation of plasma *PE signal occurred at 25 µL plasma volume. **(D**) Interestingly**,** *Chol efflux was increased to a lesser degree with increased DMPC:cholesterol ratio than *PE efflux was. *Chol efflux was nearly linear throughout the range plasma volume range used in all cases.

Supplemental Figure 1. Development of functional LC-CSH with minimal lipid composition for optimized HDL-SPE.

Effect of LC-CSH fluorescent lipid mass on fluorescent lipid transfer to human plasma. (E) LC-CSH was coated with DMPC:Cholesterol:*PE:*Chol mole ratio = 2:1:0.02:0.01 or 2:1:0.004:0.002. 10, 25, or 50 µL of LC-CSH were incubated with the indicated volumes of pooled human plasma for 1 h at 37 °C at 1200 RPM. The 5-fold increase in LC-CSH fluorescent lipid content markedly increased fluorescent PE efflux to plasma, **(F)** but only marginally increased fluorescent cholesterol efflux to plasma. We routinely used LC-CSH containing the 5-fold increased fluorescent PE mass in our studies, as it provided a robust fluorescent PE plasma signal in the assay.

Effect of LC-CSH lipid mass/LC-CSH mass ratio on LC-CSH phospholipid and cholesterol fluorescent lipid content of LC-CSH. (G) To determine the optimal mass of lipid that can fully coat LC-CSH, different masses of LC-CSH (20, 40, 60, or 80 mg) were coated with the same amount of total lipid mass, and **(H)** *PE and *Chol fluorescence were measured after extraction of LC-CSH lipids (10µL of LC-CSH was mixed with 190 µL of 1% TX-100 and 100 µL of the extract was used for measurement of fluorescence. As shown in **(H)**, lipid fluorescence increased in a linear manner with increasing LC-CSH mass. As shown in the photo in (g), 80 mg of LC-CSH appeared to be completely coated, whereas with decreasing LC-CSH mass, increasing amounts of *PE could be seen in the supernatant (pinkish color). These findings indicate that this amount of lipid fully coated 80 mg LC-CSH. Less lipid appeared to be required to fully coat smaller amounts of LC-CSH (20–60 mg) and the excess lipids accumulated in the aqueous phase.

Supplemental Figure 2. Structural and physiochemical characterization of LC-CSH and HDL-SPE.

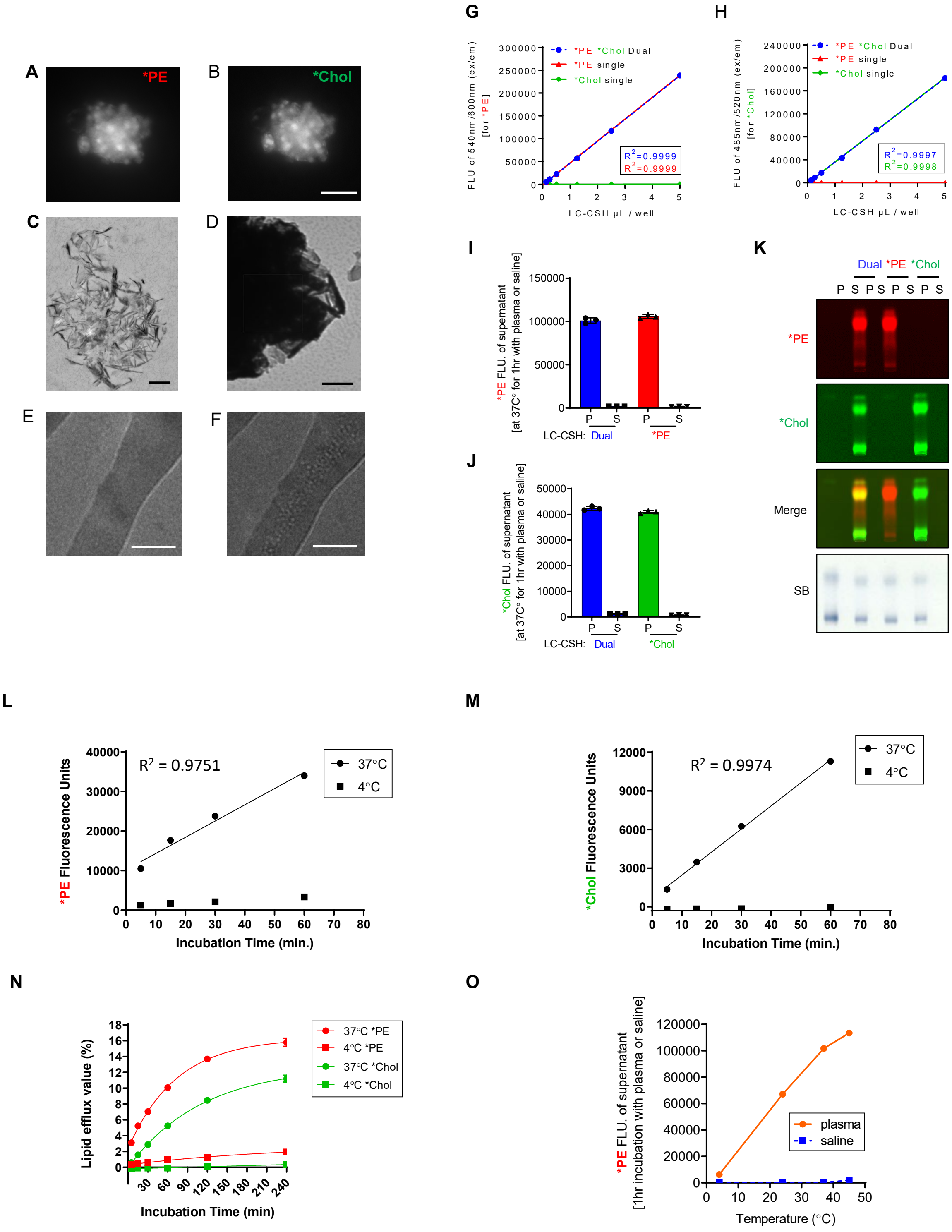

Supplemental Figure 2. Structural and physiochemical characterization of LC-CSH and HDL-SPE.

Confocal and electron microscopy of LC-CSH. High resolution confocal microscopy of dual *PE- and *Chol-labeled LC-CSH reveals the distribution of **(A)** *PE and **(B)** *Chol on the surface of LC-CSH (Scale bar = 5 μm). **(C)** Transmission electron microscopy of LC-CSH thin-sections (Scale bar = 500 nm) and, **(D)** negatively-stained whole LC-CSH reveal plate and needle crystal structure (Scale bar = 500 nm). **(E,F)** The lipid coating on the surface of CSH crystals is revealed by high intensity electron irradiation. LC-CSH imaged before **(E),** and after high intensity electron irradiation. Note the bubbling (melted lipids) **(F)** on the surface of LC-CSH that occurs after irradiation. Scale bars = 50 nm.

LC-CSH, HDL-SPE supernatant and agarose gel *PE and *Chol fluorescence emission is unaltered using dual-labeled LC-CSH. Standard curves for LC-CSH singly or dually labeled with *PE and/or *Chol. **(G)** Fluorimetry using 540 nm/600 nm (Ex/Em). **(H)** Fluorimetry using 485 nm/520 nm (Ex/Em). FLU=Arbitrary fluorescence units. Note that LC-CSH *PE fluorescence emission is unaltered in the presence of *Chol **(G)** and conversely, *Chol fluorescence emission is unaltered in the presence of *PE **(H).** *PE-labeled LC-CSH has no *Chol emission **(G)** and conversely, *Chol-labeled LC-CSH has no *PE emission **(H).** **(I)** Only *PE fluorescence is detected in the supernatant of plasma (P) (but not saline (S)) incubated with either *PE-labeled or dually *PE/*Chol-labeled LC-CSH when *PE fluorescence is monitored. (**J**) Only *Chol fluorescence is detected in the supernatant of plasma (P) (but not saline (S)) incubated with either *Chol-labeled or dually *PE/*Chol-labeled LC-CSH when *Chol fluorescence is monitored. **(K)** Agarose gel electrophoresis of human plasma incubated with either dual (*PE/*Chol)- or *PE-, or *Chol-labeled CS-CSH under standard assay conditions. Note that no signal is detected in saline (S). Only *PE is detected on HDL with singly- or dually-fluorescent lipid-labeled LC-CSH when imaged for *PE fluorescence. Conversely, *Chol alone is detected on all lipoproteins with singly- or dually-fluorescent lipid-labeled LC-CSH when imaged for *Chol fluorescence. All data are mean ± SD in triplicate assays.

HDL-SPE Time and Temperature Dependency. HDL-SPE Time Dependency at 4 ˚C vs 37 ˚C. Pooled human plasma were incubated with fluorescent lipid labeled LC-CSH using standard HDL-SPE assay conditions, as described in “Methods”. Efflux of *PE fluorescence **(L)** and *Chol **(M)** was linear from 5 to 60 min at 37 ˚C. Little or no *PE **(L)** or *Chol **(M)** transferred from LC-CSH to plasma at 4 ˚C. **(N)** The % *PE and *Chol efflux saturated at 2 - 4 h at 37 ˚C with little or no efflux occurring between 5 min and 4 h at 4 ˚C. **(O)** HDL-SPE is temperature-dependent. Pooled human plasma or saline were incubated with fluorescent lipid labeled LC-CSH using standard HDL-SPE assay conditions, as described in “Methods.” FLU = fluorescence units. Note that *PE efflux to plasma is linear from 4 ˚C to 37 ˚C. All data are mean ± SD in triplicate assays.

Supplemental Figure 2. Structural and physiochemical characterization of LC-CSH and HDL-SPE.

Methods

Confocal microscopy. Structured Illumination Microscope (SIM) mages were acquired with a VT-iSIM Imaging System scanner from VisiTech International (Sunderland, UK) on an Olympus IX 81 microscope using an Olympus UPLSAPO 100 × 1.49 NA Oil objective and dual Hamamatsu CMOS Orca-Flash 4 cameras. The total acquisition system was controlled using Metamorph software (Molecular Devices, LLC, San Jose, CA). The *PE and *Chol fluorescence were excited with 561 nm and 488 nm lasers, respectively, and the emission light was filtered using long pass 590 nm, and 500-550 nm emission filters, respectively, before the camera. 3D volumes were taken at an interslice distance of 100 nm for a total of 30-36 individual planes. Exposure time for each plane was 250 ms.

Electron microscopy. Thin section TEM. LC-CSH particles were mixed with 10% liquefied low-melting point agarose and then solidified on ice. The embedded LC-CSH were maintained at 4 ˚C overnight and then fixed in 2.5% glutaraldehyde/1% paraformaldehyde in 0.12 M sodium cacodylate buffer (pH 7.4) at 4 ˚C overnight. Fixed samples were washed in cacodylate buffer, post-fixed in 1% OsO_4 in cacodylate buffer, washed, stained en bloc with uranyl acetate, ethanol dehydrated, and EMbed-812 embedded (Electron Microscopy Sciences, Hatfield PA). Thin sections were stained with uranyl acetate and lead citrate prior to imaging with a JEM1400 electron microscope (JEOL USA) equipped with an AMT XR-111 digital camera (Advanced Microscopy Techniques Corp).

Supplemental Figure 2. Structural and physiochemical characterization of LC-CSH and HDL-SPE.

PE and *Chol standard curves for HDL-SPE and NS-CEC. First, we prepared a 10-fold dilution of LC-CSH and a 100-fold dilution of LC-CSH in 15 mL tubes as follows: 10-fold dilution: 100 µL of LC-CSH and 900 µL of 1% Triton-X100 in water 100-fold dilution: 100 µL of 10-fold diluted LC-CSH and 900 µL of 1% Triton-X100. Second, we prepared six sequential dilutions in 96 well plate using 10-fold dilution and 100-fold dilution as follows: 100 µL of 10-fold dilution + 100 µL of 1% Triton-X100 (equivalent to 10 µL of LC-CSH per well), 50 µL of 10-fold dilution + 150 µL of 1% Triton-X100 (equivalent to 5 µL of LC-CSH per well), 25 µL of 10-fold dilution + 175 µL of 1% Triton-X100 (equivalent to 2.5 µL of LC-CSH per well), 100 µL of 100-fold dilution + 100 µL of 1% Triton-X100 (equivalent to 1 µL of LC-CSH per well), 50 µL of 100-fold dilution + 150 µL of 1% Triton-X100 (equivalent to 0.5 µL of LC-CSH per well), 25 µL of 100-fold dilution + 175 µL of 1% Triton-X100 (equivalent to 0.25 µL of LC-CSH per well). Third, to completely dissolve LC-CSH-bound lipids, we incubated the samples at 24 ˚C for 1 h with shaking (1200 rpm). Following this incubation, 100 µL of the entire suspension (including the delipidated CSH particles) was transferred to black 96 well plate wells along with 100 µL of saline. After mixing well, fluorescence of fluorescent-tagged PE (*PE) and/or fluorescent-tagged cholesterol (*Chol) were measured as described above. All assays were performed in triplicate. Note this protocol can provide precise standard curves for *PE (R^2=0.9999) and *Chol (R^2=0.9997). We confirmed that there is no cross-talk between *PE fluorescence and *Chol fluorescence using *PE singly-labeled LC-CSH or *Chol singly-labeled LC-CSH. Moreover, no interference in the measurement of either HDL-SPE or NS-CEC was detected when dual *PE- and *Chol-labeled LC-CSH donor particles were used in the assay.

Supplemental Figure 3. Human plasma and human serum are both suitable to assess HDL-SPE.

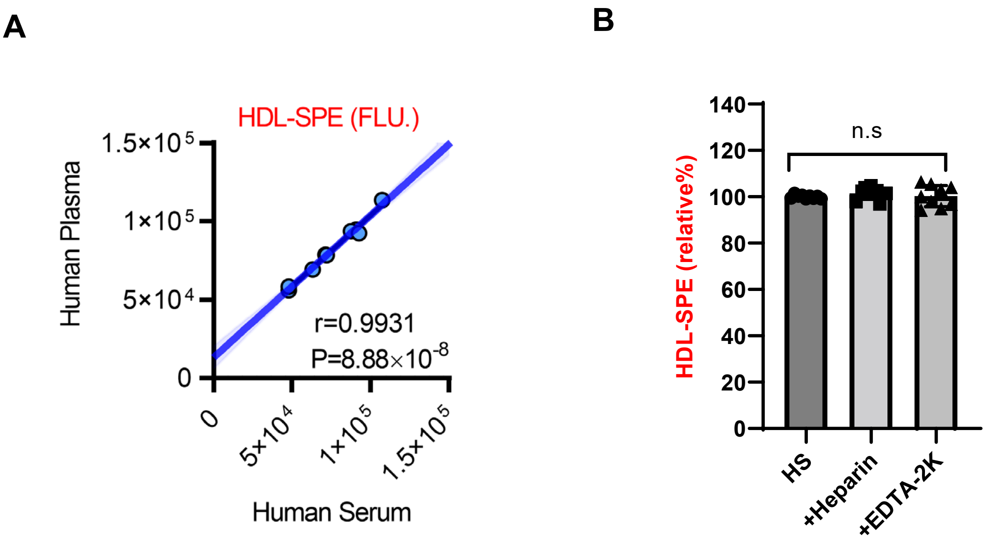

(**A)) HDL-SPE by human plasma and serum highly correlate** (triplicate paired 15, 25 and 35 µL serum and heparin-plasma samples from 3 individuals). **(B) The effect of anticoagulants heparin-sodium and EDTA-2K on HDL-SPE.** Heparin-sodium was used with final concentration of 200 USP(1 mg) per 10 mL serum and EDTA-2K with 0.1% weight per volume. These are the working anticoagulant concentrations typically employed. HDL-SPE relative% is the relative value based on using HDL-SPE in control HS as the 100% baseline. The data represent the mean of triplicate assays in three independent experiments (n=9). Statistical analysis was performed by one-way ANOVA followed by multiple comparison test. $p<0.05$ was considered statistically significant.

Supplemental Figure 4. HDL-SPE assay precision and stability.

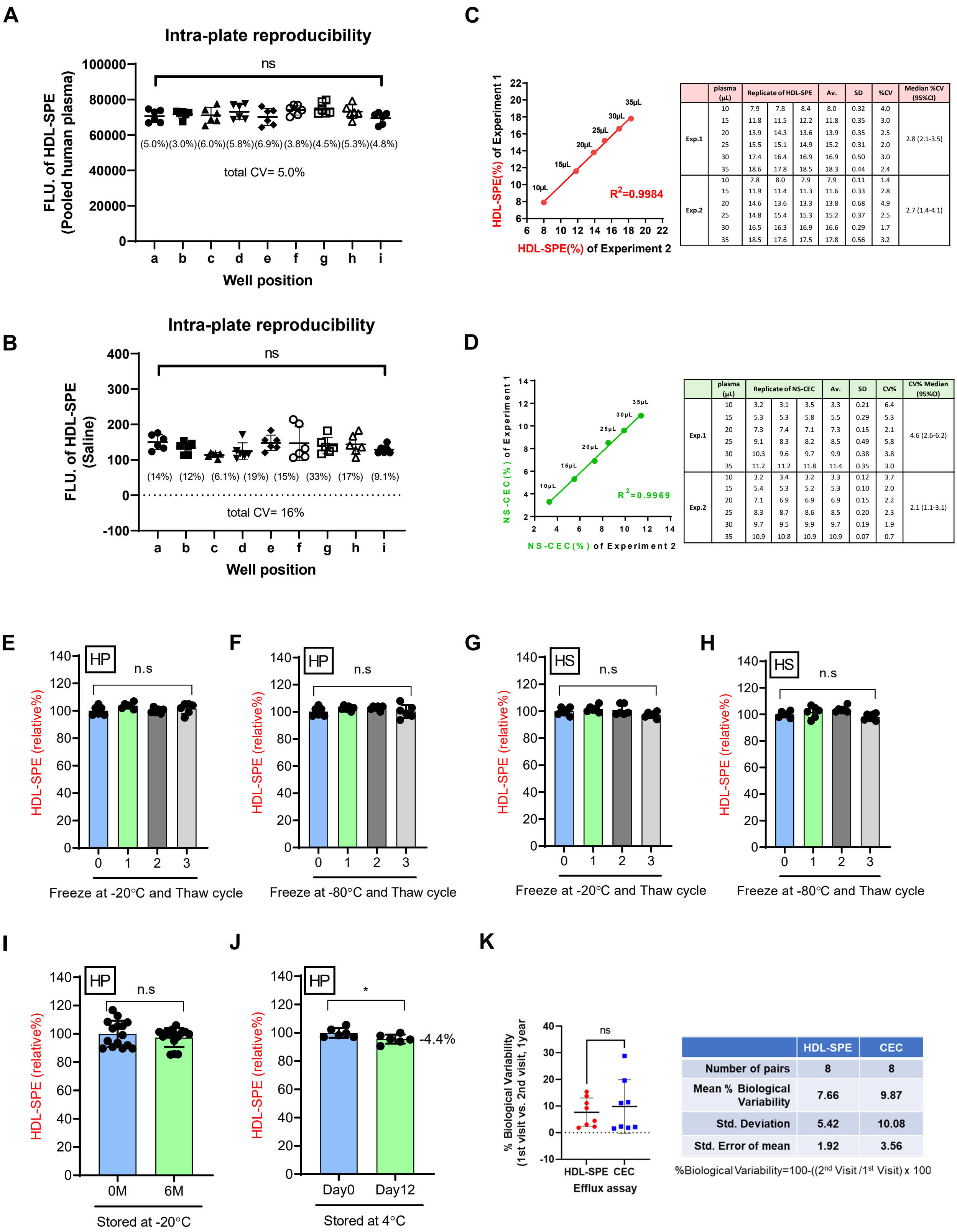

Panel C table:

	plasma (μL)	Replicate of HDL-SPE			Av.	SD	%CV	Median %CV (95%CI)
Exp.1	10	7.9	7.8	8.4	8.0	0.32	4.0	2.8 (2.1-3.5)
	15	11.8	11.5	12.2	11.8	0.35	3.0	
	20	13.9	14.3	13.6	13.9	0.35	2.5	
	25	15.5	15.1	14.9	15.2	0.31	2.0	
	30	17.4	16.4	16.9	16.9	0.50	3.0	
	35	18.6	17.8	18.5	18.3	0.44	2.4	
Exp.2	10	7.8	8.0	7.9	7.9	0.11	1.4	2.7 (1.4-4.1)
	15	11.9	11.4	11.3	11.6	0.33	2.8	
	20	14.6	13.6	13.3	13.8	0.68	4.9	
	25	14.8	15.4	15.3	15.2	0.37	2.5	
	30	16.5	16.3	16.9	16.6	0.29	1.7	
	35	18.5	17.6	17.5	17.8	0.56	3.2	

Panel D table:

	plasma (μL)	Replicate of NS-CEC			Av.	SD	CV%	CV% Median (95%CI)
Exp.1	10	3.2	3.1	3.5	3.3	0.21	6.4	4.6 (2.6-6.2)
	15	5.3	5.3	5.8	5.5	0.29	5.3	
	20	7.3	7.4	7.1	7.3	0.15	2.1	
	25	9.1	8.3	8.2	8.5	0.49	5.8	
	30	10.3	9.6	9.7	9.9	0.38	3.8	
	35	11.2	11.2	11.8	11.4	0.35	3.0	
Exp.2	10	3.2	3.4	3.2	3.3	0.12	3.7	2.1 (1.1-3.1)
	15	5.4	5.3	5.2	5.3	0.10	2.0	
	20	7.1	6.9	6.9	6.9	0.15	2.2	
	25	8.3	8.7	8.6	8.5	0.20	2.3	
	30	9.7	9.5	9.9	9.7	0.19	1.9	
	35	10.9	10.8	10.9	10.9	0.07	0.7	

Panel K table:

	HDL-SPE	CEC
Number of pairs	8	8
Mean % Biological Variability	7.66	9.87
Std. Deviation	5.42	10.08
Std. Error of mean	1.92	3.56

%Biological Variability=100-((2nd Visit / 1st Visit) x 100

Supplemental Figure 4. HDL-SPE assay precision and stability.

These reproducibility analyses were conducted using the same batch of materials including LC-CSH.

Intra-plate reproducibility. The same lot of LC-CSH was used for 3 plates**.** HDL-SPE was performed using **(A)** pooled human plasma or **(B)** saline duplicates at nine different positions encompassing the entire plate. Intra-plate reproducibility was low (%CV in N=6 wells in each position) of N=3 plates performed within the same day suggests no well-position dependency in HDL-SPE assay. All data are mean ± SD in N=6 plots. Ordinary one-way ANOVA was used. *p<0.05 was considered statistically significant.

Intra- and inter-plate variability for HDL-SPE and NS-CEC assays. Experiments 1 (y-axis) and 2 (x-axis) were conducted using the same pooled plasma samples and LC-CSH batch on different days. **(C)** HDL-SPE assay. **(D)** NS-CEC assay. Note the highly significant linearity (low inter-plate variability) and the low intra-plate median %CV for HDL-SPE and NS-CEC in **(C)** and **(D)**, respectively.

Plasma and serum storage stability. The effect of freeze and thaw cycles on HDL-SPE **(E,G)** at -20 °C; or at -80 °C **(F,H)** was evaluated using **(E,F)** human pooled plasma (HP) and **(G,H)** human pooled serum (HS). Thawing was performed in a room-temperature in water-bath. HDL-SPE assay was performed with triplicate assay in two independent experiments. HDL-SPE assay using HP after long-storage for **(I)** 6 months at -20 °C and **(J)** after short-storage for 12 days at 4 °C was performed. HDL-SPE relative% is the relative value based on using HDL-SPE in control HP/HS with 0 freeze/thaw cycles as the 100% baseline in **(E-H)** and 0 M (months) as control in **(I),** and Day 0 as control in **(J)**. Each point represents the mean of triplicate assays in two independent experiments (n=6) in **(E-H,J)** and the mean of triplicate assay in five independent experiments (n=15) in **(I)**. Statistical analysis was done by one-way ANOVA in **(E-H)** and by unpaired t-test in **(I,J)**. *p<0.05 was considered statistically significant.

Biological Variability of HDL-SPE. (K) HDL-SPE and CEC were performed on triplicate plasma samples from eight individuals obtained 1 year apart. The baseline and 1-year samples were assayed on individual plates. Statistical analysis was done using unpaired t-test.

Supplemental Figure 5. LC-CSH batch comparison and storage stability.

A

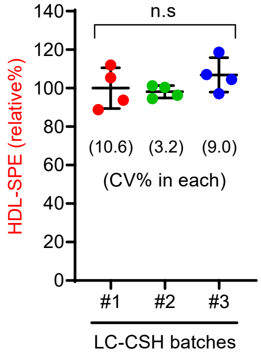

B

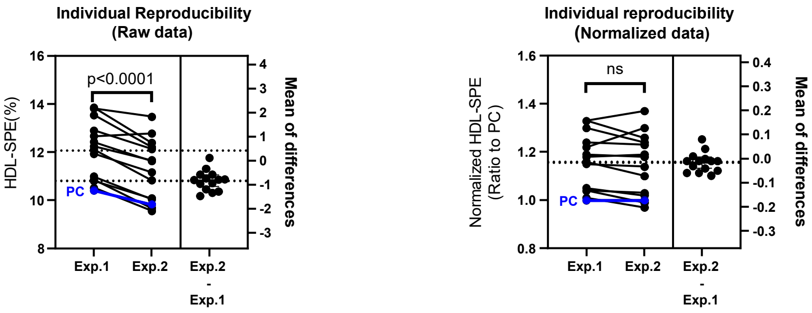

C

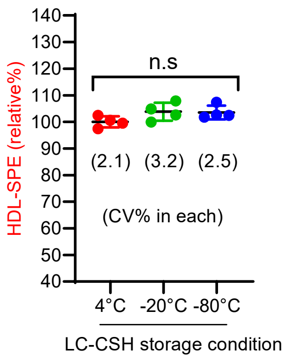

D

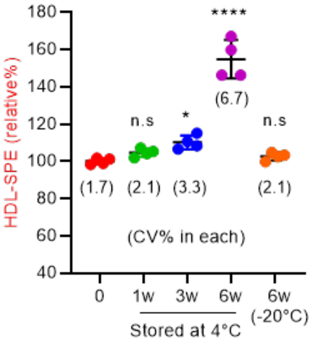

Supplemental Figure 5. LC-CSH batch comparison and storage stability.

(A) LC-CSH batch comparison. HDL-SPE assay using control pooled human plasma (HP) was performed comparing three different batches of LC-CSH which were prepared on different days (day 14 (batch #1), day 7 (batch #2) and day 4 (batch #3)) and stored at 4 °C until use. Four individual LC-CSH preparations (lots) were included in each batch. Each point represents the mean of triplicate assay samples.

(B) Normalized HDL-SPE reproducibility study for individual samples using different batches of LC-CSH. Duplicate samples of individual healthy human plasma samples (N=13; black) and pooled human plasma samples (positive control (PC); blue) were used for each HDL-SPE assays performed using different lots of LS-CSH on different days. Experiment 1: batch #1 of LC-CSH on Day 0; Experiment 2: Batch #2 of LC-CSH, 4 months later. Left Panel: Mean of differences (raw data) is about -1.0. Right Panel: Normalization of the data with PC minimized the inter-experimental variation to nearly zero, with good reproducibility (<±10%). Paired t-test was used. *p<0.05 was considered statistically significant.

(C) LC-CSH temperature stability. LC-CSH was stored at 4 °C, -20 °C or -80 °C for 7 days and then thawed at 4 °C for 1 h prior to use.

(D) LC-CSH temperature stability over time. LC-CSH was stored at 4 °C for 0, 1, 3, or, 6 weeks, or at -20 °C for 6 weeks, prior to use in HDL-SPE assay. *p<0.05 vs. control (0 weeks). ****p<0.0001 vs. control (0 weeks). HDL-SPE (relative%) is the relative value based on using HDL-SPE in control batch #1 as the 100% baseline in **(A);** 4 °C as control in **(C)**; and 0 weeks as control in **(D)**. Ordinary One-way ANOVA analysis was done for each comparison. For temperature stability assays in **(C)** and **(D)**, four individual LC-CSH preparations were pooled and then aliquoted into four individual pooled samples. Each data point represents triplicate samples from a single pooled LC-CSH preparation.

Supplemental Figure 6. Five saline washes are sufficient for *PE fluorimetric and proteomic LC-CSH release studies.

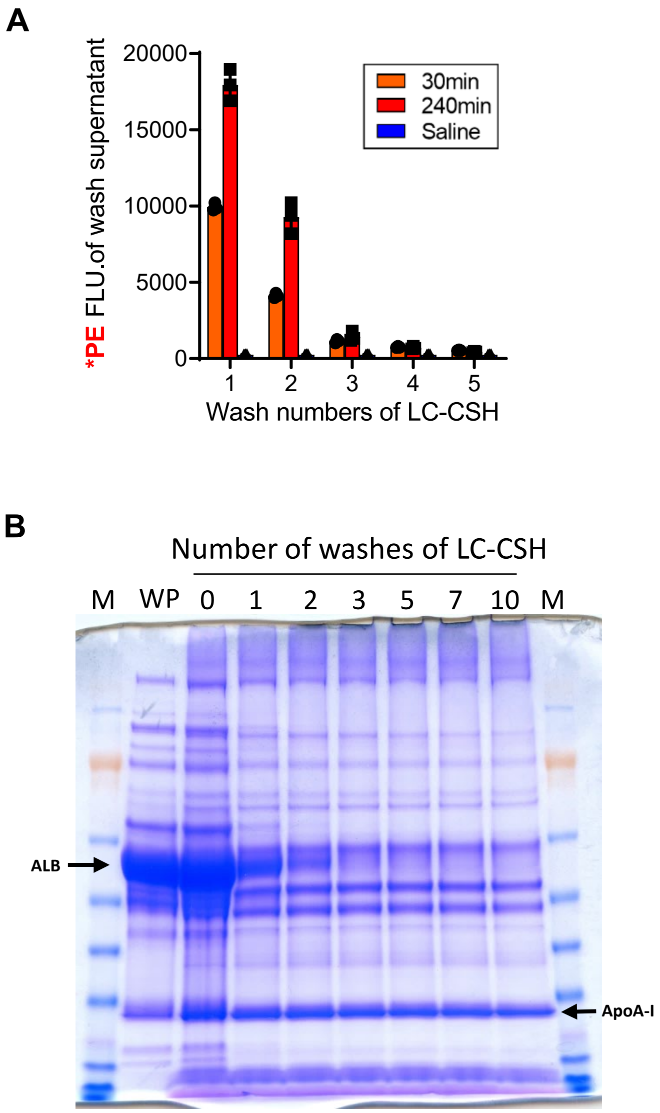

Supplemental Figure 6. Five saline washes are sufficient for *PE fluorimetric and proteomic LC-CSH release studies.

(A) *PE fluorescence in saline washes. Pooled human plasma or saline were incubated with LC-CSH under standard conditions for 30 or 240 min, supernatant was removed, and the LC-CSH were washed with saline from one to five times. Note that *PE fluorescence in the washes is nearly eliminated after five saline washes. **(B)** Assessment of protein content associated with LC-CSH after saline washes (SDS-PAGE gel stained with Coomassie blue). Proteins were extracted with SDS as described in Methods. Albumin and apoA-I were identified by in-gel proteomics (not shown). Note that the apoA-I band remained unaltered while the albumin and other protein bands were minimized after five or more saline washes. These findings indicate that five washes optimally remove contaminating supernatant proteins, thus allowing for assessment of LC-CSH bound and released proteins. Data are mean ± SD in triplicate assays.

Methods

Assessment of the efficacy of removal of unbound plasma proteins from LC-CSH by fluorimetry and SDS-PAGE electrophoresis. 25 µL of pooled human plasma, 50 µL of LC-CSH and, 75 µL of saline were incubated for 30 min at 37 ^{o}C with shaking at 1200 rpm. 120 µL of supernatant was removed and replaced with 120 µL of new cold saline. After pipetting vigorously 5 times, LC-CSH was precipitated by centrifugation (2000 rpm for 2 min), and 120 µL of the supernatant was removed and replaced again with of 120 µL of new cold saline. This washing process was repeated up to ten times as indicated. All washing procedures were performed at 4 ^{o}C. *PE fluorescence in the supernatant (*PE not bound to LC-CSH) was reduced to the same level as saline background level after five washes, even after LC-CSH was pre-incubated for 240 min with human plasma. To confirm the stability of plasma protein binding to LC-CSH after 30 min pre-incubation with pooled human plasma, samples were prepared for SDS-PAGE electrophoresis as follows: 50 µL of lysis buffer (2% SDS, 10 mM DTT in 50 mM triethylammonium bicarbonate buffer pH 8.5) was added to washed (1-10 times) and to unwashed LC-CSH as well as 1 µL of pooled human plasma, as reference controls. Samples were extracted and denatured for 5 min at 95 ^{o}C with shaking at 600 rpm in lysis buffer. 30 µL of the lysate was obtained from supernatant and incubated with 10 µL of 4X NuPAGE sample buffer (Invitrogen #NP0007) for 5min at 95 ^{o}C. 40 µL of each sample was loaded on to Novex NuPAGE 4-12% Bis-Tris Gel 1.5mm (Invitrogen #NP0335) and run with NuPAGE MOPS running buffer (Invitrogen #NP0001) at 200V for 50 min at room temperature. SeeBlue plus2 pre-stained standard (Invitrogen #LC5925) was used as a protein size marker.

Supplemental Figure 6. Five saline washes are sufficient for *PE fluorimetric and proteomic LC-CSH release studies.

Methods (continued)

After gel electrophoresis, protein bands were stained for 30 min with Coomassie Brilliant Blue (CBB: 1g of R-250, 400mL of methanol, 100 mL of acetic acid and 500 mL of double distilled water) and then de-stained overnight (200 mL of methanol, 100 mL of acetic acid and 700 mL of double distilled water). After five or more saline washes, apoA-I bands on the gel remained unaltered whereas albumin and other protein bands were reduced to a minimum compared to unwashed controls (Supplemental Figure 6B). ApoA-I and albumin were identified by in-gel proteomic analysis (not shown). These findings indicate that five washes optimally remove contaminating supernatant proteins and *PE, thus allowing for the proteomic analysis of LC-CSH-bound and released plasma proteins associated with*PE efflux (Figure 2).

In-gel proteomic analysis. Eluted plasma proteins that were bound to LC-CSH were subjected to SDS-PAGE, and gels were stained with CBB. Protein bands were excised, then destained, reduced, alkylated and digested overnight with trypsin (Promega, V511A Sequencing grade). Digested peptides were desalted, concentrated and purified using C18 ZipTips according to the manufacturer's protocol (ZTC18S096, Millipore Sigma) and transferred into sample vials (C4011-13, Thermo Scientific) for mass spectrometry.

Supplemental Figure 7. *PE is released as small HDL particles from LC-CSH.

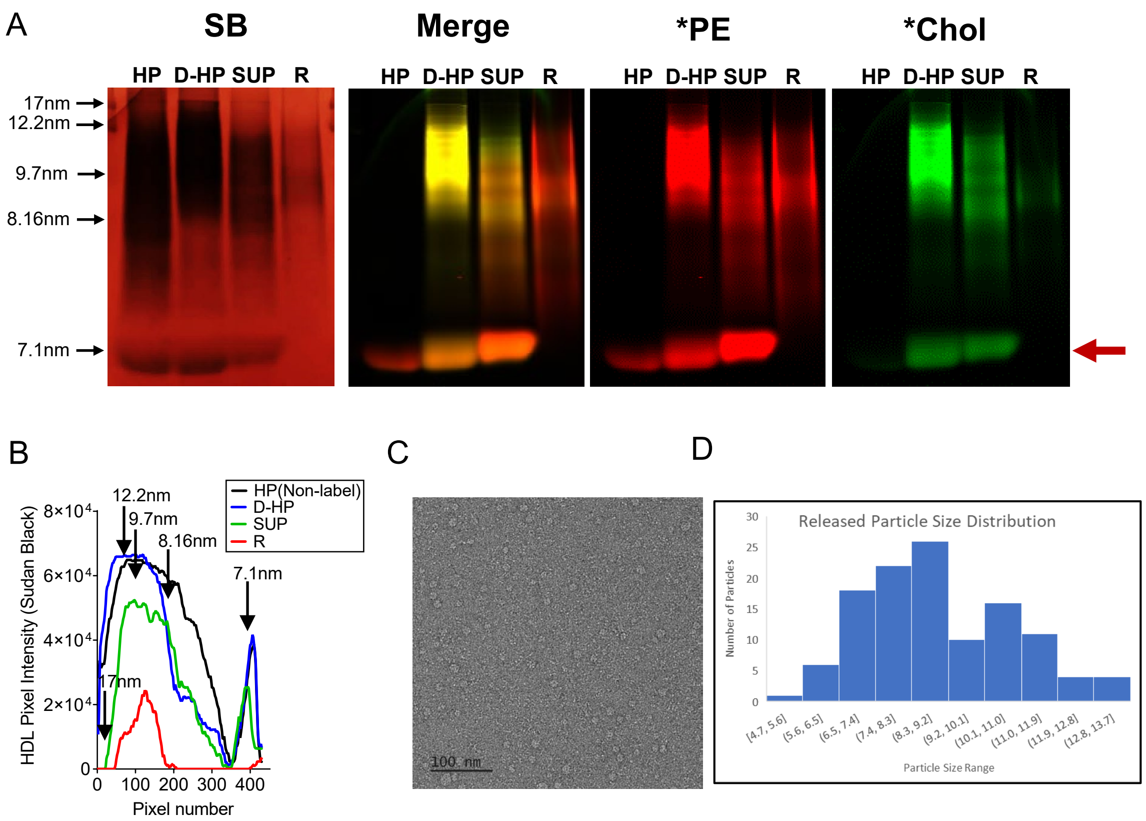

Electron microscopy. Negative staining. LC-CSH particles were diluted to 0.6 mg/ml with PBS sample buffer, whereas samples of particles released from LC-CSH (R) were not diluted, and 0.3 μl of sample solution was placed on each FCF300H-CU (Electron Microscopy Sciences PA, USA) grid and left at room temperature for 1 min. Grids were subsequently blotted using filter paper, washed with filtered distilled water three times and then stained with uranyl acetate 2% (Electron Microscopy Sciences PA, USA) for 20 seconds. Grids were then blotted with filter paper to remove excess stain and left to dry at room temperature for 15 min. Samples were evaluated by FEI Tecnai T12 120kV TEM & 2k TVIPS camera.

Supplemental Figure 7. *PE is released as small HDL particles from LC-CSH.

(A) Native gel analysis of HDL-SPE. Gel imaged for *PE and *Chol fluorescence, then HDL core hydrophobic lipids were stained with Sudan Black (SB). HP: unlabeled pooled human plasma; D-HP: HP directly labeled with *PE and *Chol, as described in Methods. SUP: HDL-SPE supernatant after incubation of HP with *PE-labeled LC-SCH for 30 min; R: HDL particles released to saline from LC-CSH pre-incubated with HP. Merge: Merged image of *PE and *Chol fluorescence imaging. Red arrow indicates small-HDL/albumin band. SB staining (i) reveals a shift in electrophoretic mobility for HDL in D-HP vs HP to the presence of fluorophores on D-HP, resulting in a larger "apparent size" for D-HP vs HP HDL particles; (ii) similar staining of the small HDL/albumin band in HP and D-HP and, (iii) reduced staining in SUP and little, if any, SB staining in R. Because the SUP and R samples were labeled with *PE by the same mechanism (LC-CSH-mediated efflux), their relative *PE fluorescence intensity can be compared. However, because direct labeling and LC-CSH *PE efflux-mediated labeling of HDL involve different mechanisms, only the relative particle size distribution of D-HP HDL, but not D-HP intensity, can be compared with the SUP and R samples. Fluorescent *PE and *Chol imaging demonstrates that: (i) the unlabeled HP small HDL/albumin band exhibits a small amount of red (*PE) autofluorescence, but no green (*Chol) autofluorescence and, (iii) this band in (R) contains no *PE or *Chol. Moreover, the (R) lane contains little *Chol. Thus, the SUP small HDL/albumin band is markedly enriched with *PE compared to SB. Note the near lack of *Chol in the R lane, but a substantial amount in SUP, consistent with predominantly apolipoprotein-mediated solubilization and release of LC-CSH *PE in R vs desorption and diffusion of LC-CSH *Chol to plasma HDL species in SUP. **(B)** Kymograph analysis of Sudan Black stain in **(A)** shows that D-HP particle size shifts to larger size compared to non-labeled HP baseline, reduced staining in the SUP lane and small HDL/albumin band, and markedly reduced staining in R compared to SUP. **(C)** Negatively stained lipoproteins released from LC-CSH particles into saline (R) imaged by transmission electron microscopy. Scale bar = 100nm. **(D)** Size distribution of lipoprotein particles in **(C)**. Note the median particle diameter = 8.8 nm. Geometric diameter of particles was measured in > 100 particles in two electron micrographs at the same magnification using image J software.

Supplemental Figure 8. Non-apolipoprotein-mediated HDL-SPE.

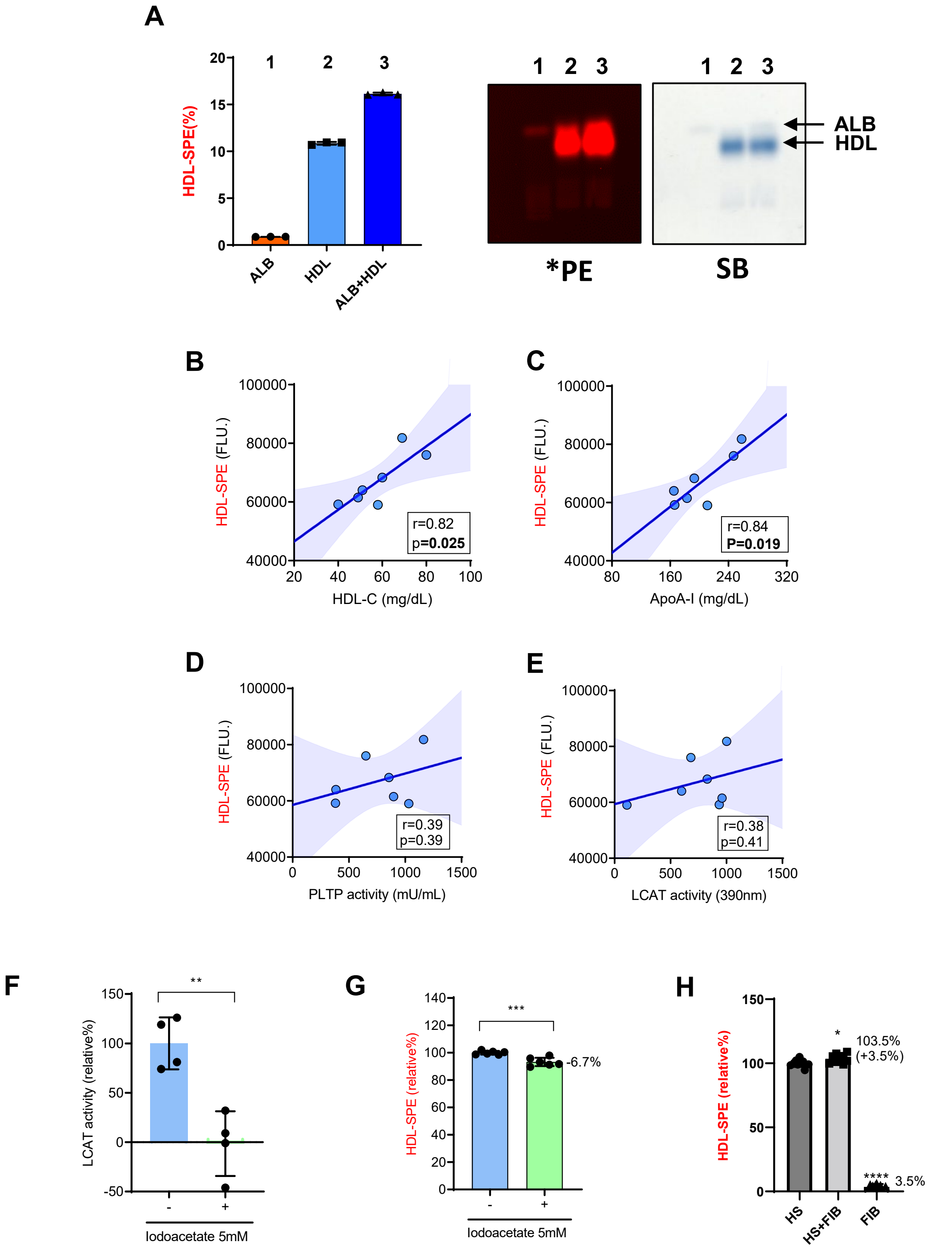

Supplemental Figure 8. Non-apolipoprotein-mediated HDL-SPE.

Albumin modulates HDL-SPE. (A) Albumin-mediated HDL-SPE. Agarose gel *PE and SB analysis. Albumin (ALB) mediates little HDL-SPE itself, but markedly increases HDL-mediated HDL-SPE. Thus, although albumin effluxes little *PE from LC-CSH, ALB markedly increases HDL-mediated efflux. Thus, ALB facilitates HDL-SPE. 25 µL of 40 µg/µL ALB (total 1000 µg) and/or 25 µL of 80 mg/dL isolated HDL (total 20 µg of phospholipid). Albumin and phospholipid content represent human physiological concentrations. HDL-SPE data represent a representative experiment performed in triplicate assay (from two independent experiments).

Neither PLTP nor LCAT activity significantly correlates with HDL-SPE. Correlation analysis was performed between serum HDL-SPE and **(B)** HDL-C, **(C)** apoA-I, **(D)** PLTP activity and, **(E)** LCAT activity (n=7, triplicate samples). Note that although both HDL-C and apoA-I were highly correlated with HDL-SPE, neither LCAT nor PLTP activity correlated with HDL-SPE. PLTP activity was measured based on increase in 535 nm FLU between 8 and 20 min at 37°C and calculated using the conversion factor provided by the manufacturer (concentration of donor particle), as described in Methods section. LCAT activity was measured based on increasement of 390 nm FLU between 15 min and 2.5 h at 37 °C, as described in Methods section. For correlation analysis, Pearson r was evaluated and $p<0.05$ was considered statistically significant.

LCAT does not play an important role in HDL-SPE. (F) Complete inhibition of LCAT activity by 5 mM Iodoacetate was confirmed using human pooled plasma (HP). LCAT activity was measured based on increase of 390 nm FLU between 15 min and 2.5 h at 37 °C with or without 5 mM Iodoacetate of LCAT inhibitor, as described in Methods section. **(G)** HDL-SPE was performed at 37°C for 1 hour with or without 5 mM Iodoacetate. LCAT activity relative% in **(F)** and HDL-SPE relative% in **(G)** is the relative value based on using LCAT activity or HDL-SPE in control HP without LCAT inhibitor as the 100% baseline. Each point represents the mean of duplicate assay in two independent experiments (n=4) in **(F)** and the mean of triplicate assay in two independent experiments (n=6) in **(G)**. Statistical analysis was done by unpaired t-test. $p<0.05$ was considered statistically significant. **$p<0.01$

Human fibrinogen has a negligible effect on plasma HDL-SPE. (H) The effect of the presence of human purified fibrinogen (FIB) on HDL-SPE was evaluated using human pooled serum (HS). HDL-SPE was performed using HS spiked with 2 mg/mL FIB (physiological concentration in human plasma) or with 2 mg/mL FIB alone in PBS. HDL-SPE relative% is the relative value based on using HDL-SPE in control HS as the 100% baseline. The data represent the mean of triplicate assays in three independent experiments (n=9). Statistical analysis was performed by one-way ANOVA followed by multiple comparison test (d). $p<0.05$ was considered statistically significant. *$P<0.05$ vs. HS. ****$p<0.0001$ vs. HS.

Supplemental Figure 8. Non-apolipoprotein-mediated HDL-SPE.

Methods

PLTP activity assay. PLTP assay was performed using PLTP Activity Assay Kit (Catalog Number MAK108, Supplied by Roar Biomedical, Inc, via SIGMA-ALDRICH) according to manufacturer instructions. Briefly, PLTP activity was measured based on increase in 535 nm FLU. between 8 and 20 min at 37 °C and calculated using the conversion factor (concentration of donor particle: 322 nmol per mL), provided by the manufacturer. PLTP assay was performed in duplicate.

LCAT activity assay. LCAT assay was performed using LCAT Activity Assay Kit (Catalog Number MAK107, Supplied by Roar Biomedical, Inc, via SIGMA-ALDRICH) according to manufacturer instructions. Briefly, LCAT activity was measured based on increasement of 390 nm FLU between 15 min and 2.5 h at 37 °C. LCAT assay was performed in duplicate.

Supplemental Figure 9. HDL-SPE correlates with CEC and HDL parameters in Clinical Study I.

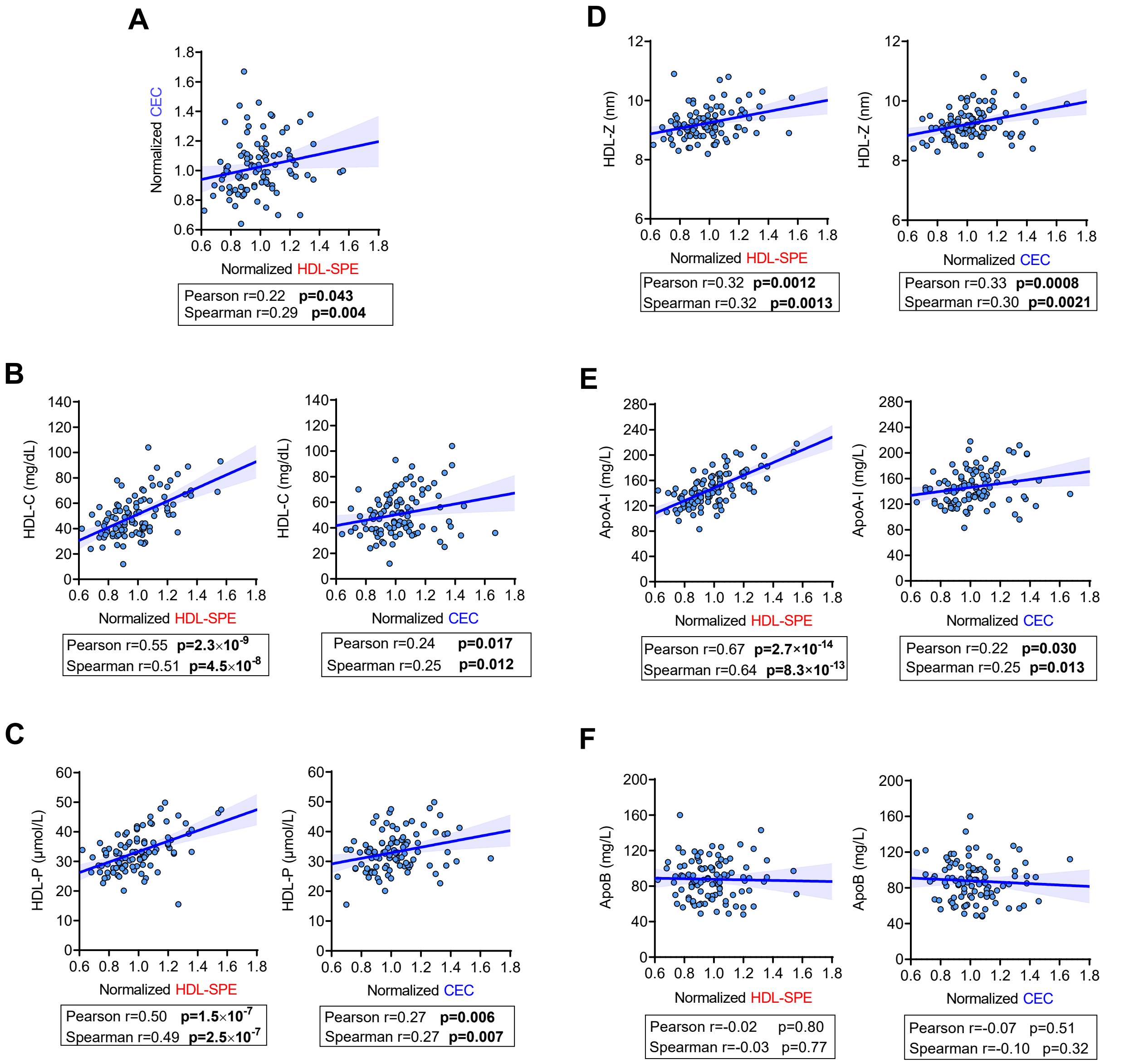

(A) Correlation between HDL-SPE and CEC (J774 cells) assays in cohort of 100 CVD patients including 50 severe stenosis/obstructive CAD subjects and 50 non-CAD/non-obstructive CAD subjects. HDL-SPE weakly correlates with CEC. Correlation of plasma levels of HDL cholesterol (HDL-C) **(B)**, HDL particle number (HDL-P) **(C)**, HDL particle size (HDL-Z) **(D)**, apoA-I **(E)**, and apoB **(F)**, with HDL-SPE and CEC. HDL-SPE and CEC both correlate with plasma HDL-C, HDL-P, HDL-Z and apoA-I, but not apoB. Note, of all the HDL-related parameters, the highest correlation is between HDL-SPE and plasma apoA-I levels, consistent with the apoA-I dependent-mechanism of HDL-SPE demonstrated in Figure 1 and Figure 2 in the manuscript.

Supplemental Figure 10. HDL-SPE inversely correlates with total and non-calcified plaque burden in CAD subjects assessed by coronary CT angiography.

HDL-SPE inversely correlates non-calcified coronary artery plaque burden. We compared the ability of the HDL-SPE and CEC assays to predict coronary artery plaque burden in 208 arteries from a cohort of 73 known cardiovascular disease (CVD) subjects assessed by coronary CT angiography (CCTA). See Supplemental Table 4 "Clinical characteristics of clinical study Ib subjects." This cohort is 50.7% male with an average age of 60.2 yrs and is generally a normolipidemic cohort. For Clinical Study Ib, the sera used in the US cohort study included 84 cardiovascular disease (CVD) patients, recruited as part of an ongoing cohort study as previously reported (1). Eleven patients were excluded in this study due to missing data or insufficient volume for HDL-SPE assay.

CEC has previously been shown to be inversely correlated with atherosclerotic non-calcified plaque burden in psoriasis patients (2). Multivariate linear regression analysis revealed that after adjustment for traditional CVD risk factors including age, gender, BMI, systolic BP, LDL-C, TG, hs-CRP, lipid-lowering therapy, current smoker, DM2, race, PCI/CABG and HDL-C, HDL-SPE significantly inversely correlated with NCB, TB, FFB and FB, whereas in this cohort, CEC did not.

NCB, specifically NB and FFB, represent the lipid-rich component of vulnerable atherosclerotic plaque (3,4). Thus, the inverse correlation of HDL-SPE with NCB may reflect the ability of apoA-I and other exchangeable apolipoproteins to remove arterial plaque lipids in vivo. The potential importance of our findings are underscored by several recent studies that provide evidence that NCB may be a better predictor of ACS and MACE risk than traditional risk factors (3).

Supplemental Figure 10. HDL-SPE inversely correlates with non-calcified plaque burden in CAD subjects assessed by coronary CT angiography.

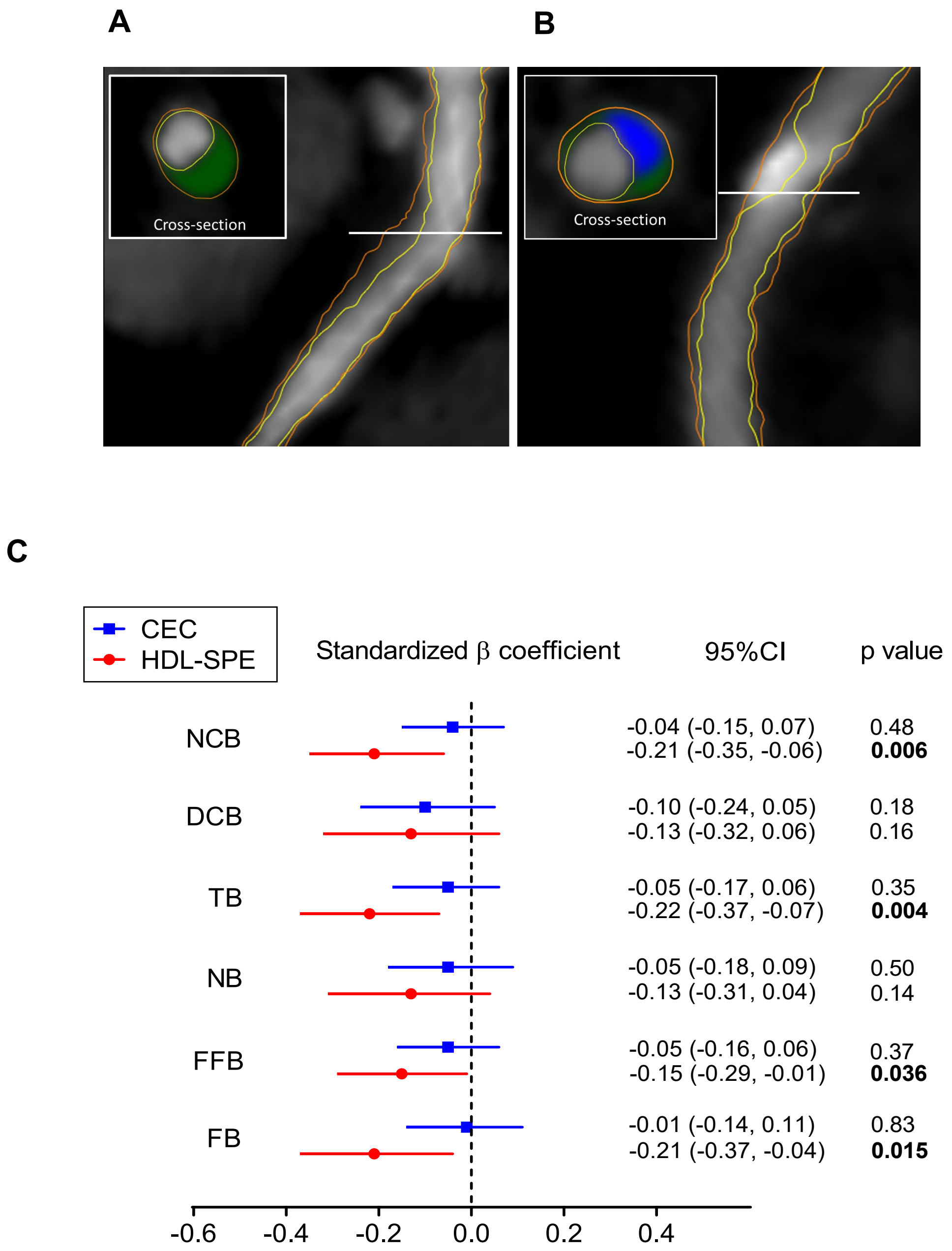

Adjusted for age, gender, BMI, systolic BP, LDL-C, LLT, TG, hs-CRP, DM2, Current SMK, race, PCI/CABG and HDL-C

Supplemental Figure 10. HDL-SPE inversely correlates with non-calcified plaque burden in CAD subjects assessed by coronary CT angiography.

(A,B) Representative CT angiograms. (A) Non-calcified plaque (NCB) in the proximal left anterior descending artery shown in curved multiplanar reconstruction and cross-section taken at the white line. Widened region between lumen boundary and vessel wall contains abundant NCB.
(B) Mixed calcified plaque (DCB) and NCB in the left circumflex artery shown in curved multiplanar reconstruction and cross-section taken at the white line. Yellow: Lumen boundary. Orange: Vessel wall. Green: NCB. Blue: DCB.

(C) Multivariate linear regression analysis of HDL-SPE and CEC association with CCTA plaque burden (n=208 data). Multivariate linear regression analysis of HDL-SPE and CEC after adjustment for traditional risk factors and HDL-C. HDL-SPE, but not CEC, was significantly inversely correlated with NCB, TB, FFB and FB. $p<0.05$ was considered statistically significant. Non-parametric variables were log transformed before analysis. HDL-C: high density lipoprotein cholesterol, CEC: cholesterol efflux capacity, HDL-SPE: HDL-specific phospholipid efflux. NCB: non-calcified plaque burden , DCB: dense calcium burden, TB: total plaque burden, NB: necrotic burden, FFB: fibro-fatty burden, FB: fibrous burden. Adjusted variables included age, gender, BMI: Body mass index, SysBP: systolic blood pressure, LDL-C: low density lipoprotein cholesterol, LLT: lipid lowering therapy, TG: triglyceride, hs-CRP: high sensitivity C-reactive protein, DM2: type2-diabetes mellitus, Current SMK: current smoking, race, PCI/CABG: percutaneous coronary intervention or coronary artery bypass grafting, and HDL-C: high density lipoprotein cholesterol.

Supplemental Figure 10. HDL-SPE inversely correlates with non-calcified plaque burden in CAD subjects assessed by coronary CT angiography.

METHODS:

Atherosclerosis burden assessment. Coronary CT scans were performed using a 320-detector row Aquilion ONE ViSION system (Toshiba). Coronary plaque burden was separately evaluated in each of the main coronary arteries (left anterior descending, left circumflex, and right coronary arteries) using the dedicated software QAngio CT (Medis) by a single blinded reader as previously reported (2). Plaque volume index (mm^2) was calculated by dividing total vessel plaque volume by total vessel length and was adjusted for luminal attenuation. Total plaque burden (TB) was defined as the sum of calcified plaque burden (DCB) and non-calcified plaque burden (NCB) (1). Non-calcified plaque subcomponents including fibrous (FB), fibro-fatty (FFB), and necrotic (NB) burdens were obtained after adaptively correcting for lumen attenuation and depicted based on Hounsfield Units.

REFERENCES:

1. Gordon, S. M., et al. (2018). "High density lipoprotein proteome is associated with cardiovascular risk factors and atherosclerosis burden as evaluated by coronary CT angiography." Atherosclerosis 278: 278-285.

2. Salahuddin, T., et al. (2015). "Cholesterol efflux capacity in humans with psoriasis is inversely related to non-calcified burden of coronary atherosclerosis." Eur Heart J 36(39): 2662-2665.

3. Voit, J. and K. R. H. Branch (2022). "Coronary plaque in the fourth dimension: associating coronary computed tomography plaque components and the time to acute coronary syndrome " European Heart Journal - Cardiovascular Imaging 23(10): 1324-1325.

4. van den Hoogen IJ, Stuijfzand WJ, Gianni U, van Rosendael AR, Bax AM, ET AL.. Early versus late acute coronary syndrome risk patterns of coronary atherosclerotic plaque. Eur Heart J Cardiovasc Imaging. 2022 Sep 10;23(10):1314-1323. doi: 10.1093/ehjci/jeac114. PMID: 35904766.

Supplemental Figure 11. HDL-SPE correlates with HDL-C and apoA-I parameters in Clinical Study II.

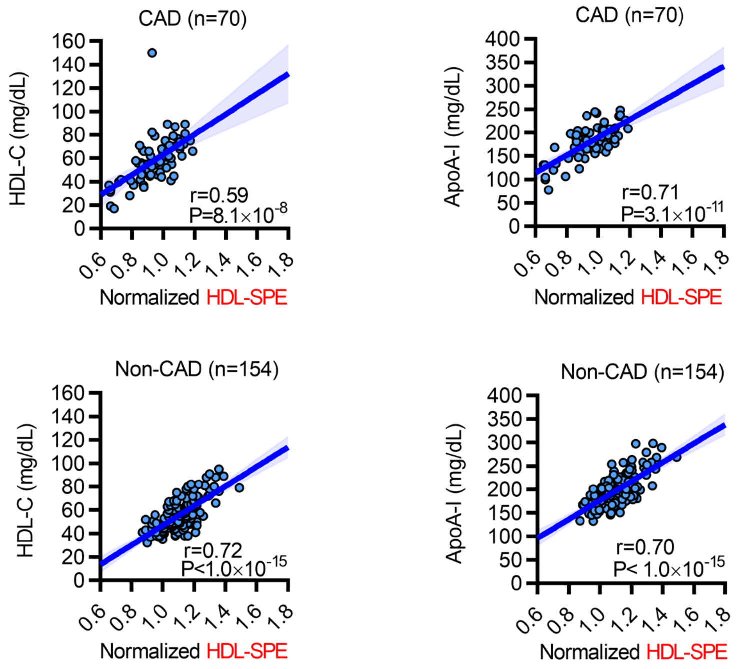

HDL-C and ApoA-I correlate with HDL-SPE in Japanese cohort of CAD- (n=154) and non-CAD (n=70) subjects. Pearson's correlation analysis was performed and $p<0.05$ was considered statistically significant.

Supplemental Figure 12. ApoA-I, but not HDL-C, correlated linearly with HDL-SPE in subjects with HDL-C ≥ 80 mg/dL in Clinical Study II (Japanese cohort).

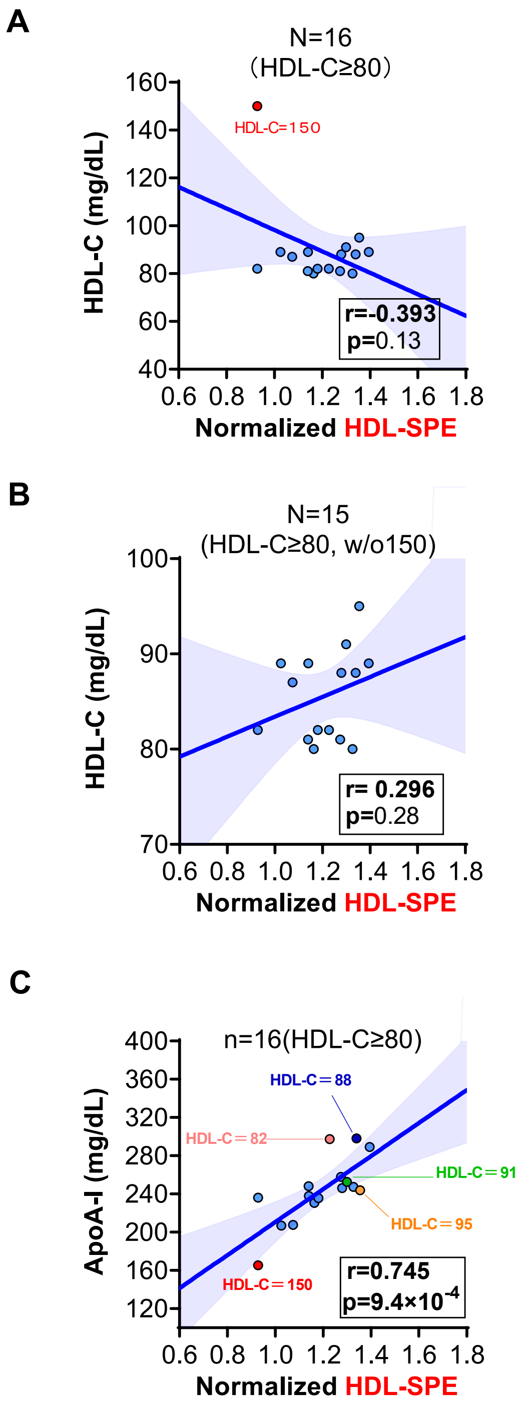

Supplemental Figure 12. ApoA-I, but not HDL-C, correlated linearly with HDL-SPE in subjects with HDL-C ≥ 80 mg/dL in Clinical Study II (Japanese cohort).

Analysis of HDL-SPE in subjects with HDL-C ≥ 80 mg/dL in Clinical Study II (Japanese cohort).

Correlation analysis in subjects with HDL-C ≥ 80 mg/dL was performed between HDL-SPE and:

(a) HDL-C (N=16 subjects with HDL-C ≥ 80 mg/dL).

(b) HDL-C (N=15 subjects excluding HDL-C = 150 mg/dL).

(c) ApoA-I (N=16 subjects with HDL-C ≥ 80 mg/dL). A

These analysis of HDL-SPE in subjects with HDL-C ≥ 80 mg/dL in Clinical Study II (Japanese cohort), albeit using a limited sample size (N = 16), shows that apoA-I, but not HDL-C, correlated linearly with HDL-SPE.

These findings suggest that our HDL-SPE assay may still be quantitative in samples with high HDL-C, and that HDL-SPE can detect dysfunctional HDL.

Supplemental Table 1. Ranked plasma proteins identified and quantified by LC MS/MS

	Gene Symbols	UniProt protein ID	iBAQ AV (n=3)	iBAQ SD (n=3)
**1**	***ALB***	P02768	1.57E+10	4.06E+08
**2**	***IGHG1***	P01857	3.39E+09	2.86E+08
**3**	***APOA1***	P02647	1.85E+09	4.38E+08
**4**	***HP***	P00738	1.30E+09	1.98E+08
**5**	***IGHA1***	P01876	1.23E+09	2.47E+08
**6**	***TF***	P02787	1.10E+09	2.17E+08
**7**	***IGHG2***	P01859	8.63E+08	7.91E+07
**8**	***SERPINA1***	P01009	7.61E+08	1.11E+08
**9**	***IGHG3***	P01860	5.62E+08	1.40E+08
**10**	***A2M***	P01023	4.66E+08	1.89E+07
**11**	***IGK***	P0DOX7	4.61E+08	4.65E+07
**12**	***FGA***	P02671	4.46E+08	1.05E+08
**13**	***IGHM***	P01871	4.45E+08	4.09E+07
**14**	***FGG***	P02679	3.82E+08	4.08E+07
**15**	***IGLL5*; *IGLC1***	B9A064; P0CG04	3.52E+08	3.21E+08
**16**	***FGB***	P02675	2.86E+08	7.11E+06
**17**	***KV315***	P01624	2.75E+08	3.46E+07
**18**	***C3***	P01024	2.74E+08	3.11E+07
**19**	***TTR***	P02766	2.59E+08	2.04E+07
**20**	***SERPINA3***	P01011	2.31E+08	4.70E+07
21	*APOA2*	P02652	1.86E+08	7.21E+06
22	*KV320*	P01619	1.85E+08	2.70E+07
23	*HPX*	P02790	1.47E+08	1.63E+07
24	*APOC1*	P02654	1.46E+08	6.47E+06
25	*IGKVA18*; *IGKV2D-26*; *IGKV2D-29*	A2NJV5; A0A0A0MRZ7; A0A075B6S2	1.38E+08	1.18E+08
26	*SERPINC1*	P01008	1.20E+08	8.71E+06
27	*APOH*	P02749	1.13E+08	2.26E+07
28	*HBB*	P68871	1.11E+08	3.59E+07
29	*HBA1*	P69905	1.09E+08	1.07E+07
30	*IGHV3-15*	A0A0B4J1V0	1.08E+08	1.92E+07
31	*AHSG*	P02765	1.03E+08	1.23E+07
32	*VTN*	P04004	9.15E+07	8.34E+06

Supplemental Table 1. Ranked plasma proteins identified and quantified by LC MS/MS

33	*SERPING1*	P05155	8.91E+07	2.94E+07
34	*PLG*	P00747	8.73E+07	6.22E+06
35	*LV147*	P01700	8.32E+07	6.38E+06
36	*F2*	P00734	7.99E+07	5.25E+06
37	*CFB*	P00751	7.41E+07	1.45E+07
38	*A1BG*	P04217	7.22E+07	1.24E+07
39	*C4B*	P0C0L5	7.15E+07	1.43E+07
40	*IGKV4-1*	P06312	7.09E+07	6.23E+06
41	*AMBP*	P02760	7.01E+07	1.79E+07
42	*CLU*	P10909	6.55E+07	1.18E+07
43	*IGJ*	P01591	6.48E+07	8.34E+06
44	*ORM2*	P19652	6.33E+07	1.50E+07
45	*GC*	P02774	6.14E+07	9.21E+06
46	*APOA4*	P06727	6.11E+07	1.79E+07
47	*IGHV4-61*; *IGHV4-4*	A0A0C4DH41; A0A075B6R2	5.84E+07	4.57E+07
48	*ORM1*	P02763	5.83E+07	2.90E+07
49	*CP*	P00450	5.66E+07	5.59E+06
50	*CFH*	P08603	5.62E+07	6.46E+06
51	*IGA2*	P0DOX2	5.44E+07	3.39E+06
52	*C4BPA*	P04003	5.40E+07	2.70E+06
53	*APOE*	P02649	5.14E+07	8.60E+06
54	*KRT1*	P04264	4.53E+07	3.29E+07
55	*IGHV1-18*	A0A0C4DH31	4.35E+07	2.37E+06
56	*APOC3*	P02656	4.34E+07	1.18E+07
57	*IGHG4*	P01861	4.24E+07	3.72E+07
58	*APOB*	P04114	4.10E+07	1.65E+06
59	*IGLV7-46*	A0A075B6I9	4.02E+07	1.11E+07
60	*IGLV3-10*	A0A075B6K4	3.85E+07	1.10E+07
61	*APOM*	O95445	3.84E+07	2.55E+06
62	*SERPINF2*	P08697	3.54E+07	8.45E+06
63	*AGT*	P01019	3.39E+07	1.68E+07
64	*IGHV3-23*	P01764	3.34E+07	7.30E+06
65	*SAA4*	P35542	3.33E+07	5.97E+06
66	*LV319*	P01714	3.27E+07	1.34E+07
67	*IGHV1-3*; *IGHV1-69-2*	A0A0C4DH29; A0A0B4J2H0	3.23E+07	3.06E+06

Supplemental Table 1. Ranked plasma proteins identified and quantified by LC MS/MS

68	*IGKV2-24*	A0A0C4DH68	3.17E+07	2.85E+06
69	*LV321*	P80748	3.04E+07	1.76E+06
70	*CRP*	P02741	3.04E+07	4.19E+06
71	*ITIH4*	Q14624	2.78E+07	4.68E+06
72	*KRT10*	P13645	2.55E+07	2.24E+07
73	*CD5L*	O43866	2.54E+07	4.67E+06
74	*LV214*	P01704	2.47E+07	4.05E+06
75	*GSN*	P06396	2.46E+07	2.00E+06
76	*ITIH1*	P19827	2.38E+07	6.58E+06
77	*IGHV3-74*; *IGHV3-66*	A0A0B4J1X5; A0A0C4DH42	2.34E+07	6.54E+06
78	*KVD39*	P04432	2.32E+07	5.41E+06
79	*SERPIND1*	P05546	2.24E+07	2.15E+06
80	*LRG1*	P02750	2.12E+07	4.35E+06
81	*AZGP1*	P25311	1.95E+07	2.47E+06
82	*IGHV5-51*	A0A0C4DH38	1.92E+07	5.78E+06
83	*C1QB*	P02746	1.91E+07	8.88E+06
84	*IGKV3D-15*	A0A087WSY6	1.86E+07	2.24E+06
85	*PON1*	P27169	1.81E+07	1.23E+06
86	*SERPINF1*	P36955	1.80E+07	4.48E+06
87	*KNG1*	P01042	1.77E+07	9.66E+05
88	*RBP4*	P02753	1.69E+07	7.28E+06
89	*KRT9*	P35527	1.66E+07	2.00E+07
90	*ITIH2*	P19823	1.58E+07	3.16E+06
91	*C8G*	P07360	1.51E+07	6.56E+06
92	*C9*	P02748	1.47E+07	4.22E+06
93	*AFM*	P43652	1.27E+07	1.53E+06
94	*IGHD*	P0DOX3	1.22E+07	3.39E+06
95	*C5*	P01031	1.22E+07	3.07E+06
96	*KRT2*	P35908	1.14E+07	9.37E+06
97	*HRG*	P04196	1.13E+07	1.63E+06
98	*IGHV3-49*	A0A0A0MS15	1.10E+07	7.35E+06
99	*KRT16*	P08779	1.08E+07	5.63E+06
100	*CFI*	P05156	1.05E+07	1.60E+06
101	*APCS*	P02743	1.04E+07	1.26E+06
102	*PROS1*	P07225	1.04E+07	2.45E+06

Supplemental Table 1. Ranked plasma proteins identified and quantified by LC MS/MS

103	*KLKB1*	P03952	1.01E+07	2.25E+06
104	*FCN3*	O75636	1.00E+07	1.53E+06
105	*APOL1*	O14791	9.85E+06	3.04E+05
106	*PF4*; *PF4V1*	P02776; P10720	9.72E+06	2.61E+06
107	*PGLYRP2*	Q96PD5	9.60E+06	3.24E+06
108	*IGHV3*	A0A0J9YX35	9.27E+06	4.27E+06
109	*IGKV1-8*; *IGKV1-9*	A0A0C4DH67; A0A0C4DH69	9.08E+06	4.00E+06
110	*SERPINA6*	P08185	8.89E+06	1.13E+06
111	*C4BPB*	P20851	8.83E+06	8.86E+05
112	*C8A*	P07357	8.38E+06	3.63E+06
113	*F12*	P00748	8.34E+06	1.39E+06
114	*FN1*	P02751	8.23E+06	1.10E+06
115	*C8B*	P07358	6.27E+06	5.31E+05
116	*C1R*	P00736	6.20E+06	6.11E+05
117	*C7*	P10643	5.74E+06	7.62E+05
118	*HPR*	P00739	5.34E+06	8.52E+05
119	*ITIH3*	Q06033	4.97E+06	5.25E+05
120	*ACTB*; *ACTG1*	P60709; P63261	4.45E+06	1.72E+06
121	*IGHV3-43*	A0A0B4J1X8	4.39E+06	5.63E+05
122	*C6*	P13671	4.00E+06	1.19E+06
123	*CLEC3B*	P05452	3.96E+06	7.67E+05
124	*KRT5*	P13647	3.84E+06	5.16E+06
125	*CPN2*	P22792	3.81E+06	1.91E+06
126	*S100A8*	P05109	3.26E+06	1.63E+06
127	*APOD*	P05090	3.22E+06	3.08E+06
128	*C1S*	P09871	2.87E+06	4.76E+05
129	*IGFALS*	P35858	2.49E+06	5.70E+05
130	*C2*	P06681	2.47E+06	1.07E+05
131	*CFHR5*	Q9BXR6	2.40E+06	2.00E+05
132	*SEPP1*	P49908	2.39E+06	6.87E+05
133	*LUM*	P51884	2.35E+06	1.33E+06
134	*LBP*	P18428	2.10E+06	1.36E+06
135	*IGFBP3*	P17936	2.05E+06	4.95E+05
136	*CD14*	P08571	1.99E+06	1.49E+06
137	*SERPINA4*	P29622	1.95E+06	1.05E+06

Supplemental Table 1. Ranked plasma proteins identified and quantified by LC MS/MS

138	*SERPINA7*	P05543	1.91E+06	4.42E+05
139	*ZGRF1*	Q86YA3	1.89E+06	7.89E+05
140	*GPLD1*	P80108	1.82E+06	3.97E+05
141	*CPN1*	P15169	1.76E+06	8.25E+05
142	*F9*	P00740	1.76E+06	2.44E+05
143	*FBLN1*	P23142	1.62E+06	5.14E+05
144	*CPB2*	Q96IY4	1.59E+06	3.82E+05
145	*ACTG2*; *ACTA1*; *ACTC1*; *ACTA2*	P63267; P68133; P68032; P62736	1.58E+06	4.58E+05
146	*F13B*	P05160	1.57E+06	7.96E+05
147	*F10*	P00742	1.31E+06	6.59E+05
148	*LGALS3BP*	Q08380	1.18E+06	1.31E+06
149	*HABP2*	Q14520	9.26E+05	3.13E+05
150	*MBL2*	P11226	8.29E+05	1.64E+05
151	*CFP*	P27918	7.06E+05	5.03E+05
152	*MASP1*	P48740	6.91E+05	2.45E+05
153	*BTD*	P43251	5.32E+05	3.21E+05
154	*LPA*	P08519	3.53E+05	1.78E+05
155	*ATRN*	O75882	3.38E+05	3.19E+04
156	*F5*	P12259	2.97E+05	6.80E+04
157	*THBS1*	P07996	2.41E+05	1.24E+05
158	*VWF*	P04275	7.94E+04	1.89E+04
159	*FCGBP*	Q9Y6R7	3.72E+04	2.32E+04

Supplemental Table 2. Ranked bound proteins identified and quantified by LC MS/MS

	Gene Symbols	UniProt protein ID	iBAQ AV (n=3)	iBAQ SD (n=3)
**1**	***APOA1***	P02647	7.20E+09	5.48E+08
**2**	***FGG***	P02679	4.25E+09	4.04E+08
**3**	***APOC1***	P02654	3.65E+09	9.37E+08
**4**	***FGA***	P02671	3.46E+09	6.18E+08
**5**	***FGB***	P02675	2.56E+09	2.52E+08
**6**	***APOE***	P02649	9.66E+08	1.70E+08
**7**	***APOA2***	P02652	8.87E+08	7.49E+08
**8**	***SAA4***	P35542	7.70E+08	1.04E+08
**9**	***SAA1***	P0DJI8	5.13E+08	4.98E+07
**10**	***APOH***	P02749	4.53E+08	2.04E+08
**11**	***APOC3***	P02656	4.42E+08	1.40E+08
**12**	***CFB***	P00751	3.75E+08	3.74E+07
**13**	***ALB***	P02768	3.71E+08	6.84E+07
**14**	***CLU***	P10909	3.20E+08	6.15E+07
**15**	***APCS***	P02743	3.09E+08	7.90E+07
**16**	***CLEC3B***	P05452	3.09E+08	1.67E+08
**17**	***PPBP***	P02775	3.03E+08	1.30E+08
**18**	***CFH***	P08603	2.61E+08	6.12E+07
**19**	***C3***	P01024	2.54E+08	3.37E+07
**20**	***SERPINF1***	P36955	2.17E+08	6.70E+07
21	*VTN*	P04004	2.16E+08	5.12E+07
22	*GSN*	P06396	2.06E+08	4.02E+07
23	*IGHG1*	P0DOX5	1.99E+08	7.82E+07
24	*F12*	P00748	1.84E+08	1.94E+07
25	*HABP2*	Q14520	1.81E+08	2.10E+07
26	*PLG*	P00747	1.77E+08	2.88E+07
27	*ITIH4*	Q14624	1.63E+08	2.03E+07
28	*PON1*	P27169	1.35E+08	3.01E+07
29	*SERPING1*	P05155	1.23E+08	1.80E+07
30	*AHSG*	P02765	1.16E+08	1.82E+07
31	*APOA4*	P06727	1.13E+08	2.48E+07
32	*KNG1*	P01042	1.12E+08	4.02E+07
33	*HRG*	P04196	1.10E+08	2.83E+07
34	*CFHR5*	Q9BXR6	8.76E+07	1.15E+07

Supplemental Table 2. Ranked bound proteins identified and quantified by LC MS/MS

35	*CST3*	P01034	8.71E+07	1.45E+07
36	*IGHA1*	P01876	7.71E+07	1.13E+07
37	*APOM*	O95445	7.52E+07	2.11E+07
38	*F2*	P00734	7.38E+07	1.94E+07
39	*IGHM*	P01871	7.18E+07	1.16E+07
40	*APOC2*	P02655	7.06E+07	4.61E+07
41	*CRP*	P02741	6.99E+07	1.72E+07
42	*IGLL5*; *IGLC1*	B9A064; P0CG04	6.69E+07	5.33E+07
43	*DEFA3*; *DEFA1*	P59666; P59665	6.14E+07	4.08E+07
44	*APOB*	P04114	6.13E+07	5.47E+06
45	*APOC4*	P55056	6.13E+07	3.01E+07
46	*HP*	P00738	6.01E+07	2.65E+07
47	*SEPP1*	P49908	5.82E+07	1.65E+07
48	*C1QB*	P02746	5.29E+07	1.23E+07
49	*IGHG3*	P01860	5.24E+07	5.81E+06
50	*GC*	P02774	5.23E+07	3.13E+06
51	*C8G*	P07360	4.58E+07	9.97E+06
52	*SERPINA5*	P05154	4.40E+07	6.19E+06
53	*C9*	P02748	4.37E+07	6.74E+06
54	*SERPINA1*	P01009	4.28E+07	5.50E+06
55	*C1QC*	P02747	4.27E+07	5.22E+06
56	*IGK*	P0DOX7	4.19E+07	3.52E+06
57	*C4BPA*	P04003	4.13E+07	6.04E+06
58	*APOD*	P05090	3.99E+07	9.00E+06
59	*C1S*	P09871	3.86E+07	8.01E+06
60	*C8A*	P07357	3.45E+07	1.44E+07
61	*IGHG2*	P01859	3.43E+07	8.26E+06
62	*C4B*	P0C0L5	3.34E+07	2.12E+06
63	*KRT1*	P04264	3.31E+07	1.94E+07
64	*CFD*	P00746	3.26E+07	6.48E+06
65	*C1R*	P00736	3.22E+07	7.56E+06
66	*C6*	P13671	3.15E+07	7.31E+05
67	*SERPINC1*	P01008	3.13E+07	1.16E+07
68	*AMBP*	P02760	3.03E+07	9.14E+06
69	*LBP*	P18428	3.02E+07	2.01E+07

Supplemental Table 2. Ranked bound proteins identified and quantified by LC MS/MS

70	*CFHR1*	Q03591	2.73E+07	4.26E+06
71	*C7*	P10643	2.48E+07	3.74E+06
72	*FN1*	P02751	2.44E+07	2.61E+06
73	*TTR*	P02766	2.37E+07	5.72E+06
74	*IGFBP2*	P18065	2.33E+07	4.95E+06
75	*KV320*	P01619	1.88E+07	8.70E+06
76	*KLKB1*	P03952	1.86E+07	9.93E+05
77	*F10*	P00742	1.75E+07	4.69E+06
78	*A2M*	P01023	1.65E+07	2.42E+06
79	*PCOLCE*	Q15113	1.53E+07	5.59E+06
80	*TF*	P02787	1.52E+07	3.58E+06
81	*ITIH1*	P19827	1.47E+07	3.79E+06
82	*KRT16*	P08779	1.42E+07	1.41E+07
83	*SERPINF2*	P08697	1.37E+07	4.48E+06
84	*CCL14*	Q16627	1.17E+07	7.45E+06
85	*ITIH2*	P19823	1.15E+07	4.30E+06
86	*CP*	P00450	1.10E+07	5.03E+06
87	*CFI*	P05156	1.05E+07	3.63E+06
88	*CFHR2*	P36980	1.05E+07	2.01E+06
89	*ORM2*	P19652	1.02E+07	1.79E+06
90	*PF4*; *PF4V1*	P02776; P10720	1.00E+07	4.15E+06
91	*IGJ*	P01591	1.00E+07	2.13E+06
92	*KRT9*	P35527	9.93E+06	3.95E+06
93	*THBS1*	P07996	9.67E+06	2.12E+06
94	*C5*	P01031	9.48E+06	8.09E+05
95	*IGKV4-1*	P06312	9.47E+06	1.76E+06
96	*GPX3*	P22352	9.26E+06	6.98E+06
97	*KRT10*	P13645	9.17E+06	1.12E+07
98	*CTTN*	Q14247	8.81E+06	5.31E+05
99	*APOF*	Q13790	8.35E+06	7.57E+06
100	*KRT6A*; *KRT6C*	P02538	8.15E+06	1.28E+07
101	*SERPIND1*	P05546	7.97E+06	2.79E+06
102	*IGHV3-15*	A0A0B4J1V0	7.45E+06	1.47E+06
103	*F9*	P00740	7.34E+06	7.75E+05
104	*S100A8*	P05109	7.34E+06	4.51E+06

Supplemental Table 2. Ranked bound proteins identified and quantified by LC MS/MS

105	*SERPINA3*	P01011	7.10E+06	3.34E+06
106	*PFN1*	P07737	6.98E+06	1.87E+06
107	*IGHV4-61*; *IGHV4-4*	A0A0C4DH41; A0A075B6R2	6.93E+06	1.13E+06
108	*S100A9*	P06702	6.57E+06	1.61E+06
109	*APOL1*	O14791	6.48E+06	1.54E+06
110	*C4BPB*	P20851	5.79E+06	2.37E+06
111	*CD5L*	O43866	5.73E+06	1.55E+06
112	*IGF2*	P01344	5.68E+06	4.48E+06
113	*HPR*	P00739	5.58E+06	3.98E+06
114	*IGFBP3*	P17936	5.49E+06	3.31E+06
115	*C2*	P06681	5.24E+06	8.22E+05
116	*F5*	P12259	5.23E+06	1.12E+06
117	*AGT*	P01019	5.19E+06	1.97E+06
118	*A1BG*	P04217	4.67E+06	2.39E+06
119	*ACTB*; *ACTG1*	P60709; P63261	4.52E+06	1.75E+06
120	*PLTP*	P55058	4.48E+06	7.61E+04
121	*KRT2*	P35908	4.33E+06	3.05E+05
122	*GAPDH*	P04406	4.26E+06	1.05E+06
123	*BLVRB*	P30043	4.21E+06	6.93E+05
124	*SERPINA10*	Q9UK55	4.20E+06	2.31E+05
125	*HBB*	P68871	4.11E+06	1.26E+06
126	*IGHD*	P0DOX3	3.87E+06	9.96E+05
127	*HSPA5*	P11021	3.76E+06	8.31E+05
128	*HPX*	P02790	3.21E+06	2.59E+06
129	*FBLN1*	P23142	3.02E+06	8.34E+05
130	*C8B*	P07358	2.89E+06	7.75E+05
131	*CPN1*	P15169	2.71E+06	5.01E+05
132	*TAGLN2*	P37802	2.64E+06	1.27E+06
133	*CFL1*	P23528	2.46E+06	7.68E+05
134	*PROC*	P04070	2.42E+06	3.00E+05
135	*LPA*	P08519	2.41E+06	6.32E+05
136	*APMAP*	Q9HDC9	2.34E+06	5.97E+04
137	*ACTG2*; *ACTA1*; *ACTC1*; *ACTA2*	P63267; P68133; P68032; P62736	2.15E+06	2.65E+05
138	*LV321*	P80748	2.10E+06	3.92E+05

Supplemental Table 2. Ranked bound proteins identified and quantified by LC MS/MS

139	*IGHV3-23*	P01764	2.00E+06	5.68E+05
140	*IGHV1-3*; *IGHV1-69-2*	A0A0C4DH29; A0A0B4J2H0	1.93E+06	5.62E+05
141	*EFEMP1*	Q12805	1.90E+06	8.50E+05
142	*F11*	P03951	1.87E+06	6.57E+05
143	*FETUB*	Q9UGM5	1.84E+06	1.12E+06
144	*PROS1*	P07225	1.74E+06	3.44E+05
145	*IGA2*	P0DOX2	1.68E+06	3.22E+05
146	*PCYOX1*	Q9UHG3	1.65E+06	9.99E+05
147	*PRG4*	Q92954	1.63E+06	2.08E+05
148	*CRTAC1*	Q9NQ79	1.62E+06	8.23E+05
149	*LV319*	P01714	1.49E+06	3.55E+04
150	*CFHR4*	Q92496	1.11E+06	5.82E+05
151	*MST1*	P26927	1.01E+06	6.51E+05
152	*IGF1*	P05019	9.33E+05	5.25E+05
153	*IDH1*	O75874	8.87E+05	2.12E+05
154	*QSOX1*	O00391	8.03E+05	1.48E+05
155	*ITIH3*	Q06033	7.95E+05	2.51E+05
156	*MAN1A1*	P33908	7.88E+05	1.43E+04
157	*ALDOB*	P05062	7.54E+05	4.21E+05
158	*AFM*	P43652	6.24E+05	1.41E+05
159	*HSP90AA1*; *HSP90AB1*	P07900; P08238	6.13E+05	2.33E+05
160	*PGLYRP2*	Q96PD5	5.78E+05	5.14E+05
161	*GPLD1*	P80108	5.24E+05	2.38E+05
162	*CFP*	P27918	4.53E+05	4.25E+04
163	*APOA5*	Q6Q788	4.05E+05	2.98E+05
164	*TLN1*	Q9Y490	3.36E+05	2.00E+04
165	*ALDOA*	P04075	3.28E+05	2.20E+05
166	*TGFBI*	Q15582	3.23E+05	1.58E+05
167	*COL6A3*	P12111	2.66E+05	1.37E+05
168	*RB1CC1*	Q8TDY2	2.01E+05	9.30E+04
169	*ADAMTSL4*	Q6UY14	1.91E+05	1.71E+05
170	*HSPA8*	P11142	1.73E+05	6.73E+04
171	*AHNAK*	Q09666	1.63E+05	1.57E+05
172	*LUM*	P51884	1.13E+05	2.40E+04
173	*MMRN1*	Q13201	7.22E+04	7.55E+03
174	*SSC5D*	A1L4H1	7.11E+04	1.61E+04

Supplemental Table 3. Ranked released proteins identified and quantified by LC MS/MS

	Gene Symbols	UniProt protein ID	iBAQ AV (n=3)	iBAQ SD (n=3)
**1**	***APOA1***	P02647	8.97E+09	2.85E+09
**2**	***FGG***	P02679	3.09E+09	8.28E+08
**3**	***FGA***	P02671	2.51E+09	5.40E+08
**4**	***FGB***	P02675	2.02E+09	4.78E+08
**5**	***APOA2***	P02652	1.65E+09	8.30E+08
**6**	***APOH***	P02749	9.82E+08	3.25E+07
**7**	***APCS***	P02743	6.94E+08	2.56E+08
**8**	***APOC3***	P02656	6.76E+08	7.77E+07
**9**	***APOA4***	P06727	6.46E+08	2.44E+08
**10**	***ALB***	P02768	5.44E+08	8.26E+07
**11**	***IGHG1***	P01857	4.52E+08	8.07E+07
**12**	***CFH***	P08603	3.80E+08	9.70E+07
**13**	***CLU***	P10909	3.69E+08	6.68E+07
**14**	***C3***	P01024	2.91E+08	6.92E+07
**15**	***SERPINF1***	P36955	2.41E+08	6.18E+07
**16**	***AHSG***	P02765	2.18E+08	3.48E+07
**17**	***PLG***	P00747	2.09E+08	4.54E+07
**18**	***CRP***	P02741	1.97E+08	1.04E+08
**19**	***C4B***	P0C0L5	1.79E+08	5.52E+07
**20**	***SERPING1***	P05155	1.43E+08	2.51E+07
21	*HP*	P00738	1.39E+08	3.08E+07
22	*IGHA1*	P01876	1.38E+08	3.40E+07
23	*IGHG3*	P01860	1.37E+08	4.50E+07
24	*PON1*	P27169	1.37E+08	5.36E+07
25	*GSN*	P06396	1.37E+08	3.88E+07
26	*IGHM*	P01871	1.16E+08	3.59E+07
27	*C8G*	P07360	1.14E+08	3.25E+07
28	*APOC2*	P02655	9.88E+07	2.15E+07
29	*APOD*	P05090	9.79E+07	6.30E+07
30	*IGLL5*; *IGLC1*	B9A064; P0CG04	9.62E+07	4.65E+07
31	*C1QB*	P02746	9.51E+07	2.65E+07

Supplemental Table 3. Ranked released proteins identified and quantified by LC MS/MS

32	*C8A*	P07357	9.22E+07	3.05E+07
33	*ITIH4*	Q14624	7.66E+07	1.73E+07
34	*IGK*	P0DOX7	7.57E+07	1.47E+07
35	*C4BPA*	P04003	7.41E+07	4.51E+07
36	*C9*	P02748	7.00E+07	1.69E+07
37	*KNG1*	P01042	6.84E+07	6.21E+06
38	*APOC1*	P02654	6.77E+07	5.54E+07
39	*IGHG2*	P01859	6.71E+07	9.30E+06
40	*C1R*	P00736	6.28E+07	1.45E+07
41	*KLKB1*	P03952	6.23E+07	1.71E+07
42	*VTN*	P04004	6.15E+07	1.20E+07
43	*HRG*	P04196	6.03E+07	1.42E+07
44	*C1S*	P09871	5.80E+07	1.75E+07
45	*C1QC*	P02747	5.33E+07	1.23E+07
46	*KRT1*	P04264	5.00E+07	3.10E+07
47	*SERPINA1*	P01009	4.21E+07	1.17E+07
48	*APOE*	P02649	3.95E+07	6.07E+06
49	*APOM*	O95445	3.91E+07	2.66E+07
50	*AMBP*	P02760	3.84E+07	1.15E+07
51	*SEPP1*	P49908	3.76E+07	6.42E+06
52	*TTR*	P02766	3.52E+07	5.26E+06
53	*CP*	P00450	3.47E+07	9.83E+06
54	*KV320*	P01619	3.41E+07	1.10E+07
55	*CFHR2*	P36980	3.09E+07	1.59E+06
56	*FN1*	P02751	2.97E+07	8.57E+06
57	*TF*	P02787	2.94E+07	6.02E+06
58	*F12*	P00748	2.94E+07	7.62E+06
59	*SERPINC1*	P01008	2.78E+07	4.77E+06
60	*CFI*	P05156	2.72E+07	8.26E+06
61	*C1QA*	P02745	2.71E+07	6.26E+06
62	*GC*	P02774	2.51E+07	2.81E+06
63	*CFB*	P00751	2.44E+07	6.97E+06
64	*SERPINA5*	P05154	2.38E+07	8.77E+06
65	*A2M*	P01023	2.38E+07	6.66E+06
66	*SAA4*	P35542	2.36E+07	9.38E+06

Supplemental Table 3. Ranked released proteins identified and quantified by LC MS/MS

67	*C7*	P10643	2.36E+07	6.36E+06
68	*TMSB4X*	P62328	2.24E+07	1.34E+07
69	*CFHR5*	Q9BXR6	2.15E+07	3.68E+06
70	*IGJ*	P01591	2.11E+07	2.43E+06
71	*C6*	P13671	2.11E+07	4.32E+06
72	*PPBP*	P02775	1.90E+07	4.60E+06
73	*LV147*	P01700	1.87E+07	5.06E+06
74	*SERPINA3*	P01011	1.78E+07	6.52E+06
75	*KRT9*	P35527	1.77E+07	2.01E+07
76	*IGKV A18*; *IGKV2D-26*; *IGKV2D-29*	A2NJV5; A0A0A0MRZ7; A0A075B6S2	1.75E+07	4.17E+06
77	*ORM2*	P19652	1.71E+07	3.23E+06
78	*S100A8*	P05109	1.64E+07	5.09E+06
79	*FBLN1*	P23142	1.52E+07	2.94E+06
80	*SERPINA4*	P29622	1.35E+07	4.60E+06
81	*KRT16*	P08779	1.34E+07	5.84E+06
82	*S100A9*	P06702	1.29E+07	3.78E+06
83	*IGHV3-15*	A0A0B4J1V0	1.29E+07	4.14E+06
84	*IGHV4-61*; *IGHV4-4*	A0A0C4DH41; A0A075B6R2	1.28E+07	3.85E+06
85	*KRT10*	P13645	1.27E+07	9.45E+05
86	*APOB*	P04114	1.20E+07	4.07E+06
87	*ITIH1*	P19827	1.19E+07	1.15E+07
88	*ITIH2*	P19823	1.18E+07	9.74E+06
89	*C4BPB*	P20851	1.15E+07	2.05E+06
90	*HPX*	P02790	1.15E+07	4.33E+06
91	*IGKV4-1*	P06312	1.05E+07	3.17E+06
92	*SERPINF2*	P08697	9.68E+06	6.26E+06
93	*CD5L*	O43866	9.44E+06	1.04E+06
94	*KRT6A*; *KRT6C*	P02538; P48668	9.04E+06	1.12E+07
95	*GPX3*	P22352	8.51E+06	1.59E+06
96	*QSOX1*	O00391	7.96E+06	1.01E+06
97	*APOF*	Q13790	7.89E+06	6.56E+06
98	*HPR*	P00739	7.38E+06	1.77E+06
99	*C5*	P01031	7.27E+06	1.92E+06
100	*CLEC3B*	P05452	7.10E+06	4.35E+06

Supplemental Table 3. Ranked released proteins identified and quantified by LC MS/MS

101	*ACTB*; *ACTG1*	P60709; P63261	6.96E+06	1.38E+06
102	*KRT2*	P35908	6.91E+06	3.27E+06
103	*CFHR4*	Q92496	6.88E+06	1.07E+06
104	*CTTN*	Q14247	6.79E+06	2.26E+06
105	*CFD*	P00746	6.68E+06	2.87E+06
106	*SAA1*	P0DJI8	6.66E+06	1.53E+06
107	*IGHD*	P0DOX3	6.33E+06	2.42E+06
108	*AGT*	P01019	6.33E+06	1.62E+06
109	*A1BG*	P04217	6.06E+06	2.10E+06
110	*C8B*	P07358	5.98E+06	1.48E+06
111	*IGHV1-18*	A0A0C4DH31	5.71E+06	2.41E+06
112	*APOL1*	O14791	5.58E+06	3.16E+06
113	*PLTP*	P55058	5.52E+06	2.65E+06
114	*F2*	P00734	5.28E+06	2.01E+06
115	*HABP2*	Q14520	4.84E+06	1.35E+06
116	*HBB*	P68871	4.69E+06	1.38E+06
117	*LBP*	P18428	4.14E+06	3.63E+06
118	*IGLV3-10*	A0A075B6K4	4.05E+06	2.24E+06
119	*PGLYRP2*	Q96PD5	3.94E+06	1.67E+06
120	*LV321*	P80748	3.90E+06	1.50E+05
121	*SERPIND1*	P05546	3.70E+06	1.65E+06
122	*IGA2*	P0DOX2	3.63E+06	6.94E+05
123	*IGHV3-23*	P01764	3.60E+06	5.77E+05
124	*F10*	P00742	3.59E+06	2.21E+06
125	*LV319*	P01714	3.32E+06	1.16E+06
126	*IGHV1-3*; *IGHV1-69-2*	A0A0C4DH29; A0A0B4J2H0	3.18E+06	1.06E+06
127	*EFEMP1*	Q12805	3.17E+06	6.64E+05
128	*TADA2B*	Q86TJ2	3.04E+06	2.26E+06
129	*IGHV3-74*; *IGHV3-66*	A0A0B4J1X5; A0A0C4DH42	2.88E+06	7.82E+05
130	*KRT14*	P02533	2.78E+06	2.00E+06
131	*IGKV2-24*	A0A0C4DH68	2.69E+06	8.22E+05
132	*CST3*	P01034	2.55E+06	3.37E+05
133	*MST1*	P26927	2.51E+06	1.12E+06
134	*SERPINA10*	Q9UK55	2.45E+06	4.97E+05
135	*KRT5*	P13647	2.33E+06	2.36E+06

Supplemental Table 3. Ranked released proteins identified and quantified by LC MS/MS

136	*AFM*	P43652	2.27E+06	7.52E+05
137	*PROS1*	P07225	2.19E+06	6.50E+05
138	*IGFBP3*	P17936	2.18E+06	1.62E+06
139	*APMAP*	Q9HDC9	2.11E+06	9.18E+05
140	*CPN2*	P22792	2.05E+06	7.49E+05
141	*FCN3*	O75636	1.64E+06	5.17E+05
142	*CRTAC1*	Q9NQ79	1.55E+06	4.59E+05
143	*ACTG2*; *ACTA1*; *ACTC1*; *ACTA2*	P63267; P68133; P68032; P62736	1.52E+06	3.09E+05
144	*F5*	P12259	1.45E+06	2.36E+05
145	*PRSS3*	P35030	1.33E+06	3.08E+05
146	*CFP*	P27918	1.23E+06	3.31E+05
147	*PCYOX1*	Q9UHG3	1.07E+06	6.77E+05
148	*F11*	P03951	1.06E+06	4.30E+05
149	*GPLD1*	P80108	1.02E+06	3.43E+05
150	*FGL1*	Q08830	1.02E+06	3.72E+05
151	*IGFALS*	P35858	1.01E+06	4.86E+05
152	*LPA*	P08519	1.01E+06	3.85E+05
153	*IGHV5-51*	A0A0C4DH38	1.00E+06	2.92E+05
154	*SERPINE1*	P05121	7.80E+05	5.18E+05
155	*TGFBI*	Q15582	6.54E+05	6.35E+05
156	*ECM1*	Q16610	6.53E+05	3.27E+05
157	*ITIH3*	Q06033	5.98E+05	2.53E+05
158	*C2*	P06681	5.22E+05	3.40E+05
159	*COMP*	P49747	3.92E+05	5.19E+05
160	*COLEC11*	Q9BWP8	2.56E+05	4.80E+04
161	*SSC5D*	A1L4H1	2.04E+05	2.06E+04
162	*ZGRF1*	Q86YA3	1.90E+05	1.12E+04
163	*COL6A3*	P12111	1.82E+05	1.67E+05
164	*THBS1*	P07996	6.19E+04	1.15E+04
165	*TLN1*	Q9Y490	5.20E+04	3.70E+04

Supplemental Table 4. Plasma proteins unbound to LC-CSH

Gene Symbols	UniProt protein ID
ATRN	O75882
AZGP1	P25311
BTD	P43251
CD14	P08571
CPB2	Q96IY4
CPN2	P22792
F13B	P05160
FCGBP	Q9Y6R7
FCN3	O75636
HBA1	P69905
IGFALS	P35858
KVD39	P04432
LGALS3BP	Q08380
LRG1	P02750
MASP1	P48740
MBL2	P11226
ORM1	P02763
PGLYRP2	Q96PD5
RBP4	P02753
SERPINA4	P29622
SERPINA6	P08185
SERPINA7	P05543
VWF	P04275
ZGRF1	Q86YA3

Supplemental Table 5. Clinical characteristics of clinical study Ib subjects

Parameter	Cohort, n=73
**Demographics and medical history**	
Male, n (%)	37 (50.7)
Age (years)	60.15 ± 7.61
Body mass index (kg/m^2)	28.97 ± 5.86
Systolic blood pressure (mmHg)	115.7 ± 15.0
Diastolic blood pressure (mmHg)	62.6 ± 10.6
Lipid lowering therapy, n (%)	37 (50.7)
Current Smoker, n (%)	6 (8.3)
DM2	14 (19.4)
PCI / CABG	45 (61.6)
**Race**	
White	48 (65.8)
Black or African American	14 (19.2)
Asian or unknown	11 (15.1)
**CADRADS classification**	
CADRADS 0, n (%)	26 (35.6)
CADRADS 1, n (%)	25 (34.2)
CADRADS 2, n (%)	22 (30.1)
**Clinical and laboratory values**	
high sensitivity C-reactive protein (mg/L)	1.20 (0.60-2.30)
Total cholesterol (mg/dl)	183.74 ± 30.41
LDL cholesterol (mg/dl)	96.08 ± 29.69
HDL cholesterol (mg/dl)	59.78 ± 18.18
Triglycerides (mg/dl)	112 (72.0, 157.0)
ApoA-I (mg/L)	167.5 ± 28.35
ApoB (mg/L)	89.99 ± 21.74
Agatston score	8.00 (0, 71.0)
**HDL function parameter**	
Cholesterol efflux capacity	1.02 ± 0.17
HDL-SPE	0.99 ± 0.13
**CCTA plaque parameter**[a]	**n=208 arteries**
Total plaque burden (mm^2)[b]	0.0108 ± 0.0036
Non-calcified plaque burden (mm^2)	0.0106 ± 0.0036
Dense-calcified plaque burden (mm^2)	4.4×10^{-5} (1.4×10^{-5}, 1.3×10^{-4})
Necrotic Burden (mm^2)	3.1×10^{-5} (1.3×10^{-5}, 7.1×10^{-5})
Fibro-Fatty burden (mm^2)	5.9×10^{-4} (3.0×10^{-4}, 1.0×10^{-3})
Fibrous burden (mm^2)	0.0048 ± 0.0019

Supplemental Table 5. Clinical characteristics of clinical study Ib subjects

Data represented as mean ± standard deviation or median (IQR) for parametric and non-parametric variables and as n (%) for categorical variables. HDL: high density lipoprotein, LDL: low density lipoprotein, ApoA-I: apolipoprotein A-I, ApoB: apolipoprotein B, HDL-SPE: HDL-specific phospholipid efflux. CCTA: coronary computed tomography angiography. (a), 208 plaque data of three coronary arteries in each subject; right coronary artery (RCA) and/or left anterior descending coronary artery (LAD) and/or left circumflex coronary artery (LCX). (b), Total plaque burden (TB) consists of non-calcified plaque (NCB) and dense-calcified plaque (DCB).

Supplemental Table 6. Comparison of HDL-SPE to related assays

	Representative reference	Mechanism	Metric	Requirements			Additional requirements	Quantifiability	Suitable for automation	Evaluated with CVD patients
				ApoB-depletion (or HDL isolation)	Cell culture	Radioactive				
Cell-free	**HDL-SPE**	Phospholipid efflux capacity	Fluorescent-Phosphatidyl-ethanolamine	NO	NO	NO	NO	YES	YES	YES
	Neufeld, E.B. *et al.* (2019)	Phospholipid efflux capacity	Fluorescent-Phosphatidyl-ethanolamine	NO	NO	NO	Agarose gel electrophoresis	Limited*	NO	NO
	Harada, A. *et al.* (2017)	Free-Cholesterol uptake capacity	Fluorescent-Cholesterol	YES	NO	NO	・Serum apoAI measurement<br>・Anti-apoAI antibody incubation	YES	YES	YES
	Horiuchi, Y. *et al.* (2018)	Cholesterol efflux capacity	Fluorescent-Cholesterol	YES	NO	NO	NO	YES	YES	NO
	Borja, M.S. *et al.* (2013)	ApoAI exchange-ability	Spin-labeled apoAI†	YES	NO	NO	NO	YES	?	YES
	Lorkowski, S.W. *et al.* (2020)	ApoAI exchange rate	NBD/Alexa647 labeled apoA-I fluorescence kinetics	NO	NO	NO	Kinetic fluorescence measurement	YES	YES	YES
Cell-based	Rohatgi, A. *et al.* (2014)	Cholesterol efflux capacity	Fluorescent-Cholesterol‡	YES‡	YES	NO‡	・ABCA1 induction<br>・Cell lysis	YES	NO	YES
	Remaley A.T. *et al.* (2001)	Phospholipid efflux capacity	Radioactive-Phosphatidyl-choline and sphingomyelin	YES	YES	YES	・ABCA1 induction<br>・Cell lysis<br>・Phospholipid extraction	YES	NO	NO

*Relative intensity, but not % efflux efficiency, can be quantitative.

†Measured using electron paramagnetic resonance spectroscopy.

‡These are inconsistent between researchers.
